# SARS-CoV-2 infection risk by non-healthcare occupations: a systematic review and meta-analysis

**DOI:** 10.1186/s12995-025-00462-9

**Published:** 2025-05-22

**Authors:** Katharina M. A. Gabriel, Christin Schröder, Rebecca Wolf, Ulrich Bolm-Audorff, Camilla Kienast, Joanna Smolinska, Gabriela Petereit-Haack, Andreas Seidler

**Affiliations:** 1 Division ‘Work and Health’, Unit ‘Medical Occupational Safety and Health, Occupational Diseases’, Federal Institute for Occupational Safety and Health, Nöldnerstr. 42, Berlin, 10317 Germany; 2 Faculty of Medicine, Technische Universität Dresden, Institute and Policlinic of Occupational and Social Medicine (IPAS), Fetscherstraße 74, Dresden, 01307 Germany; 3 Center for Occupational Medical Care, Charité - Universitaetsmedizin Berlin, Turmstraße 21, Berlin, 10559 Germany; 4 Division of Occupational Health, Department of Occupational Safety, Regional Government of South Hesse, Kreuzberger Ring 17, Wiesbaden, 65205 Germany

**Keywords:** COVID-19, Global, Meta-analysis, Occupational, Work

## Abstract

**Background:**

During the COVID-19 pandemic, several industries were deemed essential. However, information on infection risk in occupational settings outside of healthcare workers and medical staff (HCWs) remain scarce. Thus, a systematic review with meta-analysis was conducted to compile the risk of infection to SARS-CoV-2 in non-healthcare workers (non-HCWs).

**Methods:**

We screened three databases (EMBASE, PubMed, medRχiv) for studies on SARS-CoV-2 infection risk in working population. Several stages of severity (infection, hospitalisation, admission to intensive care unit (ICU), mortality) were eligible. Occupational specifications were harmonised according to the German classification of professions (KldB). All reported risk estimators were considered. Studies were analysed for their risk of bias. Results of random-effects meta-analyses were assessed for their evidence according to GRADE. Subgroup analyses were run for ‘outcome’, ‘comparison group’, and ‘risk of bias’.

**Results:**

Of 9,081 publications identified, 25 were recognised as eligible, mainly describing the first year of the pandemic. For 20 occupations, we were able to carry out meta-analyses on KldB-4-level by integrating all stages of severity. Nine occupations were identified with a statistically significantly increased risk of infection for SARS-CoV-2, four of which had a relative risk (RR) of > 2: Occupations in meat processing (RR = 3.58 [95%-CI 1.46; 8.77]), occupations in building cleaning services (RR = 2.55 [95%-CI 1.51; 4.31]), occupations in cargo handling (RR = 2.52 [95%-CI 2.27; 2.79]) and cooks (RR = 2.53 [95%-CI 1.75; 3.67]). The certainty of evidence of eight results was found moderate or high.

**Conclusions:**

The first systematic review and meta-analysis of occupational SARS-CoV-2 infection risk in occupations other than HCWs revealed a considerably elevated risk in individual related services as well as in commercial services.

**Trial registration:**

PROSPERO CRD42021297572.

**Supplementary Information:**

The online version contains supplementary material available at 10.1186/s12995-025-00462-9.

## Abstrakt

**Hintergrund:**

Während der COVID-19-Pandemie wurden mehrere Branchen als systemrelevant angesehen. Außerhalb der Berufsgruppe der Beschäftigten im Gesundheitswesen und dem medizinischen Personal liegen jedoch nur wenige Informationen über das Infektionsrisiko vor. Daher wurde ein systematisches Review mit Meta-Analyse durchgeführt, um das Infektionsrisiko für SARS-CoV-2 in den übrigen Berufen zu ermitteln.

**Methodik:**

Drei Datenbanken (EMBASE, PubMed, medRχiv) wurden nach Studien zum SARS-CoV-2-Infektionsrisiko in der arbeitenden Bevölkerung durchsucht. Mehrere Stadien der Erkrankung (Infektion, Krankenhausaufenthalt, Einweisung auf die Intensivstation (ICU), Sterblichkeit) wurden berücksichtigt. Die angegebenen Berufe wurden gemäß der deutschen Klassifikation der Berufe (KldB) harmonisiert. Alle berichteten Risikoschätzer wurden berücksichtigt. Die Studien wurden auf ihr Verzerrungsrisiko hin analysiert. Die Ergebnisse der Meta-Analysen wurden nach GRADE hinsichtlich ihrer Evidenz bewertet. Subgruppenanalysen wurden durchgeführt für ‚outcome‘, ‚Vergleichsgruppe‘ (‚comparison group‘) sowie für ‚Risk of Bias‘.

**Ergebnisse:**

Von 9.081 gefundenen Texten wurden 25 als geeignet eingestuft, wobei sie vorwiegend das erste Jahr der Pandemie beschrieben. Für 20 Berufe konnten Meta-Analysen auf KldB-4-Ebene durchgeführt werden. Neun Berufe wurden mit einem statistisch signifikant erhöhten Infektionsrisiko für SARS-Cov-2 identifiziert, vier davon mit einem Relativen Risiko (RR) von > 2: Berufe in der Fleischverarbeitung (RR = 3,58 [1,46; 8,77]), Berufe in der Gebäudereinigung (RR = 2,55 [1,51; 4,31]), Berufe im Frachtumschlag (RR = 2,52 [2,27; 2,79]) und Köche (RR = 2,53 [1,75; 3,67]). Die Evidenz von acht Ergebnissen wurde als mäßig oder hoch eingestuft.

**Fazit:**

Das erste systematische Review mit Meta-Analyse zum beruflichen SARS-CoV-2-Infektionsrisikos in Berufen außerhalb des Gesundheitswesens ergab ein deutlich erhöhtes Risiko in personenbezogenen Dienstleistungen sowie in gewerblichen Dienstleistungen.

## Introduction

The SARS-CoV-2 pandemic challenged the international community. Healthcare workers and medical staff were at the forefront of the response. Their increased risk of acquiring an infection themselves was studied intensively (e.g., [1]). The occupational context of their infection was acknowledged by the recognition of SARS-CoV-2 infection as an occupational disease for healthcare workers (HCWs) in many countries [2, 3].

As SARS-CoV-2 was airborne, many countries imposed lockdowns to prevent the spread of the virus. Many people worked from home or stopped working altogether. However, several industries were considered as essential [4] and their workers often could not be transferred to home-office (i.e., *frontline workers*) [5]. This suggests that, in addition to healthcare workers, other occupational groups may be at risk of increased potential regular exposure to the virus, especially those who cannot keep their distance.

Although the risk of infection in the workplace has been recognised by policymakers, general studies on the subject have been relatively scarce and selective. Therefore, a systematic review with meta-analysis was conducted to examine the risk of infection with SARS-CoV-2 in occupations other than healthcare workers and medical staff.

## Methods

The approach of the analysis was described and announced a priori in a PROSPERO-protocol (CRD42021297572) [6]. Its reporting follows the PRISMA guideline [7, 8].

### Search and selection

Following the PECOS-scheme – addressing population, exposure, comparison, outcome, and study type, we defined the working population (i.e., between 15 and 65 years of age) as the population of interest. Occupations other than healthcare workers were defined as eligible exposure. Since in a pandemic there are hardly any unexposed groups, we graded the comparator in descending order as (1) administrative occupations (as in general, they were able to work from home), (2) non-essential occupations (i.e., complementing the essential occupations, such as HCWs, bus drivers and police officers), (3) (all) other occupations, and (4) the general population. The outcome was defined as either a positive test for SARS-CoV-2 or an ICD-10 code (U07.1) recorded in the data source as reported on a medical certificate, as a hospital diagnosis or as a stated reason on a death certificate. As eligible study designs, cross-sectional and case-control studies as well as cohorts were accepted.

Search for eligible literature was conducted in three databases on February 1st 2022: in EMBASE, in Medline via PubMed and in medRχiv. For each database, we adopted the search string according to their condition (Appendix A.1). The search was not limited to any languages but to a date of publication from January 1st, 2020. This can be seen as the onset of the current pandemic, giving a clear distinction to the first SARS-pandemic in 2002/2003 and to the MERS-epidemic following 2012. A search beyond the mentioned three databases was not conducted actively, however, recommendations of peers were considered if the publication was available within the search period as well.

After removing duplicates, titles and abstracts of the resulting publications were screened for eligibility. A pilot was run to align the evaluation within the team. Then, the rest was evaluated in teams of two (among CK, CS, JS, RW, KG) independently. Publications disagreed about initially were discussed and, in case of no agreement, passed to a third scientist (AS) for decision. Full texts were retrieved and further screened for eligibility in teams of two (among CK, CS, RW, KG). Results were discussed and further refined (with UBA). Beforehand, articles in languages other than English or German were translated by professional translators.

Studies were eligible when reporting occupations other than or in addition to healthcare workers. We accepted occupations of all industries and permitted all occupational descriptions, whether following an official coding system or not. Studies selected for inclusion had to provide one of the four comparators, whereas those referring to HCWs were not eligible. Reasons for excluding full texts were specified as one of eight categories, following the predefined exclusion criteria (population, exposure, comparison, outcome, study design) as well as expanding to data quality (response, occupation) and an open category (else) (see Appendix A.2 for full definition). Thereby, focus was on the data’s adequate presentation, complemented by methodological aspects.

### Quality assessment

For assessing the risk of bias (RoB) on publication level, we applied a total of ten domains on each publication (adapted from [9]).

- Major domains were:
  (i) acceptable recruitment procedure, for what a distinct source population for cohorts and cross-sectional studies as well as controls in case control studies were required; baseline response was equal or above 50% or it was between 50% and 30%, but substantial differential selection could be excluded; in case of cohort studies, loss of follow-up was below 20% in total and did not differ between the two groups more than 10%.
  (ii) sufficiently precise exposure definition and assessment,
  (iii) objectively measured outcome,
  (iv) adequate consideration of confounding and effect modification,
  (v) use of adequate analysis methods (e.g., standardization, adjustment in multivariate model).
- Minor domains of bias included:
  (i) chronology,
  (ii) blinding of assessors,
  (iii) selective reporting,
  (iv) funding,
  (v) conflict of interest.

Due to a comparatively short incubation period of COVID-19 and because the assessment of occupation as exposure may be simultaneous to that of the outcome, chronology was not considered a major but a minor domain.

In order for a study to have an overall low RoB, every major domain had to be rated as low risk. Risk of bias assessment was performed independently in teams of two (among CK, CS, RW, KG) and discussed between all authors.

### Data extraction and occupational classification

At the publication level, basic information on authors and year of publication was collected, followed by information on the type of study as well as on the study population (if available, age, sex, and country of origin). Reported results were identified together with the type of their modality of COVID-19-confirmation. Furthermore, confounding variables considered in the respective study were listed.

At the level of the datasets, both the occupational description and the associated risk estimator were extracted in order to describe the respective occupational risk of infection. If there were results reported on more than one level of occupational description, the most detailed was preferred for extraction. Occupational information in the data sets was harmonised according to the German classification of professions (KldB), which follows a topical scheme of grouping [10, 11]. Each data set was assigned to one of ten occupational fields (KldB-level-1) and then further refined to KldB-level-4, if possible. While KldB-level-2 describes major occupational groups, KldB-level-3 and -4 signify main and minor occupational groups. Thus, a data set is classified at all levels above the most detailed one.

All reported risk estimators were considered (e.g., Odds Ratio, Hazard Ratio, Risk Ratio, Standardized Incidence Ratio, Standardized Mortality Ratio). If possible, adjusted estimators were extracted.

Data were extracted in teams of two independently (KG, CS, RW) and discussed until an agreement was reached.

### Comparison group

We predefined occupations as well as population groups as possible comparators. As first choice, we set ‘administrative occupations’, as we assumed high potential for working from home and, thus, the lowest occupational exposure. This was followed in descending order of preference by ‘non-essential workers’ and ‘others’ (a group which might contain HCWs); the least favoured comparison group was ‘general population’, as it contains the occupation of interest. Within each study, the best comparator available was chosen out of these four options.

### Data handling

In case of overlapping geographical areas and time periods between studies, we preferred those with a wider coverage and more recent information on occupation.

When overlapping periods within one study occurred, we preferred data sets of the first period (fewer COVID-19-pandemic restrictions) to the second (new restrictions set up).

### Meta-analyses and subgroup analyses

The extracted data was processed and converted in preparation for the quantitative analysis. Hazard ratios and odds ratios were considered as relative risk (RR). There was no violation of the rare disease assumption in any study, i.e., the cumulative incidence did not exceed 20% [12] in any study in the total population. Where no effect estimates were available or the comparator used to calculate the risk estimator was not eligible (e.g., HCWs), an RR was calculated from the data given within the studies. When multiple occupations from a single study were assigned to the same four-digit KldB number, a multilevel model was used [13], with one level representing the study level and the other the data set. This allows correlated effect sizes from a study to be taken into account, resulting in more robust and reliable estimates.

Due to the heterogeneity of the populations analysed in the studies and the local differences in the incidence of infection in the pandemic situation, a random-effects meta-analysis of the estimates was performed to obtain a pooled effect size for the risk of being infected with SARS-CoV-2 depending on the occupation. Calculations were done, if there were at least three data sets of the same number on KldB-level 4 from at least two different studies.

Heterogeneity was assessed by I^2^ values [14]. Publication bias was explored using Funnel plots and Egger’s test if there were at least ten studies available. Subgroup analyses were conducted for ‘outcome’, ‘comparator’ and ‘risk of bias’.

### GRADE – assessment of the certainty of evidence

Certainty of evidence of a positive or negative effect of occupation on infection risk was rated for every result describing an exposure-outcome relationship [15, 16], i.e., for every occupation for which a meta-analysis was possible. Each result was judged according to eight domains: five domains (‘risk of bias’, ‘inconsistency’, ‘indirectness’, ‘imprecision’ and ‘publication bias’) can result in a downgrade of up to two steps, while ‘effect size’, ‘dose-response relationship’ as well as a potential ‘under-estimation’ can result in an upgrade (further details in Appendix A.3). As initial level, ‘moderate evidence’ was assigned because potentially considerable unknown confounding was suspected due to high relevance of non-work-related risk factors for SARS-CoV-2.

## Results

### Search results

**Fig. 1:**
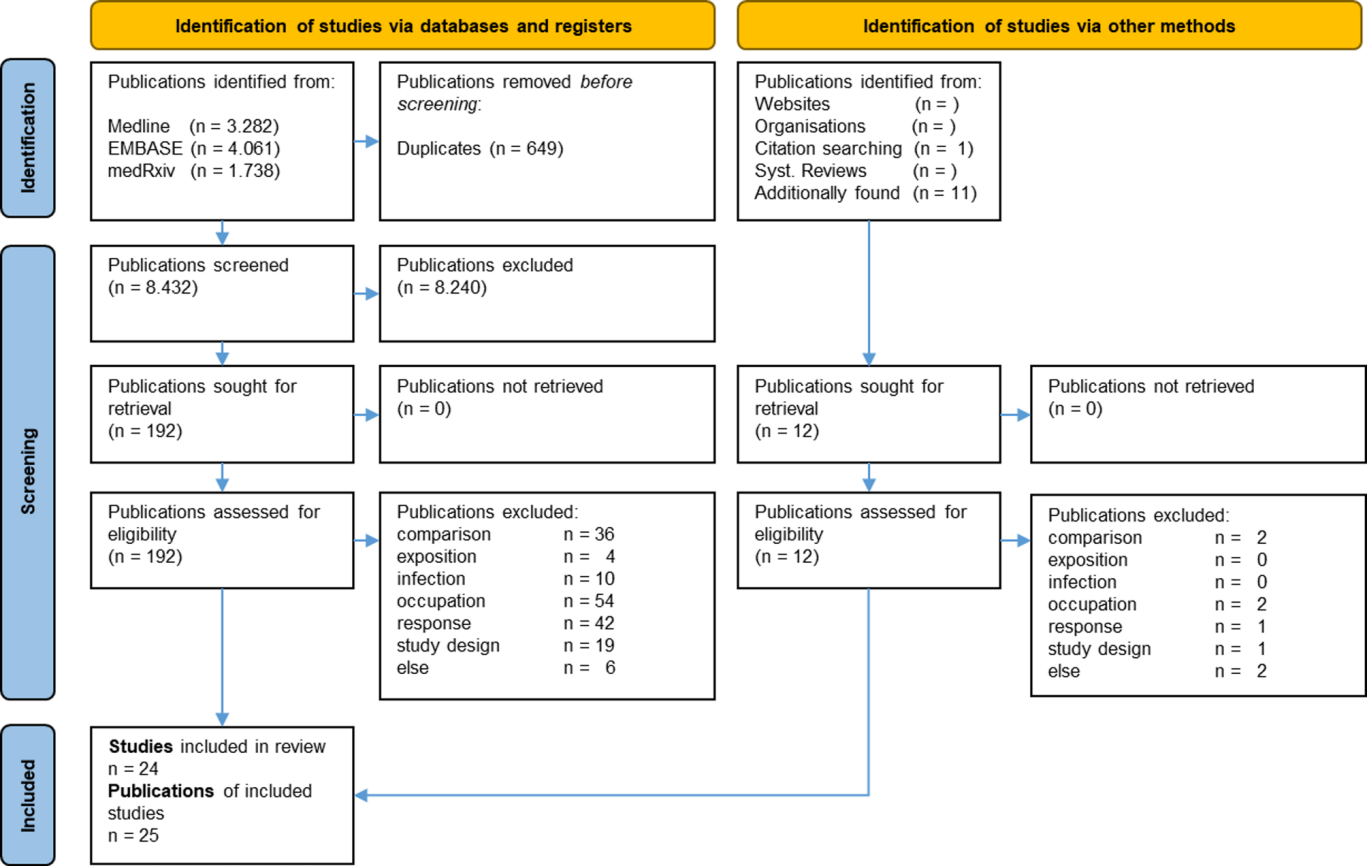
Flowchart

The search resulted in a total of 9,081 publications (Fig. 1). After removing duplicates (*n* = 649), 8,432 publications remained. Screening of title/abstract reduced eligible papers to 192, further 171 were eliminated by full text screening (details in Appendix A.2). An insufficient description of occupation was the main reason for exclusion (*n* = 54), followed by a non-satisfying information on the study’s response (*n* = 42) and a non-existing comparison group (*n* = 36). An inadequate study design (*n* = 19) and missing number of infected persons (*n* = 10) were subordinated reasons for exclusion.

The remaining 21 publications [17–37] were supplemented by four additionally found publications [38–41], resulting in a final compilation of 25 publications (Table 1).

**Table 1:** Characteristics of 25 eligible texts: (a) general information and study population, (b) exposure, (c) outcome

a)	General information				Study population					
	Country	Study type	Response rate	Risk of bias	Total	Men		Age		
						n	%	Description	Point value	Range
Al-Thani et al. 2021 [17]	QAT	cross-sec	>90.0%	high	2,641	2,641	100.0%	median (range)	35	18-80
Billingsley et al. 2022 [18]	SWE	cohort	100.0%	low	4,620,395	2,326,120	50.3%	meanClasses	43.2	---
Campbell et al. 2021 [19]	CAN	cross-sec	90.6%	high	2,128	1,320	62.0%	median (IQR)	48	37-57
Cummings et al. 2021 [20]	USA-CA	cohort	100.0%	high	6,607	5,039	76.3%	meanClasses	50.5	---
Della Valle et al. 2021 [38]	ITA	cohort	75.8%	high	2,255	1,569	69.6%	mean (SD)	44.5	9,71
Dìaz-Salazar et al. 2021 [21]	MEX	cross-sec	77.4%	high	3,268	2,008	61.4%	median (IQR)	40	31-49
Finkenzeller et al. 2020 [22]	DEU	cross-sec	67.7%	high	2,867	1,099	38.9%	meanClasses	42	---
Fogh et al. 2021 [23]	DNK	cohort	24.5%	high	318,552	114,424	35.9%	multiple median	---	---
Hawkins et al. 2021 [24]	USA-MA	cohort	100.0%	high	3,381,336	1,704,327	50.4%	meanClasses	56.2	---
House et al. 2021 [39]	USA-IA	cohort	56.5%	high	582	288	49.5%	median (IQR)	42	29-55
IIAC 2021 [40]	GBR	cohort	100.0%	low	33,194,305	16,331,210	49.2%	---	---	---
Lastrucci et al. 2020 [25]	ITA	cross-sec	95.5%	high	4,656	1,532	32.9%	median (IQR)	49	38-57
Magnusson et al. 2021 [26]	NOR	cohort	100.0%	low	3,579,608	1,825,600	51.0%	mean (SD)	44.1	14,3
Miller et al. 2021 [27]	USA-WA	cohort	75.5%	high	3,739	---	---	---	---	---
Möhner & Wolik 2020 [28]	DEU	cohort	100.0%	high	4,100,000	---	---	---	---	---
Möhner & Wolik 2021 [41]	DEU	cohort	100.0%	high	4,100,000	---	---	---	---	---
Nafilyan et al. 2021 [29]	GBR	cohort	81.6%	low	14,295,900	6,964,839	48.7%	mean (SD)	52.2	6,96
Nwaru et al. 2022 [30]	SWE	cohort	100.0%	low	326,052	162,143	49.7%	mean (SD)	43	12,5
Poustchi et al. 2021 [31]	IRN	cross-sec	97.0%	high	8,902	5,059	56.8%	meanClasses	42.6	---
Stringhini et al. 2021 [32]	CHE	cross-sec	41.0%	high	10,513	4,665	44.4%	mean (SD)	43	10,7
Stufano et al. 2021 [33]	ITA	cross-sec	91.3%	high	367	263	71.7%	multiple median	---	---
Tovar et al. 2021 [34]	PER	cross-sec	92.9%	high	1,773	993	56.0%	multiple median	---	---
Verbeeck et al. 2021 [35]	BEL	cohort	100.0%	high	4,390,750	---	---	---	---	---
Ward et al. 2021 [36]	USA-DC	cohort	100.0%	high	386,311	---	---	---	---	---
Zhang 2021 [37]	USA	cohort	100.0%	high	10,850	---	---	---	---	---

b)	Exposure									
	Reported occupations			Source of data acquisition			Period of data acquisition		Comparison group applied	
Al-Thani et al. 2021 [17]	diverse (*n*= 9)			interview			07/20-09/20		administrative workers	
Billingsley et al. 2022 [18]	diverse (*n*= 9)			Swedish occupational register			12/18		other	
Campbell et al. 2021 [19]	diverse (*n*= 4)			at work			01/21-03/21		other	
Cummings et al. 2021 [20]	diverse (*n*=23)			Employment Development Department (EDD) records			2020 (up to 6 months before death)		overall	
Della Valle et al. 2021 [38]	diverse (*n*= 3)			at work			05/20-07/20		office worker	
Dìaz-Salazar et al. 2021 [21]	diverse (*n*= 5)			register			07/20		office / management	
Finkenzeller et al. 2020 [22]	diverse (*n*=13)			at work			07/20		administration	
Fogh et al. 2021 [23]	diverse (*n*= 6)			web-based-questionnaire			09/20		office work	
Hawkins et al. 2021 [24]	diverse (*n*=11)			death certificate: “usual work”			03/20-07/20		office & administrative support	
House et al. 2021 [39]	meat packers			hospital documentation			03/20-05/20		all others	
IIAC 2021 [40]	diverse (*n*=88)			occupation recorded on the death certificate			2019		general population	
Lastrucci et al. 2020 [25]	diverse (*n*= 4)			questionnaire			05/20		work-from-home	
Magnusson et al. 2021 [26]	diverse (*n*=22)			register			08/20		individuals of working age	
Miller et al. 2021 [27]	farmworker (diverse)			at work			05/20-08/20		office work	
Möhner & Wolik 2020 [28]	diverse (*n*=19)			coded social security data			06/20-10/20		information and communication	
Möhner & Wolik 2021 [41]	diverse (*n*= 9)			coded social security data			01/20-05/20		all others	
Nafilyan et al. 2021 [29]	diverse (*n*=40)			Census data			2011		administrative occupations	
Nwaru et al. 2022 [30]	diverse (*n*= 8)			register			2018-2019		non-essential workers	
Poustchi et al. 2021 [31]	diverse (*n*= 6)			at work			04/20-06/20		general population	
Stringhini et al. 2021 [32]	diverse (*n*=16)			at work			05/20-09/20		public administration	
Stufano et al. 2021 [33]	correctional workers (diverse)			at work			11/20-01/21		administrative staff	
Tovar et al. 2021 [34]	diverse (*n*= 5)			at work			06/20-07/20		administration	
Verbeeck et al. 2021 [35]	diverse (*n*= 18)			database of active employees (NACE-BEL)			09/20-10/20		general population	
Ward et al. 2021 [36]	prison officers			register			(2015 -) 2019 – 2020		general population	
Zhang 2021 [37]	diverse (*n*=22)			Occupation recorded along with lab confirmed test			05/19		office and administrative support	

c)	Outcome									
	Specification / stage of severity			Proof					Period of data acquisition	
Al-Thani et al. 2021 [17]	infection			IgM/IgG, PCR					07/20-09/20	
Billingsley et al. 2022 [18]	mortality			ICD-10: U07.1, U07.2, B3.42					03/20-02/21	
Campbell et al. 2021 [19]	infection			PCR					01/21-03/21	
Cummings et al. 2021 [20]	mortality			death certificates: lab confirmed COVID-19 case					01/20-12/20	
Della Valle et al. 2021 [38]	infection			IgG, IgM					05/20-10/20	
Dìaz-Salazar et al. 2021 [21]	infection			IgM/IgG					07/20	
Finkenzeller et al. 2020 [22]	infection			IgM/IgG					07/20	
Fogh et al. 2021 [23]	infection			IgG, IgM					10/20	
Hawkins et al. 2021 [24]	mortality			death certificate ICD-10 U07.1 or free text					03/20-07/20	
House et al. 2021 [39]	infection, ICU			any test results					03/20-05/20	
IIAC 2021 [40]	mortality			death records ICD-10: U07.1 or U07.2					03/20-12/20	
Lastrucci et al. 2020 [25]	infection			IgM, IgG					05/20	
Magnusson et al. 2021 [26]	infection, hospitalization			PCR-testing; ICD-10: U07.1					02/20-12/20	
Miller et al. 2021 [27]	infection			NAAT-antigen test					05/20-08/20	
Möhner & Wolik 2020 [28]	hospitalization			ICD-10: U07.1, U07.2					06/20-10/20	
Möhner & Wolik 2021 [41]	hospitalization			ICD-10: U07.1, U07.2, U04.9, B34.2, B97.2					01/20-05/20	
Nafilyan et al. 2021 [29]	mortality			death certificate ICD-10 U07.1 or U07.2					01/20-12/20	
Nwaru et al. 2022 [30]	infection, hospitalization, ICU-admission			ICD-10: U07.1, U07.2					01/20-02/21	
Poustchi et al. 2021 [31]	infection			IgG, IgM					04/20-06/20	
Stringhini et al. 2021 [32]	infection			IgG					05/20-09/20	
Stufano et al. 2021 [33]	infection			rRT-PCR					11/20-12/20	
Tovar et al. 2021 [34]	infection			IgG, IgM					06/20-07/20	
Verbeeck et al. 2021 [35]	infection			register confirmed cases					09/20-10/20	
Ward et al. 2021 [36]	infection			PCR					03/20-11/20	
Zhang 2021 [37]	infection			lab confirmed					02/20-06/20	

Abbreviations: *SD* standard deviation, *IQR* interquartile range, *SSYK* Swedish standard for classification of occupation, *SOC 2010* Standard Occupational Classification 2010 (GB), *WZ 2008* Commercial sectors 2008 (Germany), *NACE-BEL* Statistical classification of economic activities in the European Community (Belgium), *ICU* Intensive care unit, *IgM / IgG* Immunoglobulin (antibodies), *PCR* polymerase-chain-reaction, *ICD-10* International Classification of Diseases, 10^th^ revision

### Characteristics of the studies

On search date, 21 of the publications included were peer-reviewed. Three publications retrieved from medRχiv were found published peer-reviewed at a tracking search nine months later (September 30th, 2022) [17, 19, 23]; one remaining publication accounts for grey literature [40].

The majority of the studies were conducted in Europe (*n* = 14), followed by the Americas (*n* = 9). While another two studies give an account of Asia, none was included representing Oceania or Africa. As individual countries, the USA (*n* = 6), Great Britain (*n* = 3), Norway (*n* = 3) and Germany (*n* = 3) stand out.

Studies cover the period from January 2020 to May 2021, with an emphasis (*n* = 21) on the year 2020. They were conducted either as cross-sectional studies (*n* = 9) or as cohorts (*n* = 16). The number of persons included ranged from 367 to 33,2 million.

As exposure, up to 88 occupations were reported in studies based on registers [40]. However, some studies focussed on one occupation only [36, 39].

Moreover, some studies referred to more than one outcome [26, 30, 39]. Primarily, infection (*n* = 18) was reported, while more severe outcomes, i.e., hospitalisation (*n* = 4), admission to intensive care unit (ICU) (*n* = 2) and mortality (*n* = 5), were reported less often.

### Risk of bias

Five publications were overall rated as low RoB (Table 2). However, in all five, ‘chronology’ – as a minor domain – was not considered as sufficient [18, 26, 29, 30, 40].

**Table 2:**
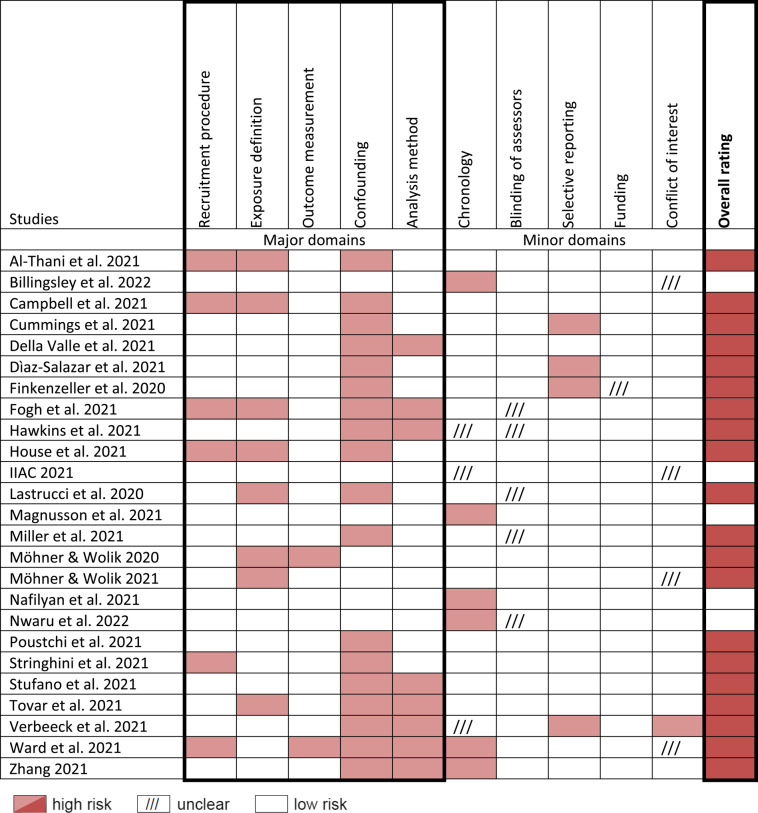
Risk of bias in 25 eligible texts [17–41]

All other publications were rated with a high RoB, mainly due to weaknesses in the evaluation of the data (confounding: *n* = 18; analysis method: *n* = 8) rather than at their collection (recruiting procedure: *n* = 6; exposure definition: *n* = 8; outcome measurement: *n* = 2). Thereby, most of the downgrades resulted from not considering age and sex as confounders.

### Data extraction and handling as well as its occupational classification

A total of 686 data sets were extracted, each describing an occupational risk of infection with SARS-CoV-2.

Overlapping studies were treated as described in the methods, so that data from IIAC - The Industrial Injuries Advisory Council [40] was used and Nafilyan et al. [29] was excluded. Within one study [35], two overlapping time periods occurred. Data quality assessment resulted in the exclusion of the data sets of one study [22]. As data of ICU admission emerged as a subgroup to data of infection [39] or to data of hospitalisation [30], in each case we preferred information of the larger group.

While some occupational descriptions in the studies were classified according to coding systems (e.g. in [18, 35, 37]), the majority of the descriptions used terms of everyday language. Some given descriptions combined two or three occupations and some descriptions were too vague as to be classifiable. Thus, the standardisation into the German classification system of KldB reduced the number of available data sets to 317.

As first choice comparators, ‘administrative occupations’ in private and public institutions (7130 and 7320, respectively) as well as ‘office clerks and secretaries’ (7140) were used. While ‘non-essential workers’ did not occur as a comparator, several studies referred their results to ‘other’, but the majority (67,2%) to the ‘general population’.

### Study results

**Fig. 2:**
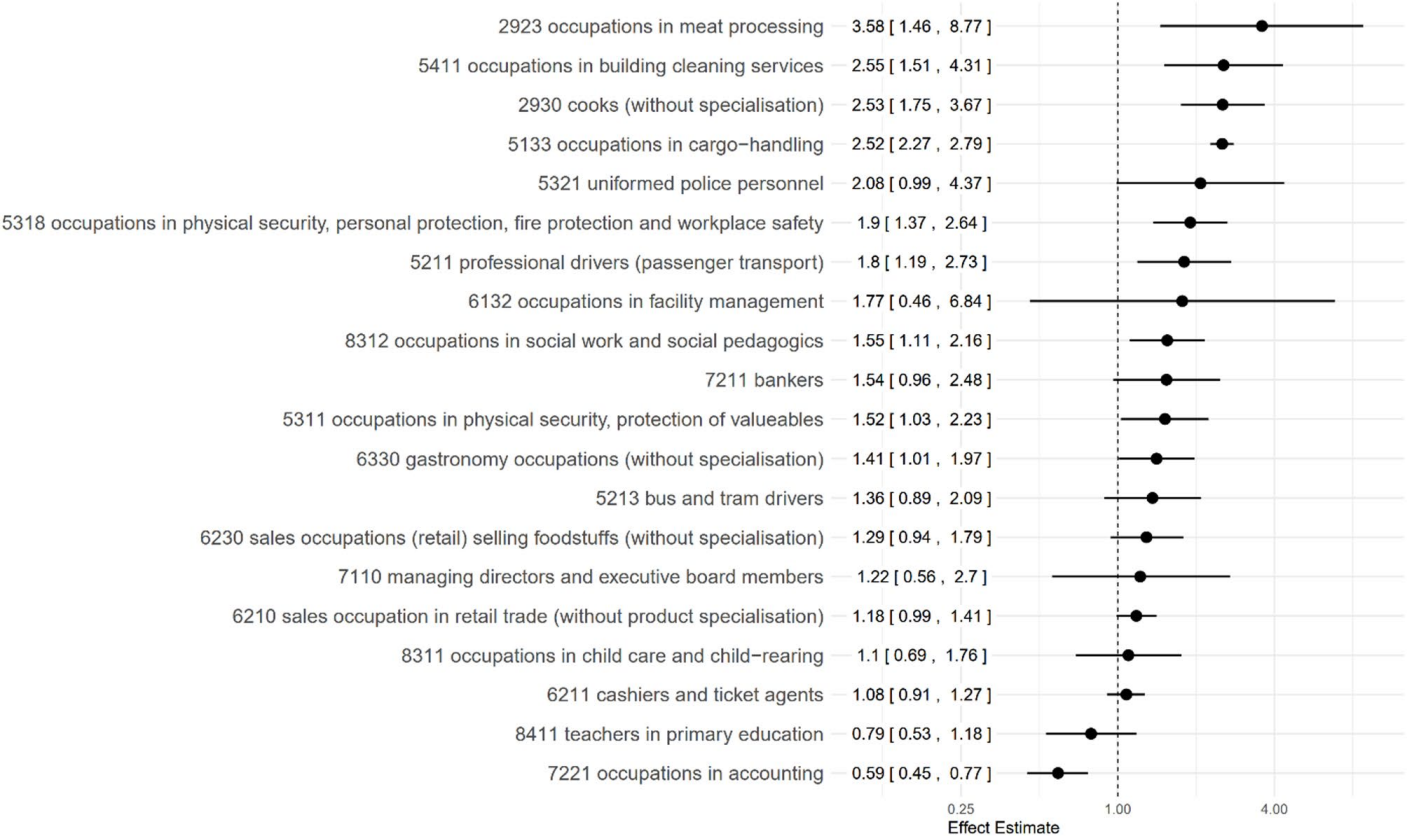
Results of meta-analyses at KldB-level-4 (dotted line at 1.0 marks the null result)

On KldB-level-4, a total of 170 data sets were available, representing 76 different occupations. For 20 of the occupations, three or more data sets from at least two different studies were available and, thus, a meta-analysis was performed (Fig. 2; Table 3; individual forest plots in Appendix A.4). These results are based on 93 data sets extracted from 17 studies.

**Table 3:** Results of meta-analyses and rating of evidence according to GRADE, initial level: moderate

KldB-4: occupational description								Downgrading					Upgrading		
		RR	95%-CI		Studies (N)	Data sets (n)	Evidence	1: Risk of Bias	2: Inconsistency	3: Indirectness	4: Precision	5: Publication-bias	6: Effect	7: Dose-Response	8: Underestimation
2923	occupations in meat-processig	3.58	1.46	8.77	4	4	moderate	0	0	0	-1	0	1	0	0
5411	occupations in building cleaning services	2.55	1.51	4.31	3	3	low	-1	0	0	-1	0	1	0	0
2930	cooks (without specialisation)	2.53	1.75	3.67	2	3	high	0	0	0	0	0	1	0	0
5133	occupations in cargo-handling	2.52	2.27	2.79	3	3	moderate	-1	0	0	0	0	1	0	0
5321	uniformed police personnel	2.08	0.99	4.37	4	4	low	-1	0	0	-2	0	1	0	0
5318	occupations in physical security, personal protection, fire protection and workplace safety (with specialisation, not elsewhere classified)	1.90	1.37	2.64	5	5	low	-1	0	0	0	0	0	0	0
5211	professional drivers (passengers transport)	1.80	1.19	2.73	4	5	moderate	0	0	0	0	0	0	0	0
6132	occupations in facility management	1.77	0.46	6.84	2	3	low	0	-1	0	-2	0	0	0	0
8312	occupations in social work and social pedagogics	1.55	1.11	2.16	4	4	moderate	0	0	0	0	0	0	0	0
7211	bankers	1.54	0.96	2.48	3	4	low	0	0	0	-2	0	0	0	0
5311	occupations in physical security, protection of valuables, and personal protection	1.52	1.03	2.23	2	4	moderate	0	0	0	0	0	0	0	0
6330	gastronomy occupations (without specialisation)	1.41	1.01	1.97	6	10	moderate	0	0	0	0	0	0	0	0
5213	bus and tram drivers	1.36	0.89	2.09	4	5	low	0	-1	0	-1	0	0	0	0
6230	sales occupations (retail) selling foodstuffs (without specialisation)	1.29	0.94	1.79	2	3	low	0	0	0	-1	0	0	0	0
7110	managing directors and executive board members	1.22	0.56	2.70	3	3	low	-1	0	0	-2	0	0	0	0
6210	sales occupations in retail trade (without product specialisation)	1.18	0.99	1.41	8	10	low	0	0	0	-1	0	0	0	0
8311	occupations in child care and child-rearing	1.10	0.69	1.76	6	10	low	0	0	0	-1	0	0	0	0
6211	cashiers and ticket agents	1.08	0.91	1.27	3	4	low	0	0	0	-1	0	0	0	0
8411	teachers in primary education	0.79	0.53	1.18	2	3	low	0	0	0	-1	0	0	0	0
7221	occupations in accounting	0.59	0.45	0.77	3	3	moderate	0	0	0	0	0	0	0	0

#### Meta-analyses

Nine occupations show a statistically significantly elevated risk of acquiring an infection with SARS-CoV-2.

- ‘Occupations in meat processing’ (2923) show the highest RR in all analyses (RR = 3.58 [1.46; 8.77]). Four data sets contribute to the result, with the information from low RoB (RR = 6.59 [3.97; 10.94]) exceeding this risk. They originate from studies conducted in Europe and North America, specifying all three stages of outcome.
- In ‘occupations in building cleaning services’ (5411), the RR = 2.55 [1.51; 4.31] results from three data sets. Their underlying cohort studies were all conducted in the US, specifying infection and mortality as outcome. They are all rated as high RoB.
- Three data sets contribute to the RR = 2.53 [1.75; 3.67] in ‘cooks’ (2930). Information from low RoB (RR = 3.11 [2.45; 3.93] exceeds the overall risk. Relying on information on infection as well as on mortality, the studies forming the basis were conducted in Europe and used administrative occupations and the general population as comparator.
- The RR = 2.52 [2.27; 2.79] of ‘occupations in cargo-handling’ (5133) is based on three data sets derived from US cohorts, describing infection as well as mortality. All studies were rated as high RoB.
- Four data sets contribute to the RR = 1.90 [1.37; 2.64] of ‘Occupations in physical security, personal protection, fire protection and workplace safety’ (5318). They originate cohort studies describing mortality as outcome. The information from low RoB (RR = 3.21 [2.71; 2.93]) exceeds the overall risk.
- ‘Professional drivers in passenger transport’ (5211) show a RR = 1.80 [1.19; 2.73]. It results from five data sets derived from European studies and incorporating all three stages of outcome (infection, hospitalization and mortality).
- For ‘occupations in social work and social pedagogics’ (8312), four data sets were combined resulting in a RR = 1.55 [1.11; 2.16]. They originate from studies conducted in Europe and the US considering infection and mortality as outcome.
- Reporting infection and mortality as outcome, the four data sets describing ‘occupations in physical security, protection of valuables, and personal protection’ (5311) result in RR = 1.52 [1.03; 2.23]. The studies were conducted in Great Britain and in Qatar.
- A total of ten data sets contribute to ‘gastronomy occupations (not specialized)’ (6330) (RR = 1.41 [1.01; 1.97]). The underlying cohort studies were conducted in Europe and the US, reporting infection as well as mortality as outcome.

Half of the reported occupations show a result not statistically significant, whereof the risk estimator of nine is elevated and one is decreased.

- The four data sets contributing to the result of ‘uniformed police personnel’ (5321) (RR = 2.08 [0.99; 4.37]) are derived from studies conducted in Europe, describing infection and mortality as outcome
- ‘Occupations in facility management’ (6132) are based on three data sets resulting in RR = 1.77 [0.46; 6.84]. The studies were conducted in Great Britain and in Mexico reporting infection as well as mortality as outcome
- For ‘bankers’ (7211), four data sets were combined, resulting in RR = 1.54 [0.96; 2.48]. They originate from studies conducted in Europe and in Iran considering infection and mortality as outcome
- The RR = 1.36 [0.89; 2.09] for ‘bus and tram drivers’ (5213) is based on five data sets reporting infection and mortality as outcome. They originate from Europe and South America. The information from low RoB not only exceeds the overall result (RR = 1.82 [1.23; 2.68]) but opposes that from high RoB in effect direction (RR = 0.87 [0.65; 1.15]).
- ‘Sales occupations (retail) selling foodstuffs’ (6230) show a RR = 1.29 [0.94; 1.79] with high and low RoB being equal. The three data sets report infection only and originate from European studies
- The three data sets contributing to ‘managing directors and executive board members’ (7110) result in RR = 1.22 [0.56; 2.70]. The studies from Switzerland and the US are all rated as high RoB and only use ‘administrative occupations’ as comparator.
- ‘Sales occupations in retail trade’ (6210) comprise ten data sets showing a RR = 1.18 [0.99; 1.41]. Results are reported from Europe, the Americas and Asia, and all three outcomes are considered (infection, hospitalization and mortality). There is no difference between high and low RoB results.
- Four data sets contribute to the RR = 1.08 [0.91; 1.27] in ‘cashiers and ticket agents’ (6211). They originate from studies carried out in Europe and Iran reporting infection and mortality as outcome.
- To describe ‘occupations in child care and child rearing’ (8311), a total of ten data sets are available (RR = 1.10 [0.69; 1.76]). The underlying studies were all conducted in European countries and report all three stages of outcome (infection, hospitalization and mortality).
- The RR = 0.79 [0.53; 1.18] of ‘teachers in primary education’ (8411) combines infection and mortality as outcome, but refers to ‘general population’ only. Three data sets derived from cohort studies conducted in England and Wales as well as in Norway form the result’s basis. All studies were rated as low RoB.

As a unique result, ‘occupations in accounting’ (7221) show a statistically significantly decreased infection risk (RR = 0.59 [0.45; 0.77]. Three data sets from cohort studies conducted in the US as well as in England and Wales contribute to it. As outcome, infection as well as mortality were examined.

In half of the results (*n* = 10) I^2^ was higher than 90%, in seven between 50% and 89%, and only in three occupations I^2^ was below 50%.

#### Subgroup analyses

Subgroup analyses for ‘outcome’ (Appendix A.5.1) showed a higher risk for ‘mortality’ than for ‘infection’ for the majority of occupations, with exceptions in ‘occupations in building cleaning services’ (5411), in ‘managing directors and executive board members’ (7110) and in ‘teachers in primary education’ (8411). In ‘sales occupations (retail) selling foodstuffs’ (6230), no differences were observed. A relation for ‘hospitalisation’ could not be determined due to lack of data.

In subgroup analyses for ‘comparison group’ (Appendix A.5.2), the majority of the results with ‘administrative occupations’ as denominator were lower than those with ‘general population’. Results using ‘other’ as denominator were indifferent in this context.

Subgroup analyses for ‘risk of bias’ (Appendix A.5.3) indicated a ‘bias towards the null’ for results derived from high RoB studies: The majority of results of studies with low bias potential showed a more pronounced result than the overall risk. Exceptions were found in ‘professional drivers’ in passenger transport (5211) and in ‘occupations in child care and child rearing’ (8311). No differences were observed in the results of ‘sales occupations (retail) selling foodstuffs’ (6230).

#### Grading of evidence

Starting our assessment at ‘moderate quality of evidence’, we graded the results’ evidence from high to low (see Table 3).

About two thirds of the results were downgraded for ‘precision’. While ‘risk of bias’ induced a downgrading in five cases, this happened twice because of ‘inconsistency’. Upgrading was possible for five results by ‘effect size’. The categories ‘indirectness’ and ‘publication bias’ for downgrading and ‘dose-response’ and ‘under-estimation’ for upgrading were not applied.

In ‘cooks’ (2930), there was no downgrading but an upgrading because of its RR > 2 (‘effect size’). Hence, we conclude high evidence that we can rely on the meta-analysis’ result.

The result of five occupations (5211; 5311; 6330; 7221; 8312) neither received a downgrade nor an upgrade. In two occupations, an initial downgrading was nullified by an upgrading due to ‘effect size’: While in ‘occupations in cargo handling’ (5133) downgrading was based on ‘risk of bias’, in ‘occupations in meat processing’ (2923) it was based on ‘precision’. Thus, for seven results, we stay with the moderate evidence we gave initially.

Despite an upgrading because of their effect size, ‘occupations in building cleaning services’ (5411) as well as in ‘uniformed police personnel’ (5321) were downgraded for ‘precision’ and ‘risk of bias’. In six results, a sole downgrading was applied because of ‘precision’ (7211; 6230; 6210; 6211; 8311; 8411) and another one because of ‘risk of bias’ (5318). In three occupations (6132; 5213; 7110), the downgrading had more than one reason. Thereby, for twelve results, we conclude low evidence that we can rely on the meta-analyses’ result.

## Discussion

We conducted a systematic review and meta-analysis to examine the risk of SARS-CoV-2 infection in various nonhealthcare occupations. A total of 25 publications were included, mainly describing the first year of the COVID-19 pandemic. Meta-analyses were performed for 20 occupations other than HCWs, predominantly showing an increased risk of exposure to SARS-CoV-2.

For seven occupations we identified a statistically significantly increased risk of being infected with SARS-CoV-2, which we classified as moderate to high evidence. All the occupations identified in our study as belonging to the ‘food and hospitality’ sector (2923; 2930; 6330) and to the ‘social services’ sector (8312) constitute this category, complemented by individual occupations in the ‘transport and logistics’ sector (5211; 5133) and the ‘security and surveillance’ sector (5311). Two further occupations, attributable to ‘security and surveillance’ again (5318) as well as to ‘cleaning’ (5411), showed a risk for infection significantly increased, however, they were rated as low evidence. These occupations cover three of four sectors specified as employing ‘essential workers’, which were defined by Castellazzi [42] as ‘agriculture’, ‘health and personal care’, ‘transportation’ and ‘food distribution’. In Germany, ‘law enforcement agencies’ are considered essential in addition to ‘food’, ‘transportation’ and ‘emergency and rescue services’ [4]. The majority requires personal contact in their execution.

For another ten occupations, we identified a risk not statistically significant and rated with low evidence, predominantly showing an elevated risk. Though the vast majority can be considered as ‘essential workers’ as well, the application of protective measures was possible: In some occupations, such as ‘bus and tram drivers’ (5213) as well as ‘sales occupations (retail) selling foodstuffs’ (6230) and ‘cashiers and ticket agents’ (6211), the potential for high infection rates was anticipated due to high numbers of customers. As preventive measure acrylic glass shields were installed as a technical barrier. In certain time frames, the reduction of maximum number of customers in grocery shops further enabled the mandatory regulation of distancing while in public transport the overall number of passengers was reduced due to lockdown. A high risk was also expected in occupations in contact with children, whereupon several countries included ‘occupations of child care and child rearing’ (8311) as well as ‘teachers in primary education’ (8411) in the organisational measures for closure (cf. [5]). However, the closure of nurseries and schools might have brought their occupational risk into line with that of other homeworking occupations.

For a single occupation, we identified a statistically significantly decreased risk of being infected with SARS-CoV-2, rating it with moderate evidence: Even though ‘accounting’ can be attributed to ‘financial services’, which is listed as an ‘essential occupation’ [4], it can be performed digitally, avoiding any personal contact.

As only observational studies were included in the systematic review and subsequent meta-analyses, the results represent risks that were influenced by both individual behaviour and policy-related factors. Thereby, our results do not represent a raw risk, but should be read in the context of policy measures applied. In addition, different periods of policy measures in the countries contributing to meta-analyses may further explain ambiguous findings.

Occupations described in our study predominantly account for the tertiary sector, with ‘food-processing’ being the only occupational group representing the secondary sector; occupations of the primary sector do not appear. Though ‘agriculture’ [42] or ‘farming, fishing and forestry’ [5] are termed consistently as ‘essential’ or ‘frontline’ occupations, we did not find enough datasets in the eligible literature to perform a meta-analysis.

Meta-analyses were calculated predominantly for occupations of lower social status. A reason for this focus can be found in aspects of data protection, as very small numbers in cases led to non-reporting in the original studies [40] and applies in occupations with low numbers of representatives, including leadership positions. This is in contrast to results based on the German National Cohort [43], where a remarkable number of occupational sectors of higher social status were reported as having a higher infection risk in the first wave.

The heterogeneity perceived as ‘considerable’ (I^2^: 75-100%) to ‘substantial’ (I^2^: 50-90%) might be explained by the consideration of different outcomes, the dynamic rate of new infection and the different implementation of prevention measures in the countries under consideration.

Four out of eight domains of GRADE did not result in a modification of the rating. As all studies considered working adults, ‘indirectness’ did not need to be addressed. Due to the pandemic event in which every article on COVID-19 was published, predominantly with ‘open access’ [44, 45], no ‘publication bias’ was observed. The binary outcome, i.e., virus detected or not, detained from assessing a possible ‘dose-response’ relationship. Finally, ‘under-estimation’ was not observed.

The definition specified a priori for downgrading in the domain ‘risk of bias’ seems too strict to us in retrospect. As it refers to a difference between results of low RoB and high RoB, it does not take into account whether results of low RoB tend towards a strong or weak relationship. Thus, we might have downgraded a result, despite its subgroup result of low RoB exceeded the overall result.

The methodological quality of the included studies varied considerably.

In terms of ‘exposure’, the accuracy of the occupational description ranged from self-declaration to information provided by the employer according to a classification system. The former was sometimes easier to translate into a standardised form than the latter, because translation tools offered more than one suggestion. Thereby, the differentiation appeared to be very simplistic, because different activities – and therefore exposures – could not be taken into account. Although we have taken into account that information on current employment may be out of date if the data collection was more than 12 months before infection, there may have been other misclassifications, e.g., inaccuracies in death certificates.

In terms of ‘outcome’, we considered three stages of rising severity. While for the infection itself the availability of testing capacity was a crucial factor leading to a potential bias in the results [40], mortality is influenced by many additional factors such as pre-existing illnesses.

Though ‘age’ and ‘sex’ were known as confounders in an early stage of pandemia [46], most of our eligible literature did not take them into account. This lack, however, is not only a disadvantage in observational studies but was detected in clinical studies as well [47]. Comparably, information on socio-economic status (SES) was not recorded in all our included studies, for which reason a confounding effect on our results cannot be precluded. Furthermore, studies published within our search period did not address comorbidities. A recently published case-cohort study using German statutory health insurance data did include comorbidities in its analyses [48]. However, their inclusion did not explicitly change the conclusion but generally supported our findings.

The strength of sex as an influencing factor depends on the stage of the outcome. While infection itself showed no sex differences, it was found pronounced in case fatality rate [46]. As its marked focus was identified in the age groups 50–59 [46] and 60–69 [49], this is related to the working population. Thus, results derived by mortality data will probably overestimate the occupational risk in professions predominantly executed by elderly male when no adjustments for sex were applied (cf. [40]).

In times of pandemia, it is hard to find an unexposed group considerable as comparator. Hence, we used a hierarchically structured approach of ‘little exposed’ or ‘less exposed than’. Of the four possible categories defined in the PROSPERO protocol, three were covered in the extracted literature. In the majority of the literature, a non-exposed comparison group was used (e.g. [50]). However, in some cases, the group assumed as most exposed (HCWs) was regarded as comparator (e.g. [51]). Taking an exposed group as a comparator, results in underestimating a risk.

Furthermore, the subgroup analysis regarding ‘risk of bias’ revealed a ‘bias towards the null’ for studies rated as high RoB. Again, this results in underestimating a risk.

Of the 25 studies included, 20 were classified as studies with a high RoB. This was mainly due to weaknesses in the evaluation of the data, such as insufficient consideration of confounding factors and inappropriate analytical methods. In addition, there were weaknesses in the recruitment of participants and the definition of exposure. Only five of the included studies showed a low RoB, while at least one minor bias factor was present, as well. As a rare situation, we had to exclude one of these studies [29], as it overlapped substantially in area and period with another study rated as low RoB [40].

We were able to extract 76 occupations from the literature at KldB-level 4. However, only for a restricted number of 20 we can report results, while two-thirds of the occupations were available in only one or two data sets. This can be attributed to the underlying studies’ own methodological aspects as some focussed on ‘essential occupations’ explicitly [18] or took into account the expected level of exposure [38], both resulting in a focus on service occupations. Other studies followed statistical considerations and excluded occupations with low numbers of employees or low numbers of infections [26, 35, 40], which leads to a limitation to occupations with higher risk of infection. Outbreak studies do not contribute to our result.

Though some studies defined their occupational exposure according to classification systems [18, 35, 37, 40], the majority did not. Thereby, we were not able to transfer all our data to the international acknowledged classification of the International Classification of Occupations (ISCO). The latter follows a skill level with level of leadership as first grouping, which was not indicated in the extracted data sets. In contrast, the German classification starts with the sector and narrows it down to educational aspects on level 5 (which we did not consider) [10, 52]. This enabled us to assign each data set according to its potential depth.

While a detailed occupational description was desirable, it has the disadvantage of scaling down the number of cases. Furthermore, similar occupational tasks – such as passenger transportation in busses and in taxis – are separated though they might pose a comparable risk.

Finding more than 9.000 texts within a search frame of two years are a massive output for a topic as young as COVID-19, even more as occupational risk is often a side issue. We refrained from performing forwards or backwards citation tracking [53] due to a very limited time interval (i.e., 01.01.2020–01.02.2022). Instead, to include all upcoming publications, we searched medRχiv for relevant texts, a database that rapidly gained attention during the pandemic [54]. As all texts retrieved from this source were peer reviewed by the time of data extraction, the confidence in their quality was given [55].

The majority of the texts excluded in title/abstract screening focussed on HCWs. This emphasis has been assessed in the literature before [40]. Not only their risk of infection was a subject matter but also their status of mental health [56–59].

For all texts eligible, the pdf was retrieved without problem for full-text screening. This probably is due to an understanding of cooperation [44, 45], issued by the scientific community at the beginning of the pandemic, to make research results available free of charge.

Though we conducted our search at the end of the second year of the pandemic, the reported data of the included studies pool in the first year. As vaccination only occurred in the end of 2020 and by then was offered exclusively to selected groups not being of interest in this study (i.e., HCWs, elders, and vulnerable persons), we covered a period in which the effect of vaccination is neglectable.

While studies giving insights on pandemic impact in African countries were rare but existed [60–62], there were none from Oceania. This probably can be explained by their isolated position and their rigorous border controls, especially within the first year. Thereby, spread within occupations was scarce and not worth doing research on.

We examined the risk of SARS-SoV-2 infection in occupations outside of the healthcare sector systematically. Search for literature was conducted worldwide and in all languages. Studies not available in German or English were translated professionally for full text screening (i.e., Spanish, Italian, Persian). Thereby, we are able to present a full picture of the scientific literature referring to the first year of COVID-19 pandemia, enabling us to describe the risk of infection without the effect of vaccination.

We describe the risk of infection for a broad variety of occupations contextualising service occupations. They cover a huge number of workers and were assumed to have a high risk of infection because of their professional activity.

We are not able to give information on occupations in which few people were employed, such as rare professions or leading positions. Furthermore, self-employed occupations are underrepresented.

Within an occupation, different tasks and activities are performed, of which some pose a higher risk for infection than others. The varying risk of infection rather depends on whether these tasks are performed or not than on being employed in a certain job. However, as information as detailed as that was not assessed in times of the pandemic, occupation is a suitable proxy.

In analysing the first year of pandemia, we integrated at least two waves. Studies considering them separately showed marked differences in occupational risk [63, 64].

In COVID-19 pandemia, the workplace became a field of public health. Protective measures implemented by health policy ranged from personal protective equipment to extensive lockdown, and had an impact on workplace safety and infection rates. It differed between industries and countries, and essential workers were protected from SARS-CoV-2 infection to a varying degree.

As home-office has gained popularity since then, health policy measures of the pandemic have long-term effects in the workplace. The experiences made in the pandemic should be further analysed in order to be able to make recommendations for action in the future.

## Conclusion

This study is one of the first to systematically examine the risk of SARS-CoV-2 infection in occupations outside the healthcare sector. In occupations of individual related services as well as in commercial services, a considerable elevated risk for SARS-CoV-2 infection was found.

## Supplementary Information

Supplementary Material 1.

Supplementary Material 2.

## Data Availability

No datasets were generated or analysed during the current study.

## Abbreviations

GRADE: Grading of Recommendations Assessments, Development, and Evaluation
HCWs: Healthcare workers
ICU: Intensive care unit
IIAC: Industrial Injuries Advisory Council
ISCO: International Standard Classification of Occupations
KldB: German classification of professions (“Klassifikation der Berufe”)
PECOS: Population, Exposure, Comparison, Outcome, Study type
PRISMA: Preferred Reporting Items for Systematic reviews and Meta-Analyses
PROSPERO: International prospective register of systematic reviews
RoB: Risk of bias
RR: Relative risk
SES: Socio-economic status
