# Supplementary material for: SARS-CoV-2 infection risk by non-healthcare occupations: a systematic review and meta-analysis
Source: J Occup Med Toxicol. 2025 May 22;20:17. doi: 10.1186/s12995-025-00462-9 (PMC12096541; doi:10.1186/s12995-025-00462-9)
Supplement: Supplementary file 1 — Supplementary Material 1. [file 12995_2025_462_MOESM1_ESM.pdf]

A. Appendix 2
A.1 Search strings 2
A.2 justification for full texts' exclusion and excluded texts by reasons 4
A.3 GRADE 11
A.4 Individual results' graphs 12
A.5 subgroup analyses 32

Appendix A

A.1 Search strings

A.1.1 PubMed

Tab. A.1-1: applied search strings in Medline via PubMed on February 1st, 2022

Exposure	("occupational diseases"[MeSH Terms] OR "occupational exposure"[MeSH Terms] OR "occupational medicine"[MeSH Terms] OR "occupational risk"[Text Word] OR "occupational hazard"[Text Word] OR "occupational group*"[Text Word] OR ("industry"[MeSH Terms] AND "mortality"[MeSH Subheading]) OR "work-related"[All Fields] OR "air pollutants, occupational"[MeSH Terms] OR "working environment"[Text Word] OR "occupational health"[MeSH Terms] OR "workplace"[MeSH Terms] OR "profession*"[Text Word] OR "job"[Text Word] OR "occupation*"[Text Word] OR "work*"[Text Word]) AND "employ*"[Text Word]
Outcome	("Coronavirus"[MeSH Terms] OR "Coronavirus"[All Fields] OR "coronaviruses"[All Fields] OR "corona virus"[All Fields] OR ("Coronaviridae"[MeSH Terms] OR "Coronaviridae"[All Fields] OR "coronavirinae"[All Fields]) OR ("Coronaviridae"[MeSH Terms] OR "Coronaviridae"[All Fields]) OR ("Betacoronavirus"[MeSH Terms] OR "Betacoronavirus"[All Fields] OR "betacoronaviruses"[All Fields]) OR ("COVID-19"[MeSH Terms] OR "COVID-19"[All Fields] OR "covid19"[All Fields]) OR "COVID-19"[All Fields] OR ("SARS-CoV-2"[MeSH Terms] OR "SARS-CoV-2"[All Fields] OR "ncov"[All Fields]) OR "CoV 2"[All Fields] OR "CoV2"[All Fields] OR "sarscov2"[All Fields] OR "2019nCoV"[All Fields] OR "novel CoV"[All Fields] OR "wuhan virus"[All Fields] OR (("wuhan"[All Fields] OR "hubei"[All Fields] OR "huanan"[All Fields]) AND ("severe acute respiratory"[All Fields] OR ("pneumonia"[MeSH Terms] OR "pneumonia"[All Fields] OR "pneumonias"[All Fields] OR "pneumoniae"[All Fields] OR "pneumoniae s"[All Fields])) AND ("disease outbreaks"[MeSH Terms] OR ("disease"[All Fields] AND "outbreaks"[All Fields]) OR "disease outbreaks"[All Fields] OR "outbreak"[All Fields] OR "epidemiology"[MeSH Subheading] OR "epidemiology"[All Fields] OR "outbreaks"[All Fields] OR "outbreak s"[All Fields])) OR "Coronavirus"[MeSH Terms] OR "Coronavirus Infections"[MeSH Terms] OR "COVID-19"[Supplementary Concept] OR "severe acute respiratory syndrome coronavirus 2"[Supplementary Concept] OR "Betacoronavirus"[MeSH Terms])
Limitation	(2020/1/1:3000/12/12[pdat])

A.1.2 EMBASE

Tab. A.1-2: applied search strings in EMBASE on February 1st, 2022.

Exposure	('occupational groups' OR 'worker'/exp/mj OR 'occupational hazard'/exp/mj<br>OR 'occupational danger' OR 'occupational hazard' OR 'occupational risk'<br>OR 'workplace'/exp/mj OR 'work'/exp/mj OR 'occupational disease'/exp/mj<br>OR 'occupational medicine' OR 'occupational accident'/exp/mj<br>OR 'work environment'/exp/mj OR 'occupational safety')<br>AND
Outcome	('severe acute respiratory syndrome coronavirus 2'/exp/mj<br>OR 'coronavirus disease 2019'/exp/mj OR 'coronavirus infection'<br>OR 'coronaviridae infection' OR 'coronavirinae' OR 'corona virus'<br>OR 'betacoronavirus' OR 'beta coronavirus' OR 'wuhan virus' OR 'covid19'<br>OR 'coronavirus disease 2019' OR '2019 novel coronavirus disease'<br>OR '2019 novel coronavirus epidemic' OR '2019 novel coronavirus infection'<br>OR '2019-ncov disease' OR '2019-ncov infection' OR 'covid' OR 'covid 19'<br>OR 'covid 19 induced pneumonia' OR 'covid-10' OR 'covid-19'<br>OR 'covid-19 induced pneumonia' OR 'covid-19 pneumonia'<br>OR 'sars coronavirus 2 infection' OR 'sars coronavirus 2 pneumonia'<br>OR 'sars-cov-2 disease' OR 'sars-cov-2 infection' OR 'sars-cov-2 pneumonia'<br>OR 'sars-cov2 disease' OR 'sars-cov2 infection' OR 'sarscov2 disease'<br>OR 'sarscov2 infection' OR 'wuhan coronavirus disease'<br>OR 'wuhan coronavirus infection' OR 'coronavirus disease 2'<br>OR 'coronavirus disease 2010' OR 'coronavirus disease 2019'<br>OR 'coronavirus disease 2019 pneumonia' OR 'coronavirus disease-19'<br>OR 'coronavirus infection 2019' OR 'ncov 2019 disease' OR 'ncov 2019 infection' OR 'new coronavirus pneumonia' OR 'novel coronavirus 2019 disease'<br>OR 'novel coronavirus 2019 infection' OR 'novel coronavirus disease 2019'<br>OR 'novel coronavirus infected pneumonia'<br>OR 'novel coronavirus infection 2019' OR 'novel coronavirus pneumonia'<br>OR 'paucisymptomatic coronavirus disease 2019'<br>OR 'severe acute respiratory syndrome 2'<br>OR 'severe acute respiratory syndrome 2 pneumonia'<br>OR 'severe acute respiratory syndrome cov-2 infection'<br>OR 'severe acute respiratory syndrome coronavirus 2 infection'<br>OR 'severe acute respiratory syndrome coronavirus 2019 infection' OR 'covid 19' OR ncov OR 'cov 2' OR cov2 OR sarscov2 OR 2019ncov<br>OR '2019 novel coronavirus' OR 'severe acute respiratory or pneumonia'<br>OR coronavirus OR 'coronavir*' OR 'betacoronavir*'<br>OR '2019 novel coronavirus*')<br>AND
Limitation	[2020-2022]/py

A.1.3 medRχiv

Tab. A.1-3: applied search in medRχiv on February 2nd, 2022.

Outcome AND exposure	Abstract or Title: COVID-19 AND employee<br>Abstract or Title: COVID-19 AND employment<br>Abstract or Title: COVID-19 AND job<br>Abstract or Title: COVID-19 AND occupation<br>Abstract or Title: COVID-19 AND occupational exposure<br>Abstract or Title: COVID-19 AND worker<br>Abstract or Title: COVID-19 AND workplace<br><br>Abstract or Title: SARS-CoV-2 AND employee<br>Abstract or Title: SARS-CoV-2 AND employment<br>Abstract or Title: SARS-CoV-2 AND job<br>Abstract or Title: SARS-CoV-2 AND occupation<br>Abstract or Title: SARS-CoV-2 AND occupational exposure<br>Abstract or Title: SARS-CoV-2 AND worker<br>Abstract or Title: SARS-CoV-2 AND workplace
Limitation	date posted: 01.01.2020-02.02.2022

A.2 Justification for full texts' exclusion and excluded texts by reasons

Tab. A.2-1: categories for excluding full texts.

population	non-working or unemployed population, age below 16
exposition	studies include only workers in health service facilities (i.e., health care workers and other employees in inpatient and outpatient care and in welfare)
occupation	no occupational groups given
comparison	without comparison group
outcome	other infectious diseases only; long COVID only
infection	no number for infected persons given
study design	RCTs, qualitative studies, ecological studies, case reports, (quasi-) experimental studies, comments, letters, editorials, congress abstracts, posters, reviews
response	a response lower than 10 % of the selected population; no information on response given; convenience sample
else	multiple testing; modelling results; no results reported for occupations in studies with other aim of analysis;

A.2.1 population

No exclusions due to this reason.

A.2.2 exposition

authors	ye	title
Goetz, Delaughder et al.	2020	Asymptomatic Employee Screening for SARS-CoV-2: Implementation of and Reactions to an Employer-Based Testing Program
Majiya, Aliyu-Paiko et al.	2021	Seroprevalence of SARS-CoV-2 in Niger State: A Pilot Cross Sectional Study
Ward, Atchison et al.	2020	Antibody prevalence for SARS-CoV-2 in England following first peak of the pandemic: REACT2 study in 100,000 adults
Warszawski, Meyer et al.	2021	Trends in social exposure to SARS-Cov-2 in France. Evidence from the national socio-epidemiological cohort - EPICOV

A.2.3 occupation

authors	ye	title
Aatresh, Cummings et al.	2021	Development of at-home sample collection logistics for large scale SARS-CoV-2 seroprevalence studies
Al Kuwari, Abdul Rahim et al.	2020	Characterization of the SARS-CoV-2 outbreak in the State of Qatar, February 28-April 18, 2020
Anand, Allen et al.	2022	Work-related and personal predictors of COVID-19 transmission: evidence from the UK and USA
Anna, Goyard et al.	2020	High seroprevalence but short-lived immune response to SARS-CoV-2 infection in Paris
Aung, Vittinghoff et al.	2021	Characteristics and Behaviors Associated with Prevalent SARS-CoV-2 Infection
Ayoubkhani, Nafilyan et al.	2020	Ethnic minority groups in England and Wales - factors affecting the size and timing of elevated COVID-19 mortality: a retrospective cohort study linking Census and death records
Bajunirwe et al.	2021	Long-distance truck drivers and the increasing risk of COVID-19 spread in Uganda
Barrett, Horton et al.	2020	Prevalence of SARS-CoV-2 infection in previously undiagnosed health care workers at the onset of the U.S. COVID-19 epidemic
Bartko, Zehetmayer et al.	2021	Screening and Confirmatory Testing for SARS-CoV-2 Antibodies: Comparison of Health and Non-Health Workers in a Nationwide Healthcare Organization in Central Europe
Batty, Deary et al.	2020	Psychosocial factors and hospitalisations for COVID-19: Prospective cohort study of the general population
Bontadi, Bergamo et al.	2020	Efficacia delle misure volte a contenere la diffusione del virus Sars-Cov-2 negli ambienti di lavoro: un'indagine in nove aziende con sede nella regione Veneto
Chamie, Marquez et al.	2020	SARS-CoV-2 Community Transmission During Shelter-in-Place in San Francisco
Costa, Meireles et al.	2021	Incidence of SARS-CoV-2 infection in a cohort of workers from the University of Porto
Davis, Carr et al.	2021	Indicators of past COVID-19 infection status: Findings from a large occupational cohort of staff and postgraduate research students from a UK university
Davis, Oetzmann et al.	2021	Unexplained longitudinal variability in COVID-19 antibody status by Lateral Flow Immuno-Antibody testing
de la Rosa Ruiz and Guillén Astete	2021	[COVID-19: modification of the risk of infection and disease development associated with the performance of blended work.]
De Negri, Galiezz et al.	2021	Socioeconomic factors and the probability of death by Covid-19 in Brazil
Elliott, Bodinier et al.	2021	COVID-19 mortality in the UK Biobank cohort: revisiting and evaluating risk factors
Fatima, Bucks et al.	2021	Shift work is associated with increased risk of COVID-19: Findings from the UK Biobank cohort
Garbarino, Domnich et al.	2021	Seroprevalence of SARS-CoV-2 in a Large Cohort of Italian Police Officers
Haag, Blankenburg et al.	2021	Prevalence and Transmission of SARS-CoV-2 in Childcare Facilities: A Longitudinal Study
Hassink, Kalb et al.	2021	Regional Coronavirus Hotspots During the COVID-19 Outbreak in the Netherlands
Holt, Talaei et al.	2021	Risk factors for developing COVID-19: a population-based longitudinal study (COVIDENCE UK)
Jantzen, Noisel et al.	2020	Epidemiological and socio-economic characteristics of the COVID-19 spring outbreak in Quebec, Canada: A population-based study
Jeremijenko, Chemaitelly et al.	2020	Evidence for and level of herd immunity against SARS-CoV-2 infection: the ten-community study
Jerković, Ljubić et al.	2021	SARS-CoV-2 Antibody Seroprevalence in Industry Workers in Split-Dalmatia and Šibenik-Knin County, Croatia
Kadyrova, Yegorov et al.	2021	Seroprevalence of anti-SARS-CoV-2 IgG and IgA antibodies before the launch of COVID-19 vaccination in Kazakhstan
Kaspersen, Greve et al.	2021	Symptoms reported by SARS-CoV-2 seropositive and seronegative healthcare and administrative employees in Denmark from May to August 2020
Labberton, Godoy et al.	2021	SARS-CoV-2 infections and hospitalizations among immigrants in Norway: significance of occupation, household crowding, education, household income and medical risk. A nationwide register study
Laursen, Petersen et al.	2021	Prevalence of SARS-CoV-2 IgG/IgM Antibodies among Danish and Swedish Falck Emergency and Non-Emergency Healthcare Workers

Lee, Han et al.	2021	Analysis of Superspreading Potential from Transmission Clusters of COVID-19 in South Korea
Leidi, Berner et al.	2021	Occupational risk of SARS-CoV-2 infection and reinfection during the second pandemic surge: a cohort study
Limaye, Ninesling et al.	2021	COVID-19 Pandemic Response in a Migrant Farmworker Community: Excess Mortality, Testing Access and Contact Tracing in Immokalee, Florida
Liu, Wu et al.	2020	Prevalence of IgG antibodies to SARS-CoV-2 in Wuhan -implications for the ability to produce long-lasting protective antibodies against SARS-CoV-2
Maidstone, Anderson et al.	2021	Shift work is associated with positive COVID-19 status in hospitalised patients
Marshall, Bois et al.	2020	Sentinel Coronavirus Environmental Monitoring Can Contribute to Detecting Asymptomatic SARS-CoV-2 Virus Spreaders and Can Verify Effectiveness of Workplace COVID-19 Controls
Matthay, Duchowny et al.	2021	Contributions of occupation characteristics and educational attainment to racial/ethnic inequities in COVID-19 mortality
Mema, Frosst et al.	2021	COVID-19 outbreak among temporary foreign workers in British Columbia, March to May 2020
Nishikimi, Watanabe et al.	2022	Prevalence of SARS-CoV-2 antibodies after one-year follow up among workers in a research institute in Japan
Pathela, Crawley et al.	2021	Seroprevalence of Severe Acute Respiratory Syndrome Coronavirus 2 Following the Largest Initial Epidemic Wave in the United States: Findings From New York City, 13 May to 21 July 2020
Riley, Ainslie et al.	2020	Transient dynamics of SARS-CoV-2 as England exited national lockdown
Riley, Ainslie et al.	2020	Resurgence of SARS-CoV-2 in England: detection by community antigen surveillance
Riley, Eales et al.	2021	REACT-1 round 8 final report: high average prevalence with regional heterogeneity of trends in SARS-CoV-2 infection in the community in England during January 2021
Riley, Walters et al.	2021	REACT-1 round 9 interim report: downward trend of SARS-CoV-2 in England inFebruary 2021 but still at high prevalence
Riley, Walters et al.	2021	REACT-1 round 9 final report: Continued but slowing decline of prevalence of SARS-CoV-2 during national lockdown in England in February 2021
Rowlands, Gillies et al.	2021	Association of working shifts, inside and outside of healthcare, with severe COVID-19: an observational study
Sarailoo et al.	2021	Investigating the relationship between occupation and SARS-CoV2
Shahani, Farouq et al.	2021	Survey of COVID-19 associated symptoms and reported deaths in an urban community in Kano, Nigeria
Takita, Matsumura et al.	2020	Preliminary Results of Seroprevalence of SARS-CoV-2 at Community Clinics in Tokyo
Talaei, Faustini et al.	2022	Determinants of pre-vaccination antibody responses to SARS-CoV-2: a population-based longitudinal study (COVIDENCE UK)
Tejamaya, Phanprsit et al.	2022	Characteristics of COVID-19 infection clusters occurring among workers in several Asia-Pacific countries
Thielecke, Theuring et al.	2020	SARS-CoV-2 infections in kindergartens and associated households at the start of the second wave in Berlin, Germany - a cross sectional study
Tomasi, Ramirez-Cardenas et al.	2021	COVID-19 mortality among Amalgamated Transit Union (ATU) and Transport Workers Union (TWU) workers-March-July 2020, New York City metro area
Tuček	2021	COVID-19 in the Czech Republic 2020: probable transmission of the coronavirus SARS-CoV-2
Wu, Xie et al.	2021	Epidemiological and clinical characteristics of severe acute respiratory coronavirus virus 2
Zhang, Saade et al.	2021	SARS-CoV-2 Seroprevalence among First Responders in Northeastern Ohio

A.2.4 comparison

authors	ye	title
Backhaus, Hermsen et al.	2021	Underascertainment of COVID-19 cases among first responders: a seroepidemiological study
De Rooij, Sikkema et al.	2021	Potential environmental transmission routes of SARS-CoV-2 inside a large meat processing plant experiencing COVID-19 clusters
Di Leone, Drago et al.	2020	[Integrated management method in the prevention department of a COVID-19 epidemic outbreak in a large meat processing plant in Bari province]
Dyal, Grant et al.	2020	COVID-19 Among Workers in Meat and Poultry Processing Facilities - 19 States, April 2020
Esposito, Neglia et al.	2021	Epidemiology of SARS-CoV-2 Infection Evaluated by Immunochromatographic Rapid Testing for the Determination of IgM and IgG Against SARS-CoV-2 in a Cohort of Mask Wearing Workers in the Metal-Mechanical Sector in an Area With a High Incidence of COVID-19
Finch, Lowe et al.	2021	SARS-CoV-2 infection and reinfection in a seroepidemiological workplace cohort in the United States

Gjestvang Moe et al.	2021	SARS-CoV-2 serological findings and exposure risk among employees in school and retail after first and second wave COVID-19 pandemic in Oslo, Norway: a cohort study
Goldfarb et al.	2021	SARS-CoV-2 seroprevalence among Vancouver public school staff in British Columbia, Canada
Günther, Czech-Sioli et al.	2020	SARS-CoV-2 outbreak investigation in a German meat processing plant
Herstein, Degarege et al.	2021	Characteristics of SARS-CoV-2 transmission among meat processing workers in Nebraska, USA, and effectiveness of risk mitigation measures
Hibino, Hayashida et al.	2021	COVID-19 seropositivity changes in asymptomatic individuals during the second and third waves of COVID-19 in Tokyo
Kalia, Moraga et al.	2021	Use of Vinegar and Water to Identify COVID-19 Cases During a Workplace Entrance Screening Protocol
Kim	2020	Social Distancing and Public Health Guidelines at Workplaces in Korea: Responses to Coronavirus Disease-19
Kojima, Roshani et al.	2021	Incidence of Severe Acute Respiratory Syndrome Coronavirus-2 infection among previously infected or vaccinated employees
Krygier, Stępień et al.	2021	Rapid serological tests for SARS-CoV-2 IgG/IgM - not worth attention?
Lan, Suharlim et al.	2021	Association between SARS-CoV-2 infection, exposure risk and mental health among a cohort of essential retail workers in the USA
Lan, Wei et al.	2020	Work-related COVID-19 transmission in six Asian countries/areas: A follow-up study
Lewis, Dack et al.	2021	Was the risk of death among the population of teachers and other school workers in England and Wales due to COVID-19 and all causes higher than other occupations during the pandemic in 2020? An ecological study using routinely collected data on deaths from the Office for National Statistics
Lopez, Weber et al.	2020	Seroprevalence of anti-SARS-CoV-2 IgG Antibodies in the Staff of a Public School System in the Midwestern United States
Malagón-Rojas, Parra et al.	2020	Infection and risk perception of sars-cov-2 among airport workers: A mixed methods study
Mallet, Pivette et al.	2021	Identification of Workers at Increased Risk of Infection During a COVID-19 Outbreak in a Meat Processing Plant, France, May 2020
Martins-Filho, Da Silva et al.	2021	Seroprevalence of SARS-CoV-2 antibodies in broadcast media workers
Meireles, Amaro et al.	2021	Prevalence of SARS-CoV-2 antibodies among workers of the public higher education institutions of Porto, Portugal: A cross-sectional study
Milleliri, Coulibaly et al.	2021	SARS-CoV-2 in Ivory Coast: serosurveillance survey among mines workers
Ming, Han et al.	2020	Environmental monitoring shows SARS-CoV-2 contamination of surfaces in food plants
Mioch, Kuiper et al.	2021	SARS-CoV-2 antibodies in employees working in non-medical contact-intensive professions in the Netherlands: Baseline data from the prospective COco-study
Modenese, Mazzoli et al.	2021	Frequency of Anti-SARS-CoV-2 Antibodies in Various Occupational Sectors in an Industrialized Area of Northern Italy from May to October 2020
Nygren, Norén et al.	2021	Association between SARS-CoV-2 and exposure risks in health care workers and university employees–a cross-sectional study
Rosero, Mas et al.	2021	COVID-19 Prevalence and prevention behaviors among US certified organic producers
Sendi, Baldan et al.	2021	A multidimensional cross-sectional analysis of COVID-19 seroprevalence among a police officer cohort: The PoliCOV-19 study
Steinberg, Kennedy et al.	2020	COVID-19 Outbreak Among Employees at a Meat Processing Facility - South Dakota, March-April 2020
Strathdee, Abramovitz et al.	2021	Prevalence and correlates of SARS-CoV-2 seropositivity among people who inject drugs in the San Diego-Tijuana border region
Thomas, Matthay et al.	2022	A descriptive analysis of 2020 California Occupational Safety and Health Administration covid-19-related complaints
Thompson, Burgess et al.	2021	Prevention and Attenuation of COVID-19 by BNT162b2 and mRNA-1273 Vaccines
Vieira, Tang et al.	2021	SARS-CoV-2 antibody seroprevalence among firefighters in Orange County, California
Walshe, Fennelly et al.	2021	Assessment of Environmental and Occupational Risk Factors for the Mitigation and Containment of a COVID-19 Outbreak in a Meat Processing Plant
Waltenburg, Rose et al.	2021	Coronavirus disease among workers in food processing, food manufacturing, and agriculture workplaces
Waltenburg, Victoroff et al.	2020	Update: COVID-19 Among Workers in Meat and Poultry Processing Facilities - United States, April-May 2020

A.2.5 outcome

No exclusions due to this reason.

A.2.6 infection

authors	ye	title
Cox-Ganser and Henneberger	2021	Occupations by Proximity and Indoor/Outdoor Work: Relevance to COVID-19 in All Workers and Black/Hispanic Workers
Crowell, Hanson et al.	2021	Union Efforts to Reduce COVID-19 Infections Among Grocery Store Workers
De Rooij, Hakze-Van Der Honing et al.	2021	Occupational and environmental exposure to SARS-CoV-2 in and around infected mink farms
Forster, Streng et al.	2022	Feasibility of SARS-CoV-2 Surveillance Testing among Children and Childcare Workers at German Day Care Centers: A Nonrandomized Controlled Trial
Ljubic, Banovac et al.	2020	The effect of serological screening for SARS-CoV-2 antibodies to participants' attitudes and risk behaviour: a study on a tested population sample of industry workers in Split-Dalmatia County, Croatia
Lotfollahzadeh, Rastgoo et al.	2021	Investigating the compliance of COVID-19 protocols in the workplaces of Ardabil, Iran
Marinaccio, Boccuni et al.	2020	Occupational factors in the COVID-19 pandemic in Italy: Compensation claims applications support establishing an occupational surveillance system
Nelson, Fosdick et al.	2021	Low prevalence of COVID-19 Exposure is Coincident with Self-reported Compliance with Public Health Guidelines among Essential Employees at an Institute of Higher Education
Nelson, Fosdick et al.	2021	Association Between COVID-19 Exposure and Self-reported Compliance With Public Health Guidelines Among Essential Employees at an Institution of Higher Education in the US
Paskarini, Haqi et al.	2020	Analysis of risk of exposure to COVID-19 in fishermen in Kenjeran

A.2.7 study design

authors	ye	title
Albin, Anders et al.	2021	Risk of inpatient care with COVID-19 in different occupational groups: The role of close human contacts in the work environment
Al-Naemi, Hassanen et al.	2021	Life span of Covid-19 antibodies following infection in a sample worker population in Qatar
Baysson, Pennachio et al.	2021	The Specchio-COVID19 cohort study: a longitudinal follow-up of SARS-CoV-2 serosurvey participants in the canton of Geneva, Switzerland (Study protocol)
Caban-Martinez, Gaglani et al.	2021	High Burden of COVID-19 among Unvaccinated Law Enforcement Officers and Firefighters
Chen, Aldridge et al.	2021	COVID-19 outbreak rates and infection attack rates associated with the workplace: a descriptive epidemiological study
De and Price	2021	Pre-existing Economic Conditions and COVID-19 Infections and Mortality in New York City
De Cassia Dos Santos Nery, Jouti et al.	2021	COVID-19 in essential activities: Report on successful measures in occupational health for 7,000 workers in an environmental sanitation company in Brazil
Feehan, Velasco et al.	2021	Racial and workplace disparities in seroprevalence of SARS-CoV-2, Baton Rouge, Louisiana, USA
Kern, Kuhlmann et al.	2022	SARS-CoV-2 surveillance (09/2020 - 03/2021) in elementary schools and daycare facilities in Bavaria
Lugg-Widger, Cannings-John et al.	2020	Establishing the impact of COVID-19 on the health outcomes of domiciliary care workers in Wales using routine data: a protocol for the OSCAR study
Methi, Telle et al.	2021	COVID-19 among bartenders and waiters before and after pub lockdown
Moret, Staley et al.	2021	Is COVID-19 an occupational disease?
Moura-Corrêa, Campos et al.	2021	Exposure to SARS-COV-2 at work and coronavirus disease (COVID-19): Survey with workers
Naicker, Jeebhay et al.	2021	Development and challenges of a national occupational health surveillance system for COVID-19 in the workplace
Naidoo and Jeebhay	2021	COVID-19: A new burden of respiratory disease among South African miners?
Pearce et al.	2021	Occupational differences in COVID-19 incidence, severity, and mortality in the United Kingdom: Available data and framework for analyses
Pokora, Kutschbach et al.	2021	Investigation of superspreading COVID-19 outbreak events in meat and poultry processing plants in Germany: A cross-sectional study
Rao, Ma et al.	2021	A disproportionate epidemic: COVID-19 cases and deaths among essential workers in Toronto, Canada
Straif, Espinosa et al.	2021	Occupational risk factors for SARS-COV-2 infection and COVID-19: Results from the covicat cohort study in Catalonia, Spain
Van Tongeren, Pearce et al.	2021	Occupation and COVID-19 mortality in england: A national linked data study of 14.3 million adults

A.2.8 response

authors	ye	title
Abu-Raddad, Chemaitelly et al.	2020	Characterizing the Qatar advanced-phase SARS-CoV-2 epidemic
Airoldi, Calcagno et al.	2021	Seroprevalence of SARS-CoV-2 Among Workers in Northern Italy
Beale, Hoskins et al.	2021	Differential Risk of SARS-CoV-2 Infection by Occupation: Evidence from the Virus Watch prospective cohort study in England and Wales
Beale, Patel et al.	2021	Occupation, Work-Related Contact, and SARS-CoV-2 Anti-Nucleocapsid Serological Status: Findings from the Virus Watch prospective cohort study
Beltrán, Basombrío et al.	2021	[Knowledge, attitudes, and practices about COVID-19 in Argentina. A cross-sectional study]
Berselli, Filippini et al.	2021	Seroprevalence of anti-SARS-CoV-2 antibodies in the Northern Italy population before the COVID-19 second wave
Cabas et al.	2021	Community pharmacists' exposure to COVID-19
Chadeau-Hyam, Eales et al.	2021	REACT-1 round 15 interim report: High and rising prevalence of SARS-CoV-2 infection in England from end of September 2021 followed by a fall in late October 2021
Chen, Glymour et al.	2021	Excess mortality associated with the COVID-19 pandemic among Californians 18-65 years of age, by occupational sector and occupation: March through November 2020
Colmenares-Mejía, Serrano-Díaz et al.	2021	Seroprevalence of sars-cov-2 infection among occupational groups from the bucaramanga metropolitan area, Colombia
Contreras, Ngo et al.	2021	Industry Sectors Highly Affected by Worksite Outbreaks of Coronavirus Disease, Los Angeles County, California, USA, March 19-September 30, 2020
Eriksen, Fogh et al.	2021	SARS-CoV-2 antibody prevalence among homeless people, sex workers and shelter workers in Denmark: a nationwide cross-sectional study
Fenton, Gribben et al.	2021	Risk of hospitalisation with COVID-19 among teachers compared to healthcare workers and other working-age adults. A nationwide case-control study
Green and Semple	2021	Occupational inequalities in the prevalence of COVID-19: A longitudinal observational study of England, August 2020 to January 2021
Hiironen, Saavedra-Campos et al.	2020	Occupational exposures associated with being a COVID-19 case. evidence from three case-control studies
Hua, Yin et al.	2022	Elevated COVID-19 Case Rates of Government Employees, District of Columbia, 2020-22
Islamoglu, Cengiz et al.	2021	COVID-19 seroconversion in the aircrew from Turkey
Kabagambe, Velasco-Gonzalez et al.	2022	Prevalence, Distribution and IgG Antibody Levels Associated with Severe Acute Respiratory Syndrome Coronavirus 2 (SARS-CoV-2) Among Health-System and Community-Based Employees and Patients
Kindzierski, Van Loon et al.	2021	SARS-CoV-2 screening prevalence in educational staff in Berlin, Germany, June-December 2020
Kotsiou, Pantazopoulos et al.	2021	Repeated Antigen-Based Rapid Diagnostic Testing for Estimating the Coronavirus Disease 2019 Prevalence from the Perspective of the Workers' Vulnerability before and during the Lockdown
Larsen, Fonager et al.	2021	Preliminary report of an outbreak of SARS-CoV-2 in mink and mink farmers associated with community spread, Denmark, June to November 2020
Leightley, Vitiello et al.	2021	The King's College London Coronavirus Health and Experiences of Colleagues at King's Study: SARS-CoV-2 antibody response in an occupational sample
Lerdsamran, Mungaomklang et al.	2022	Seroprevalence of anti-SARS coronavirus 2 antibodies in Thai adults during the first three epidemic waves
Lewnard, Mora et al.	2021	Prevalence and clinical profile of SARS-CoV-2 infection among farmworkers in Monterey County, California: June-November, 2020
Ling, Yu et al.	2020	Seroprevalence and epidemiological characteristics of immunoglobulin M and G antibodies against SARS-CoV-2 in asymptomatic people in Wuhan, China
Liu, Yang et al.	2021	Occupational characteristics in the outbreak of the covid-19 delta variant in nanjing, china: Rethinking the occupational health and safety vulnerability of essential workers
Ly, Nguyen et al.	2021	Screening of SARS-CoV-2 among homeless people, asylum-seekers and other people living in precarious conditions in Marseille, France, March-April 2020
Moe, Eriksen et al.	2021	SARS-CoV-2 serological findings and exposure risk among employees in school and retail after first and second wave COVID-19 pandemic in Oslo, Norway: a cohort study
Montague, Wipperman et al.	2021	Anti-SARS-CoV-2 IgA Identifies Asymptomatic Infection in First Responders
Mora, Lewnard et al.	2021	Risk factors for SARS-CoV-2 infection among farmworkers in Monterey County, California
Moradi, Mohamadi-Bolbanabad et al.	2021	Seroepidemiology of COVID-19 in high-risk occupational groups in west of Iran, November 2020
Mulligan, Berg et al.	2021	SARS-CoV-2 Seroprevalence Among Firefighters in Los Angeles, California
Murti, Achonu et al.	2021	COVID-19 Workplace Outbreaks by Industry Sector and Their Associated Household Transmission, Ontario, Canada, January to June, 2020
Mutambudzi, Niedzwiedz et al.	2021	Occupation and risk of severe COVID-19: Prospective cohort study of 120 075 UK Biobank participants

Pettifor, Diprete et al.	2021	A prospective study of asymptomatic SARS-CoV-2 infection among individuals involved in academic research under limited operations during the COVID-19 pandemic
Quashie, Mutungi et al.	2021	Trends of SARS-CoV-2 antibody prevalence in selected regions across Ghana
Rao, Robinson et al.	2021	Occupational exposures and mitigation strategies among homeless shelter workers at risk of COVID-19
Rowland, Bogulski et al.	2021	Experiences of Marshallese Food Processing Workers during the COVID-19 Pandemic
Satpati, Sarangi et al.	2020	Sero-surveillance (IgG) of SARS-CoV-2 among Asymptomatic General population of Paschim Medinipur District, West Bengal, India(Conducted during last week of July and 1st week of August 2020) - A Joint Venture of VRDL Lab (ICMR), Midnapore Medical College & Hospital & Department of Health and Family Welfare,Govt. of West Bengal, Paschim Medinipur
Thomas, Fina et al.	2021	Social, demographic and behavioural determinants of SARS-CoV-2 infection: A case-control study carried out during mass community testing of asymptomatic individuals in South Wales, December 2020
Toniato, Avolio et al.	2021	Evaluation of IgG seroprevalence of the Sars-Cov-2 in different worker categories
Zaidi, Rizwan et al.	2021	Seroprevalence of anti-SARS-CoV-2 antibodies in residents of Karachi-challenges in acquiring herd immunity for COVID 19
Zell, Wisnewski et al.	2021	Associations of SARS-CoV-2 serum IgG with occupation and demographics of military personnel

A.2.9 else

authors	ye	title
Al-Kuwari, Al-Nuaimi et al.	2021	COVID-19 infection across workplace settings in Qatar: a comparison of COVID-19 positivity rates of screened workers from March 1st until July 31st, 2020
Allan-Blitz et al.	2021	Characteristics of SARS-CoV-2 Positive Individuals in California From Two Periods During Notable Decline in Incident Infection
Allan-Blitz, Hertlein et al.	2021	Heterogeneity in SARS-CoV-2 Positivity by Ethnicity in Los Angeles
Brune, Korth et al.	2021	[SARS-CoV-2 IgG seroprevalence in personnel of the extraclinical fight against the COVID-19 pandemic]
Buchan, Smith et al.	2021	Incidence of outbreak-associated COVID-19 cases by industry in Ontario, Canada, April 1, 2020-March 31, 2021
de Gier et al.	2021	Occupation- and age-associated risk of SARS-CoV-2 test positivity, the Netherlands, June to October 2020
Kjøllesdal and Magnusson	2021	Occupational risk of COVID-19 by country of birth. A register-based study
Pasco, Fox et al.	2020	Estimated Association of Construction Work with Risks of COVID-19 Infection and Hospitalization in Texas

A.3 GRADE

	rating	description	comment
Downgrades			
Risk of bias	-1	In subgroup analysis, the result of high risk studies differs from those of low risk studies	
	-1	Only high risk studies form the basis of the result	
Inconsistency	-1		
Indirectness	-1		Did not apply in this review
Imprecision	-1	CI includes 1	Hazard identification;<br>Risk assessment
	-1	RR CI-range > 2	Risk assessment
	-2	CI includes 1 and RR CI-range > 2	
Publication bias	-1		Did not apply in this review

Upgrades			
Effect size	+1	2 ≤ RR < 5	
	+1	0,2 < RR ≤ 0,5	
	+2	RR ≥ 5	
	+2	RR ≤ 0,2	
Dose-response	+1		Did not apply in this review
Under-estimation	+1		Did not occur in this review

A.4 Individual results' graphs

A.4.1 2923 – occupations in meat processing

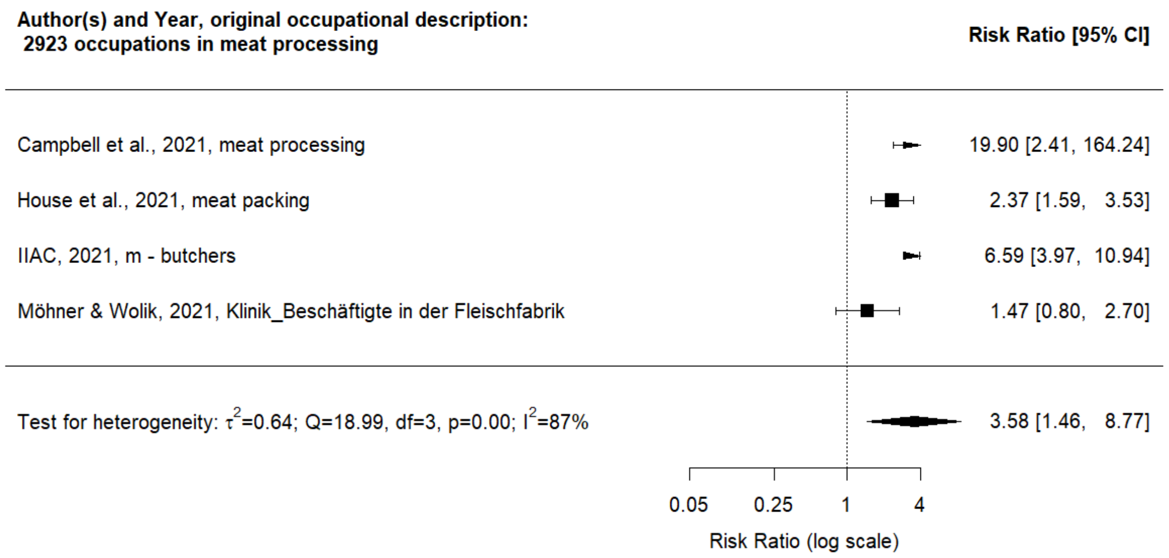

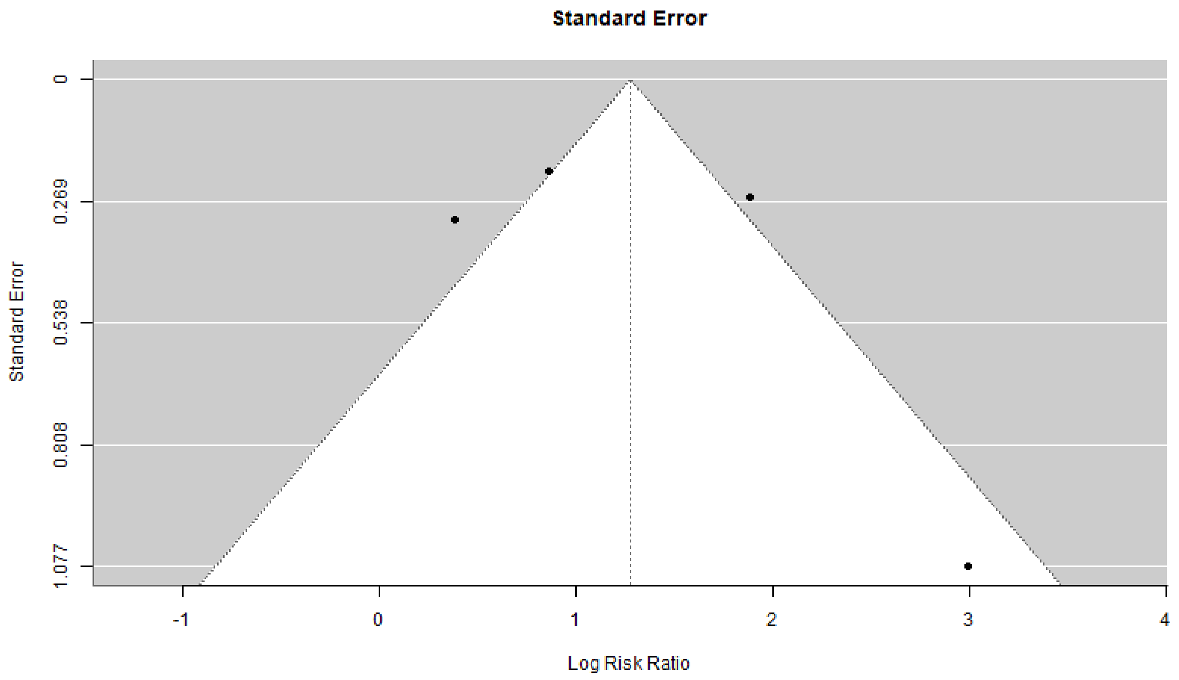

A.4.2 2930 – cooks (without specification)

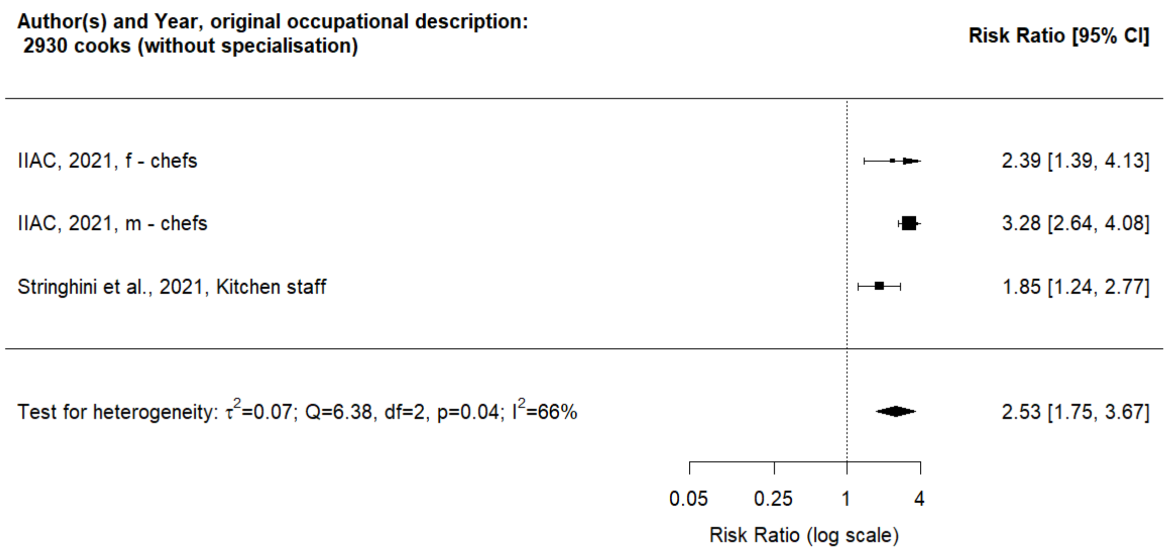

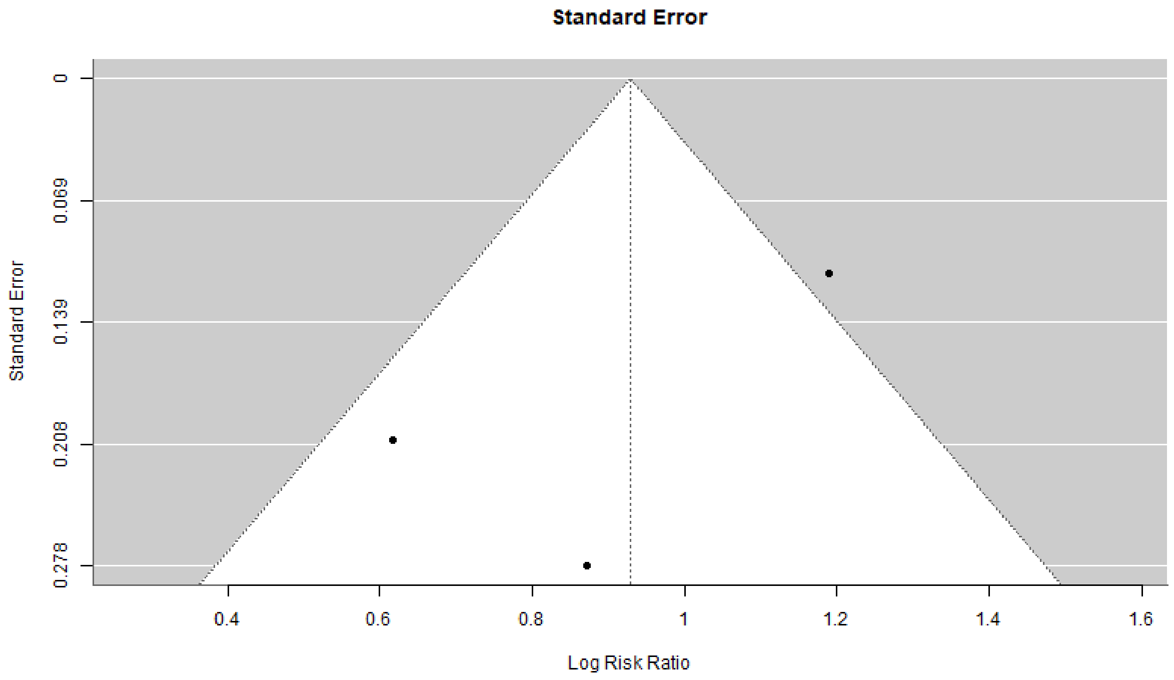

A.4.3 5133 – occupations in cargo-handling

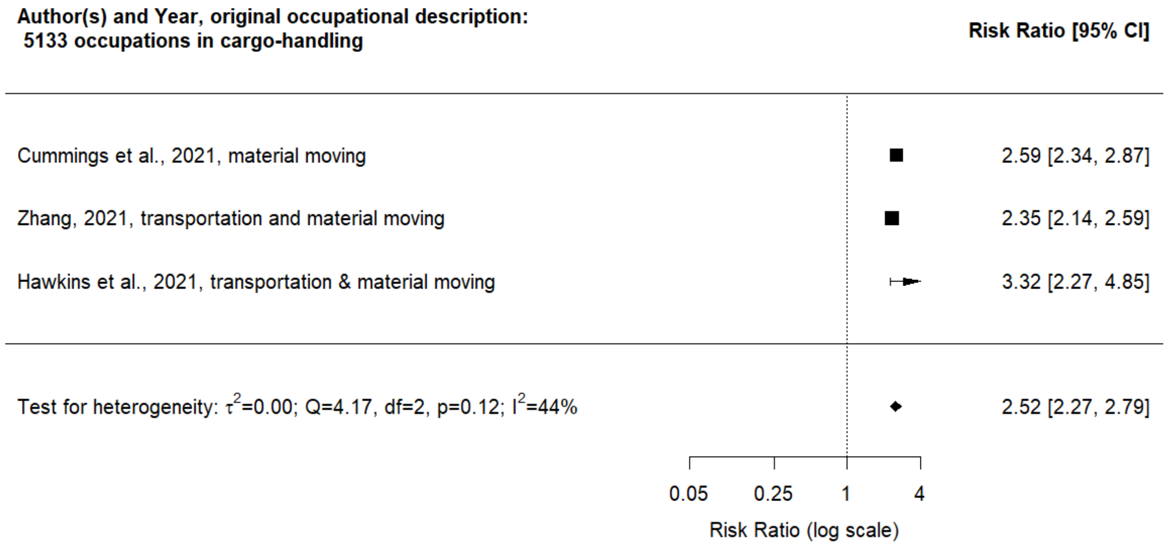

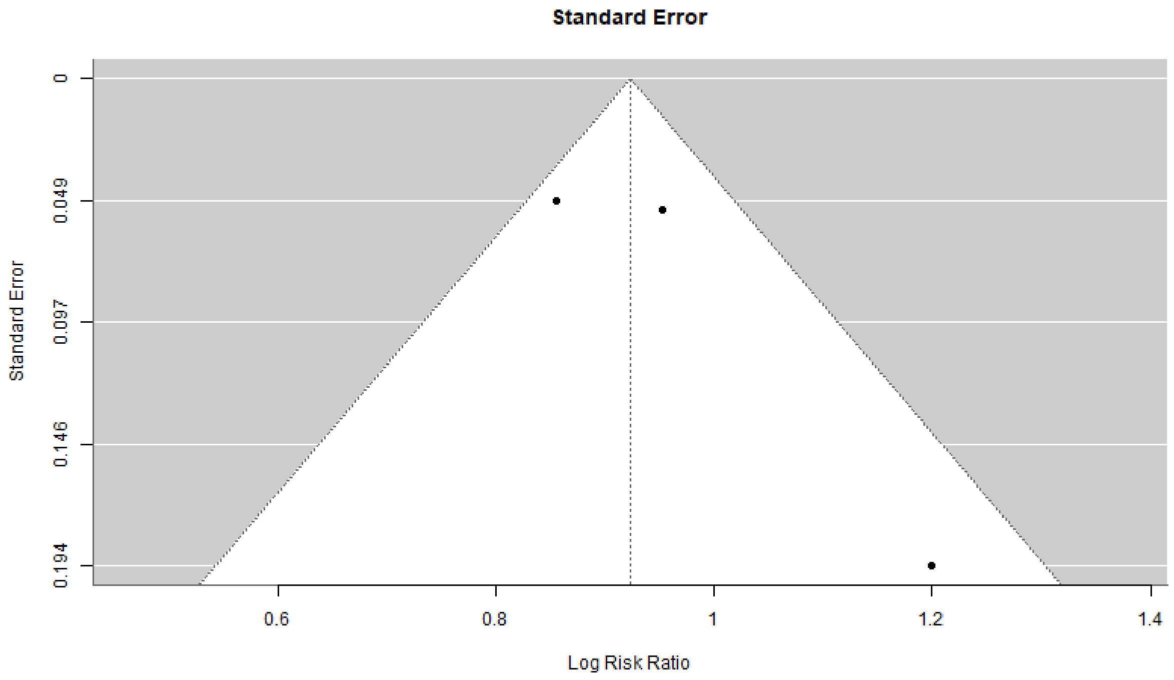

A.4.4 5211 – professional drivers (passenger transport)

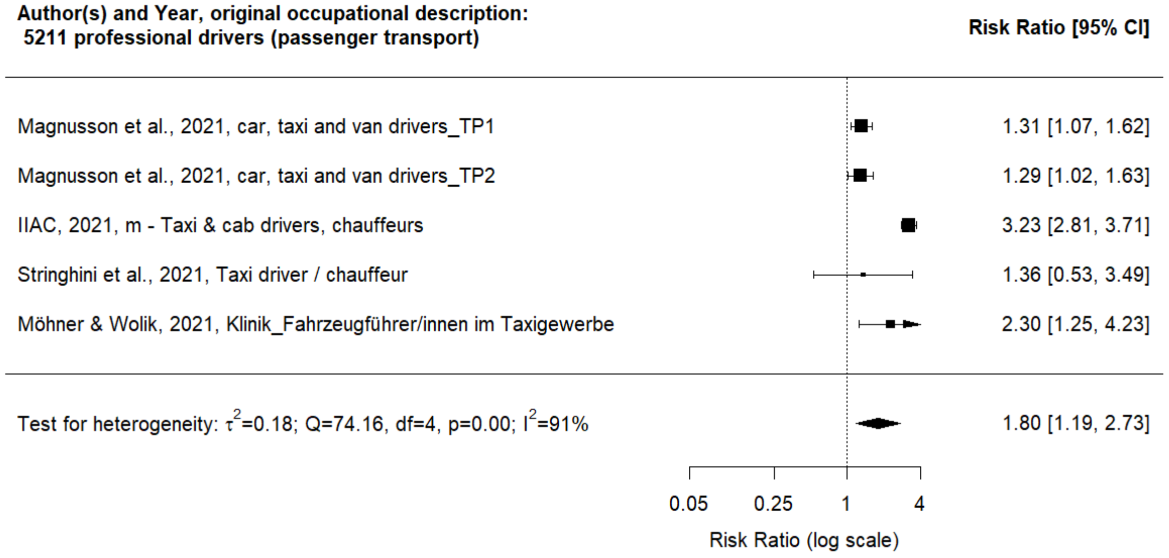

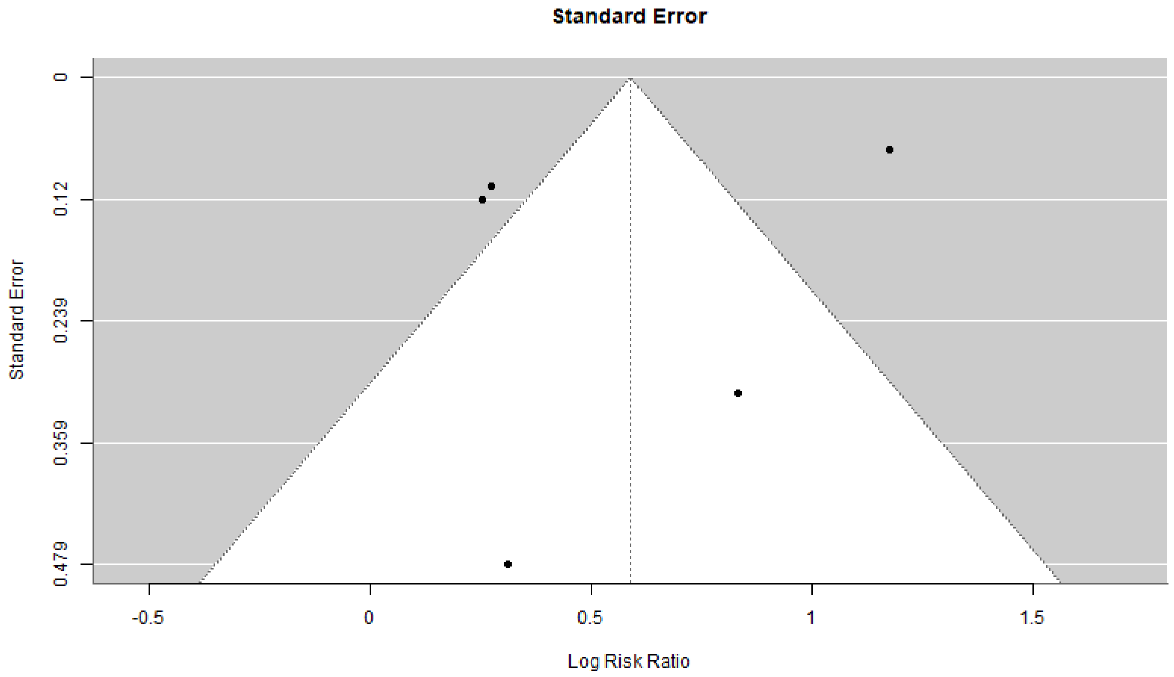

A.4.5 5213 – bus and tram drivers

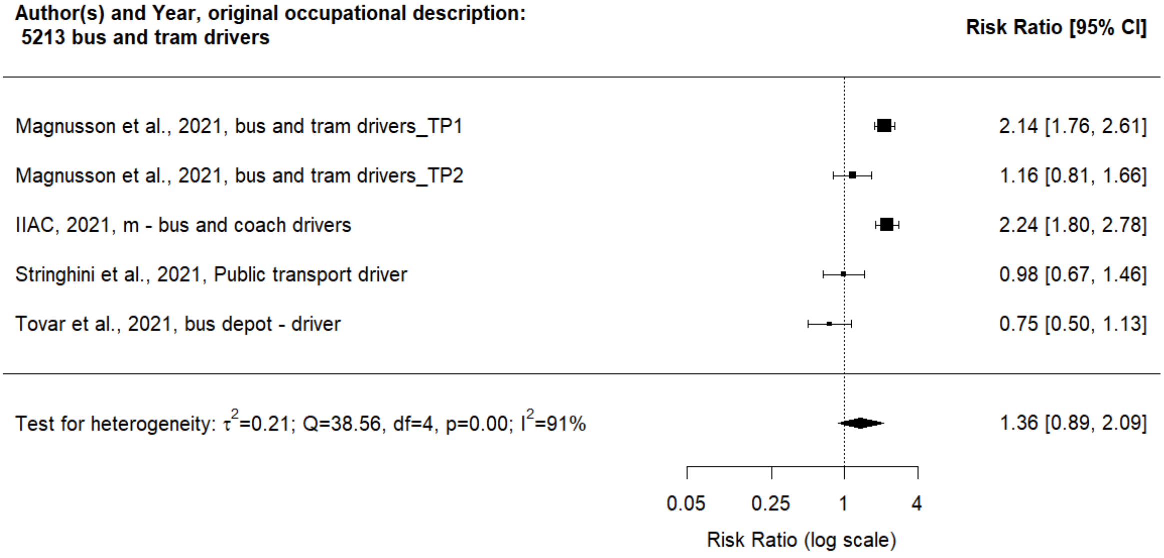

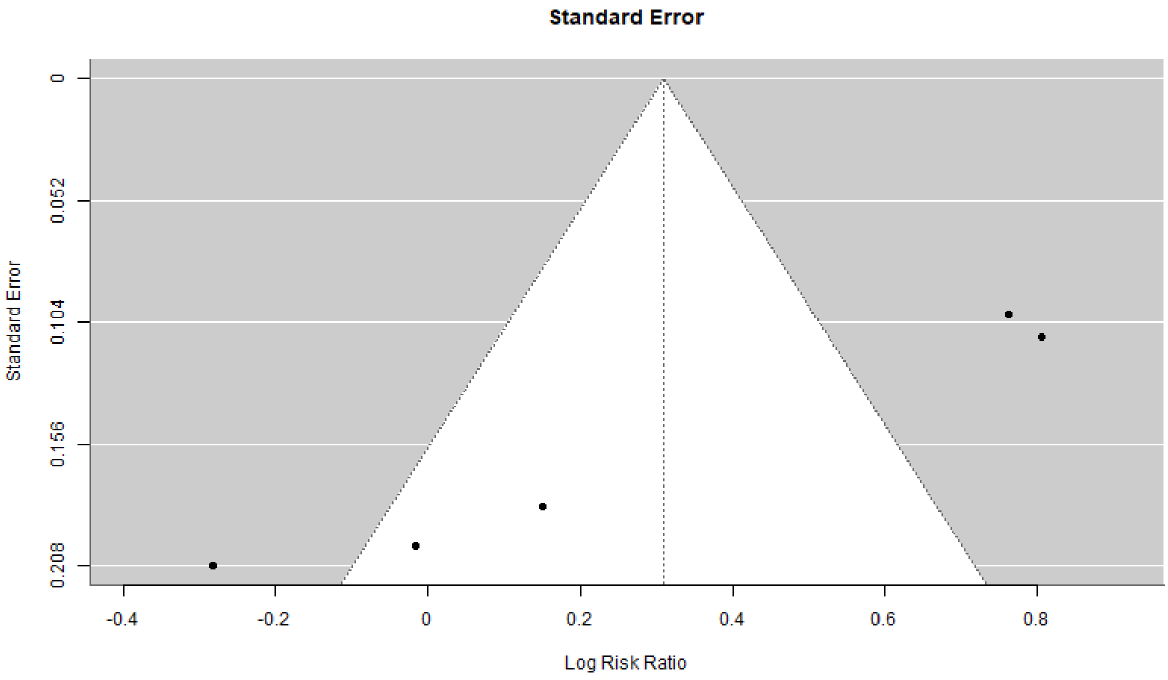

A.4.6 5311 – occupations in physical security, protection of valuables

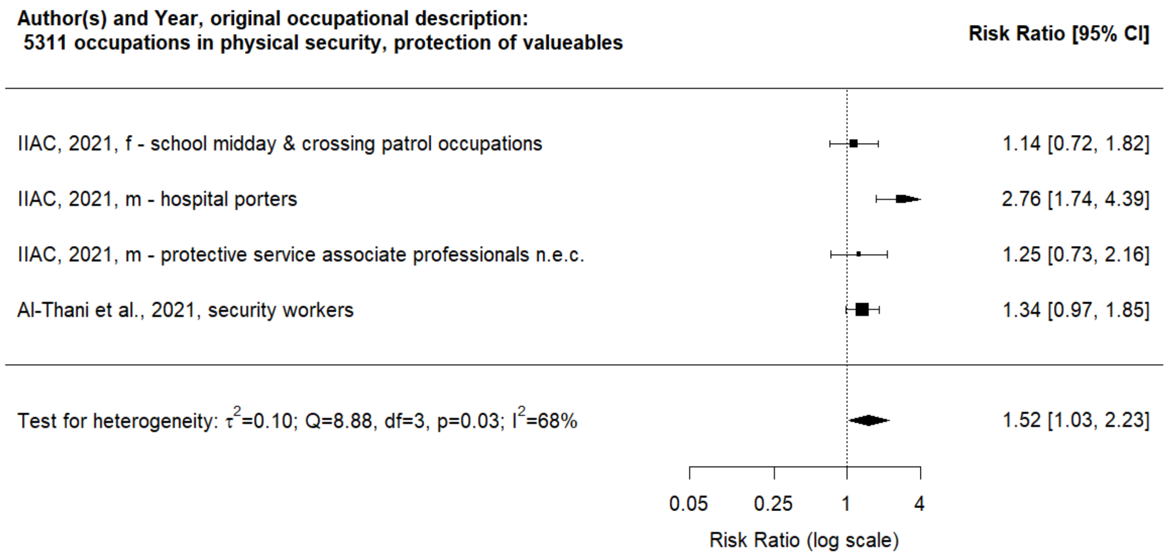

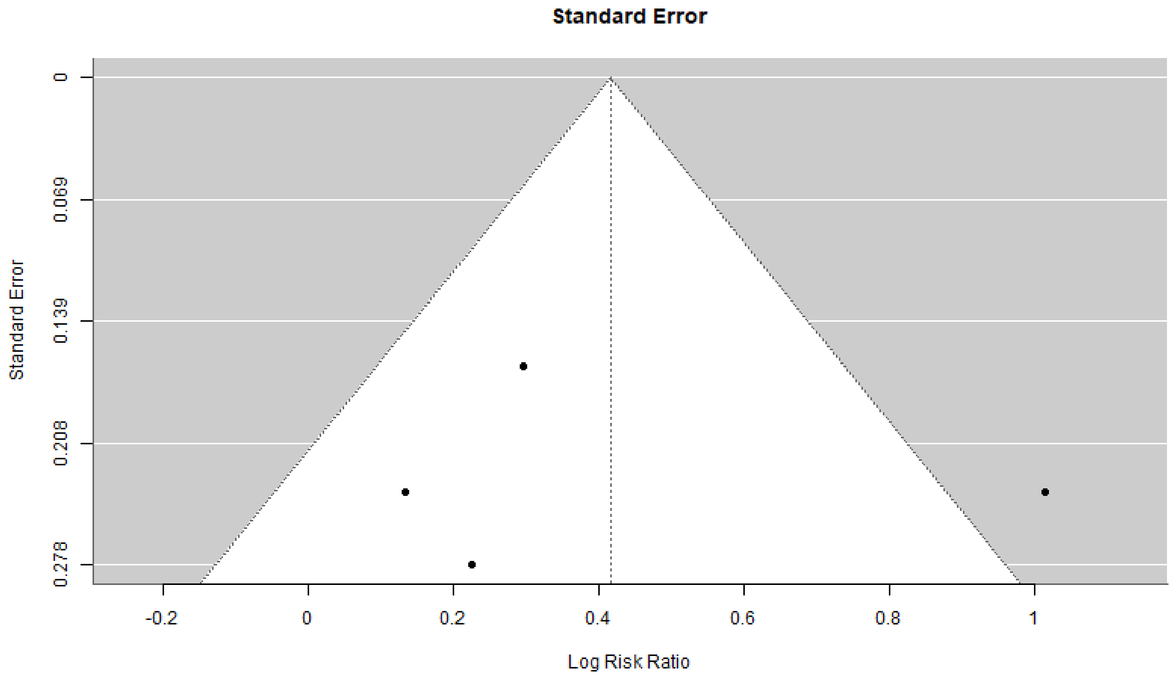

A.4.7 5318 – occupations in physical security, personal protection, fire protection and workplace safety

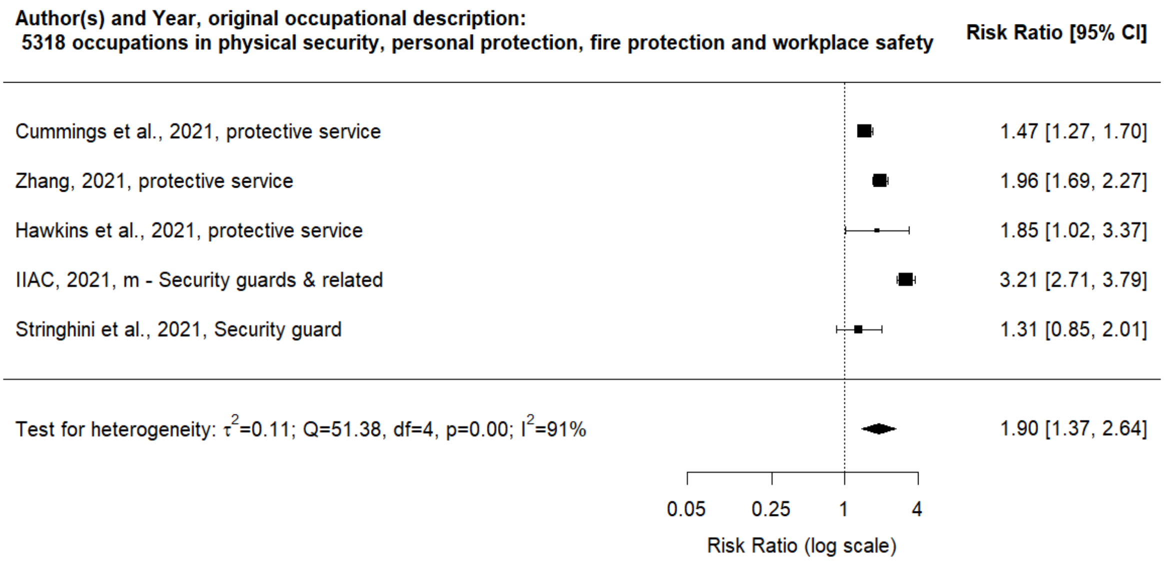

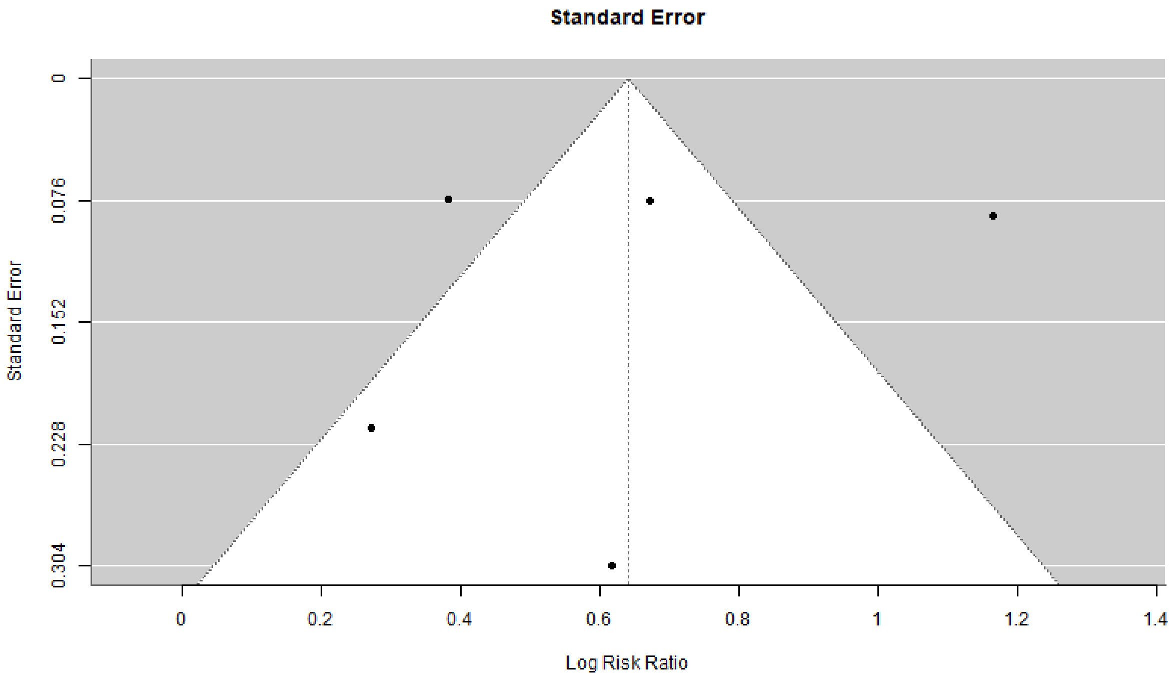

A.4.8 5321 – uniformed police personnel

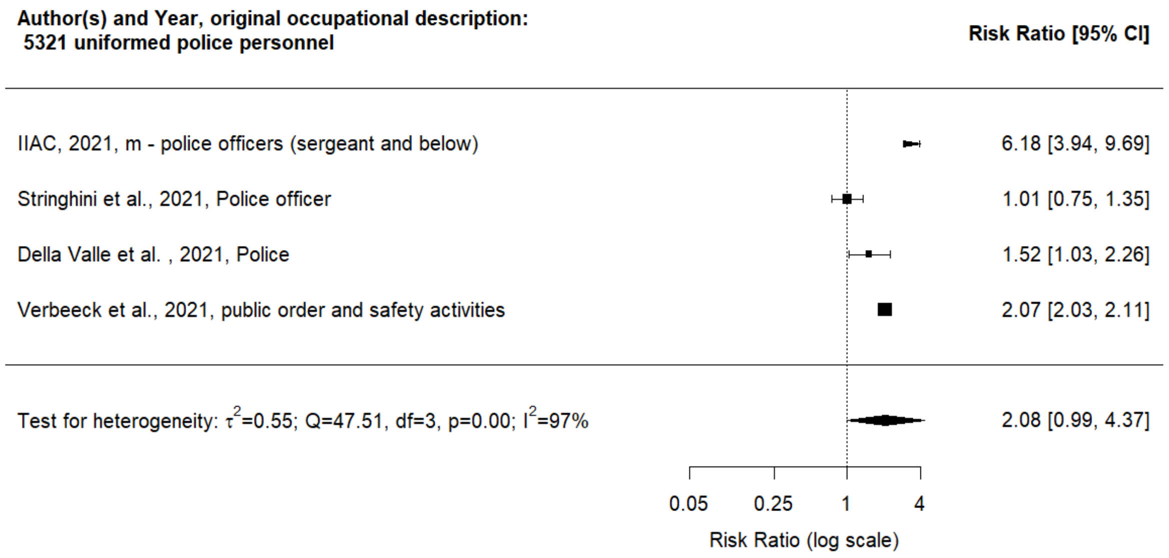

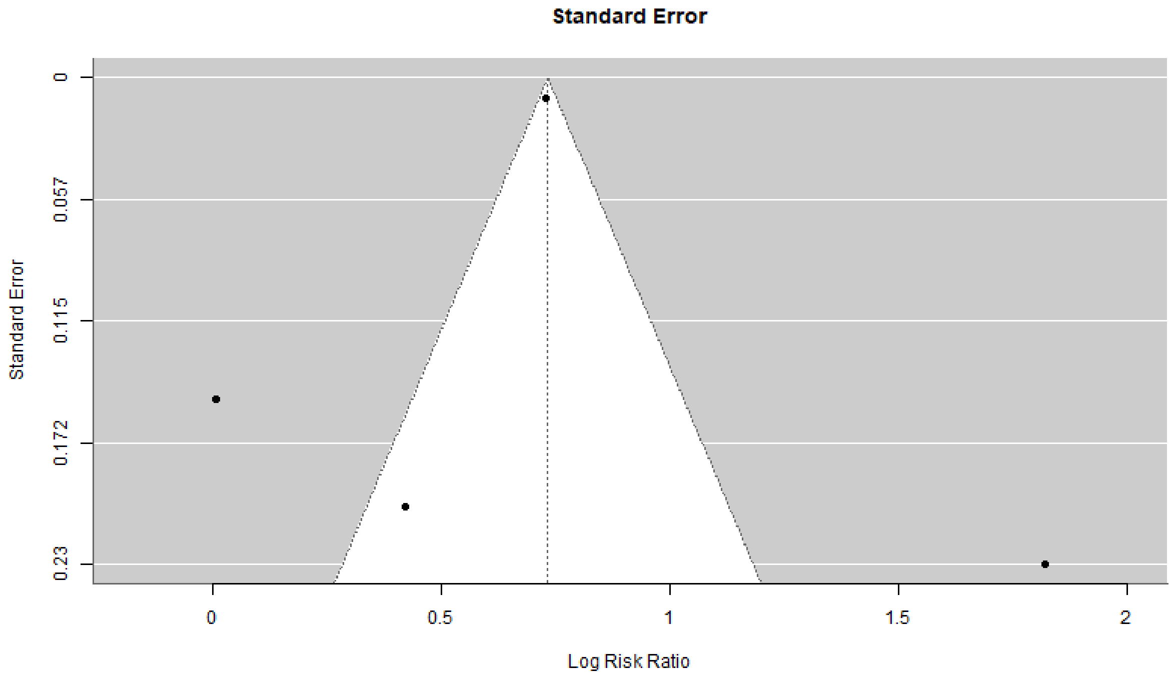

A.4.9 5411 – occupations in building cleaning services

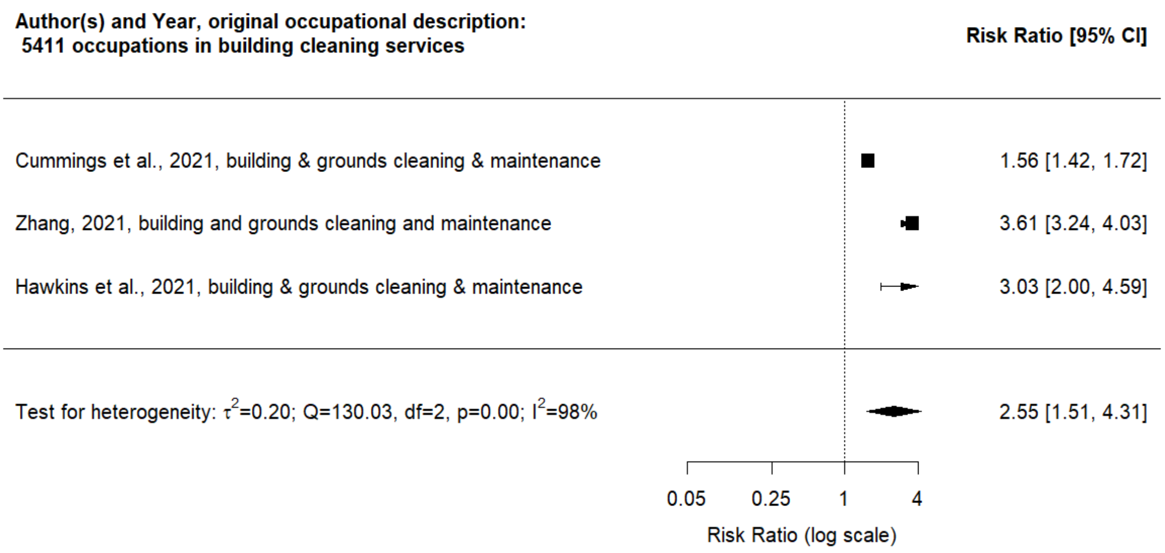

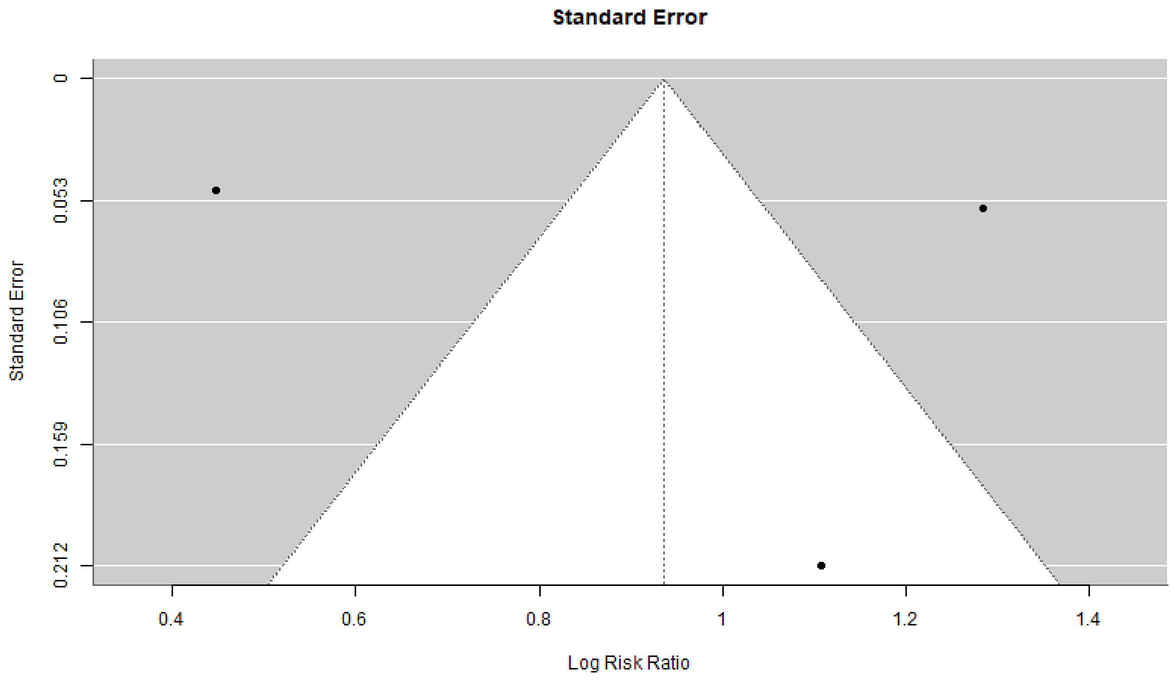

A.4.10 6132 – occupations in facility management

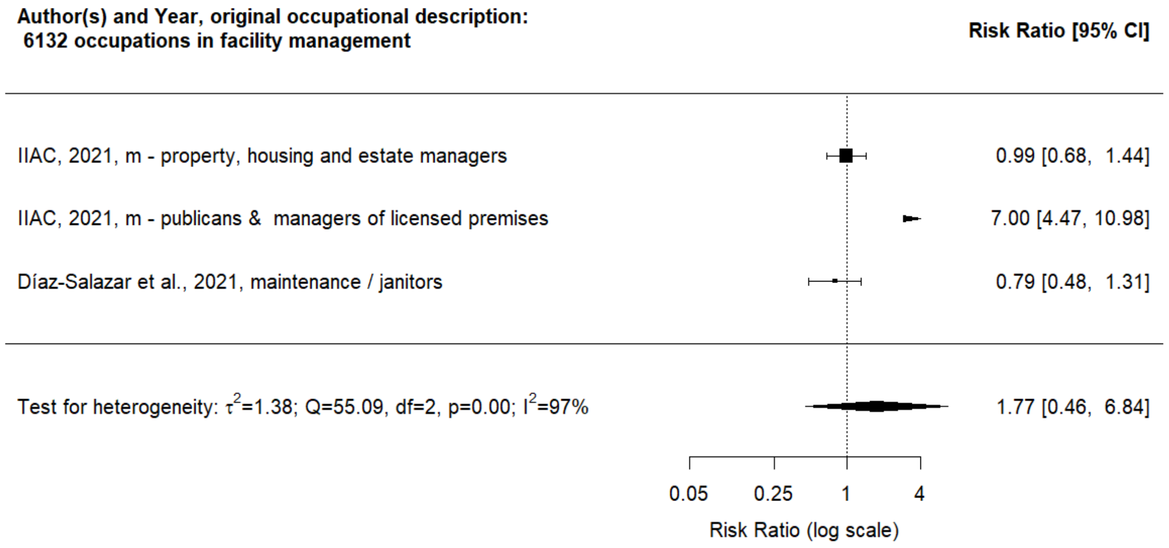

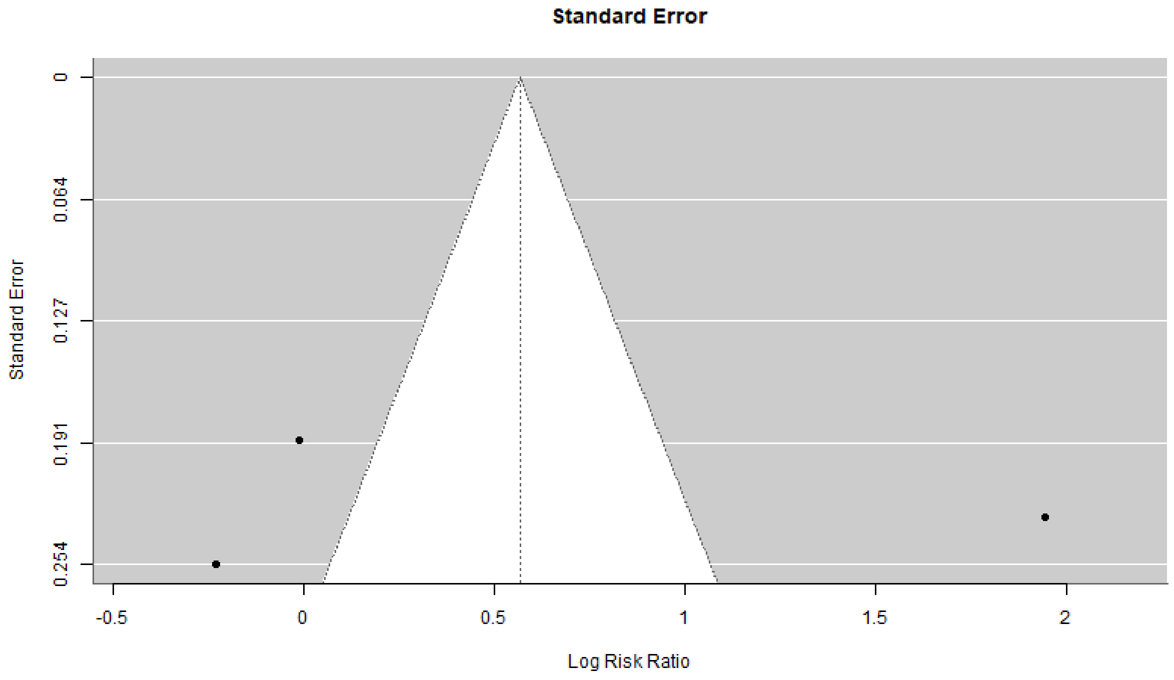

A.4.11 6210 – sales occupations in retail trade (without product specialisation)

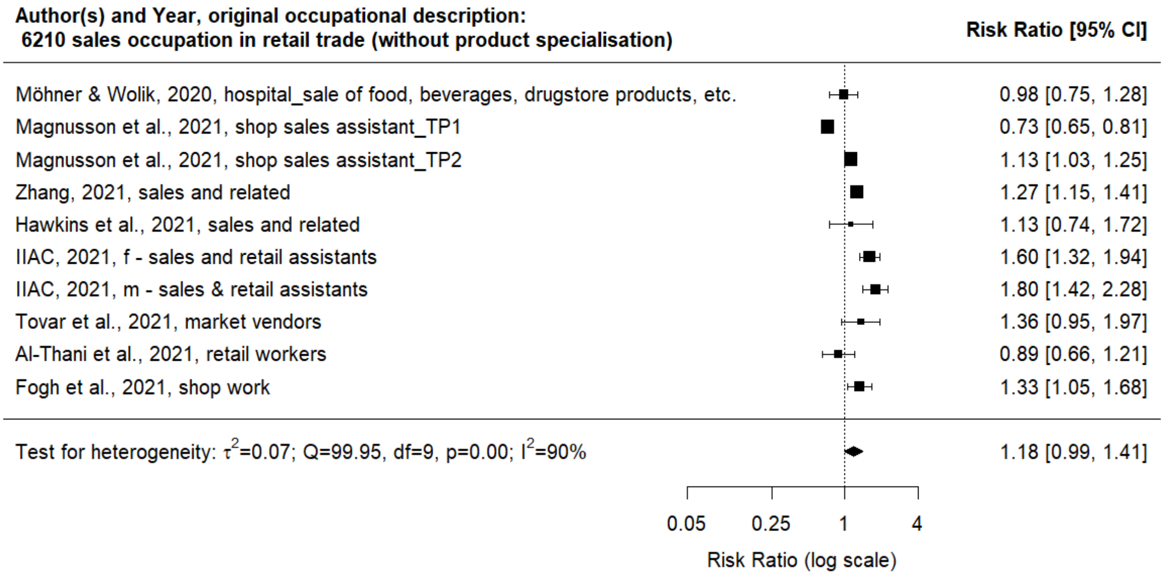

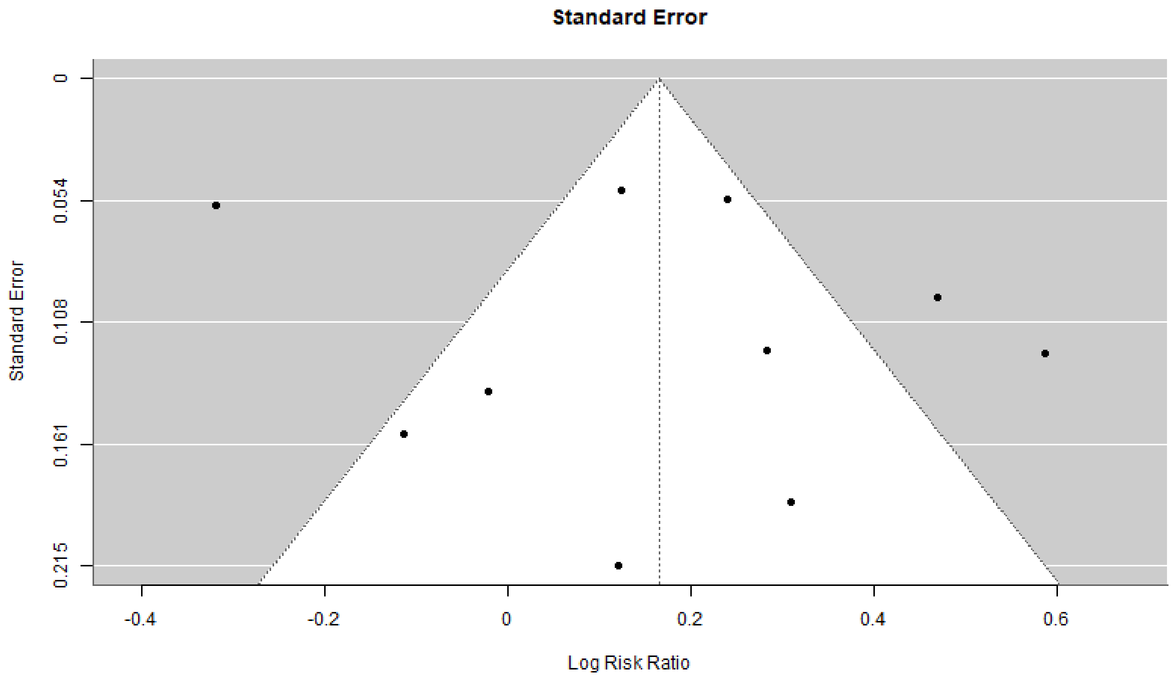

A.4.12 6211 – cashiers and ticket agents

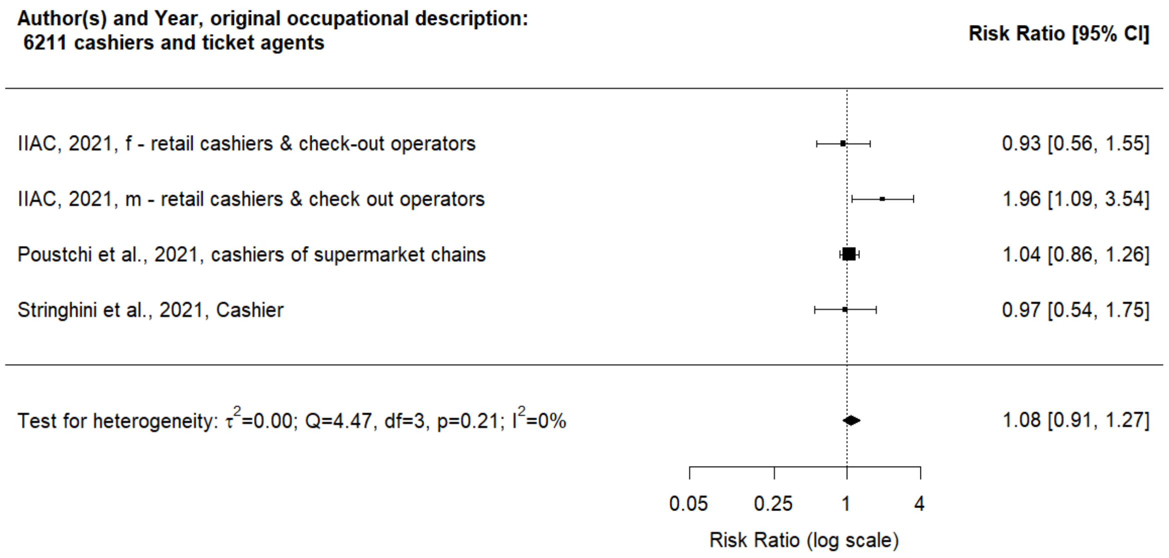

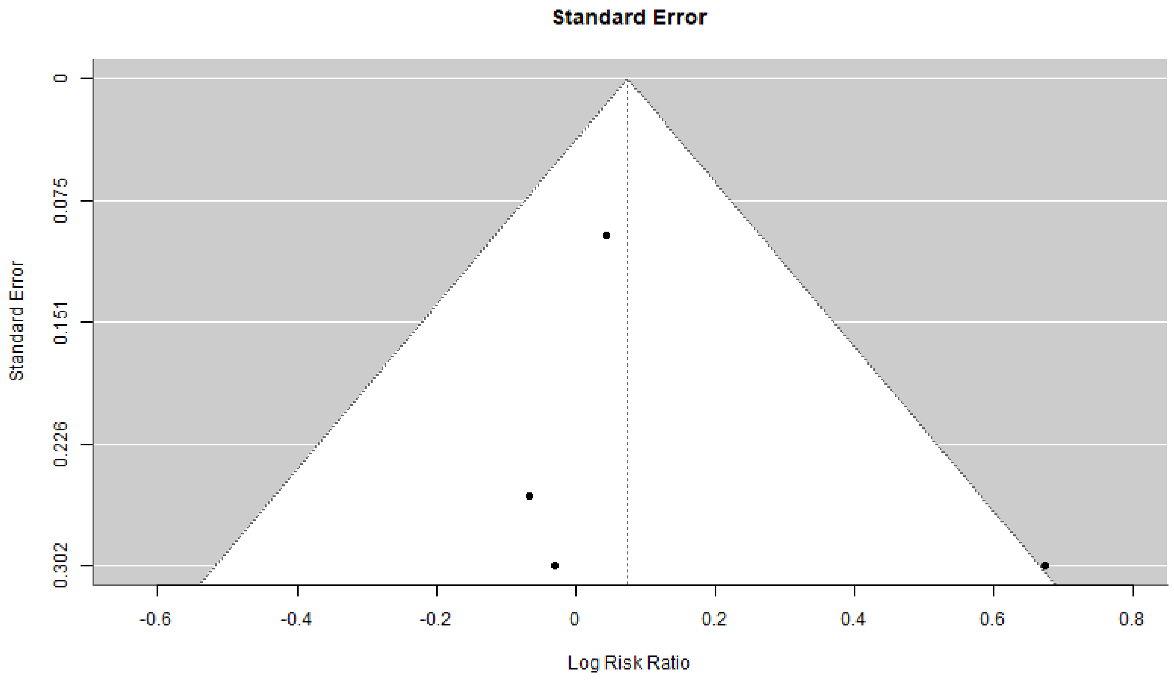

A.4.13 6230 – sales occupations (retail) selling foodstuffs (without specialisation)

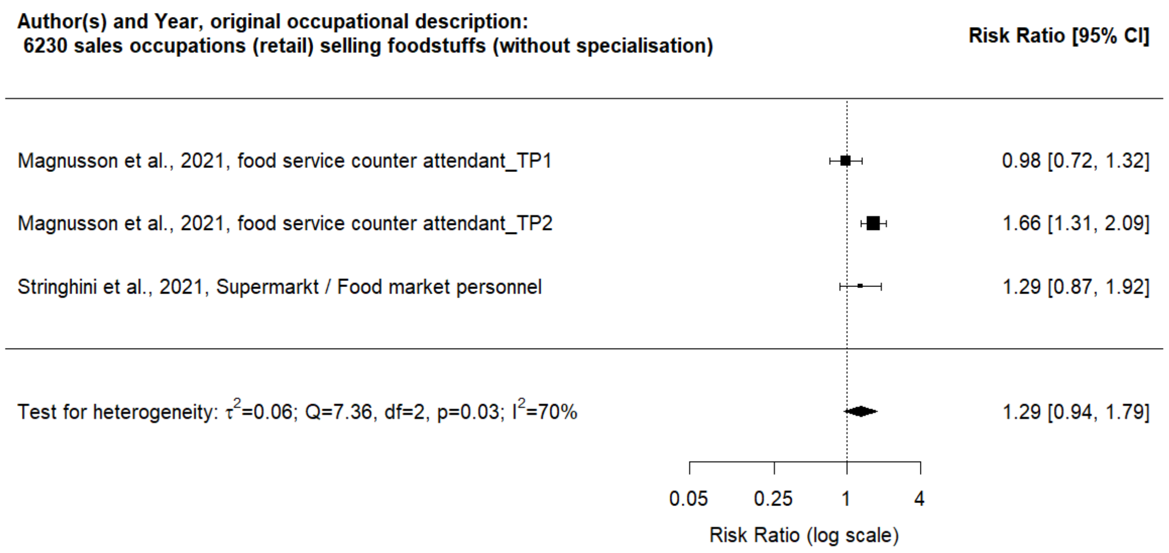

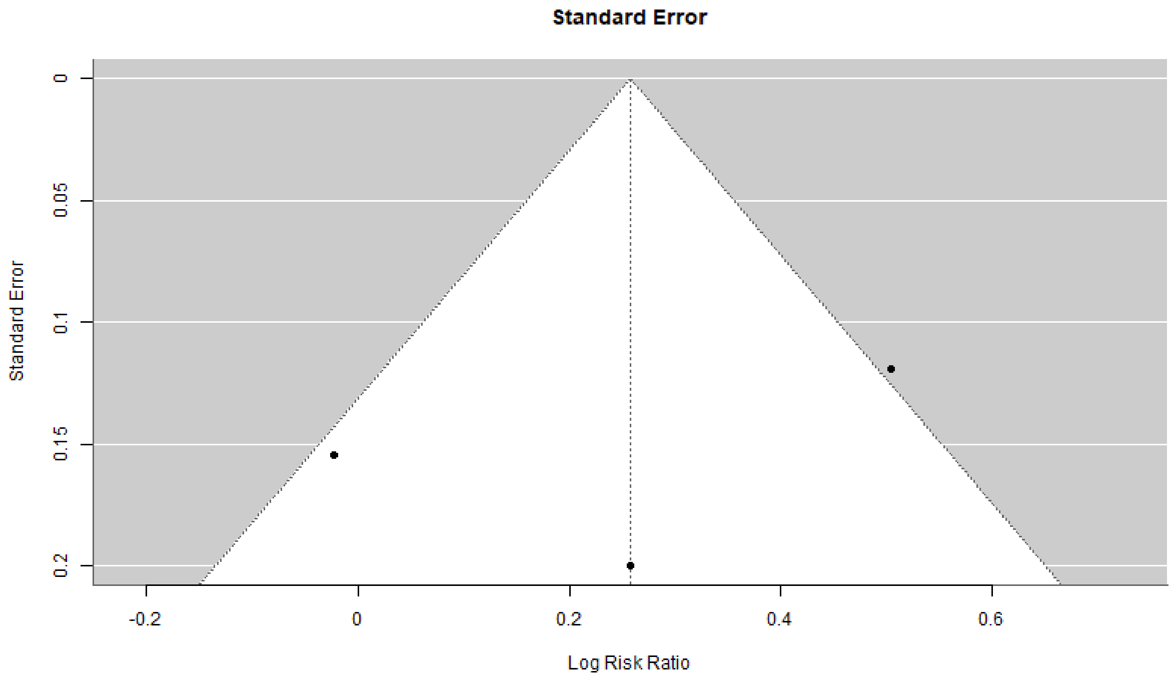

A.4.14 6330 – gastronomy occupations (without specialisation)

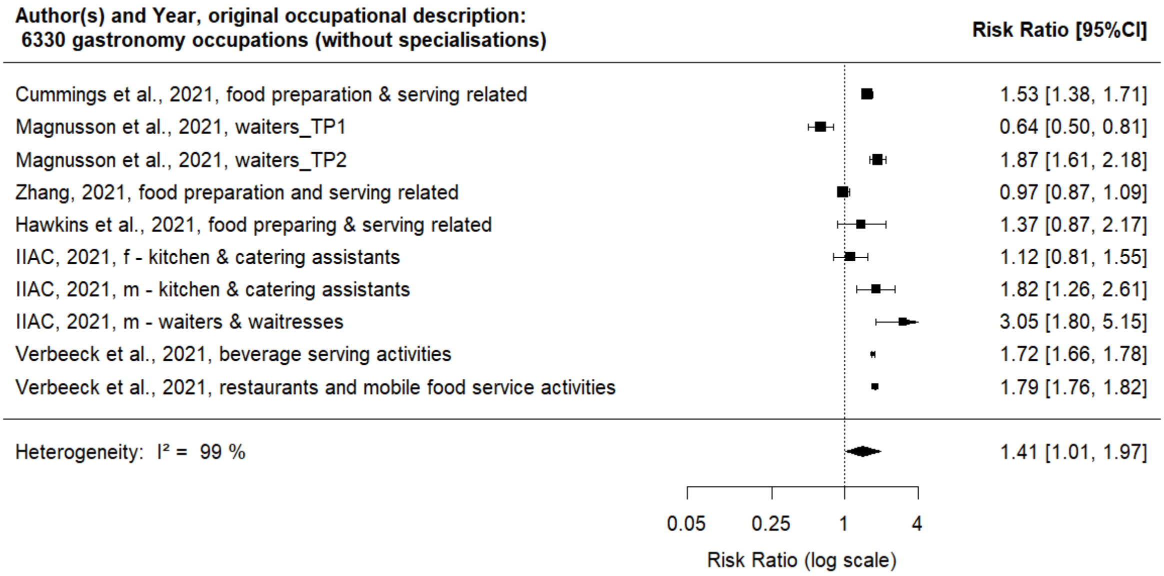

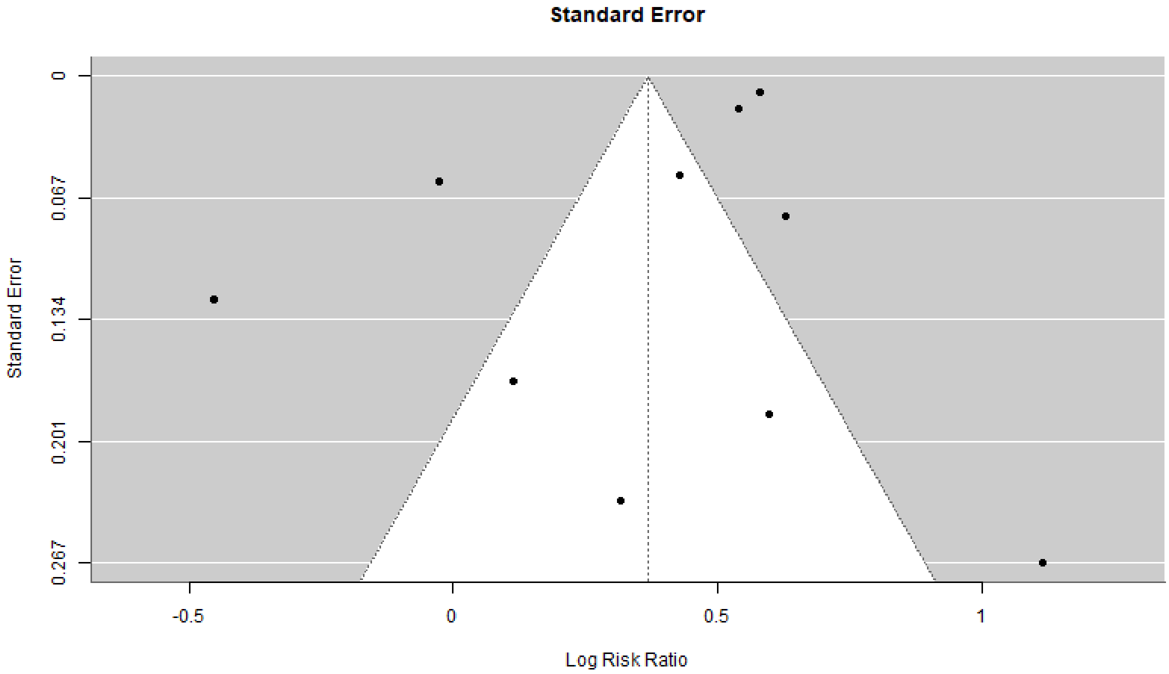

A.4.15 7110 – managing directors and executive board members

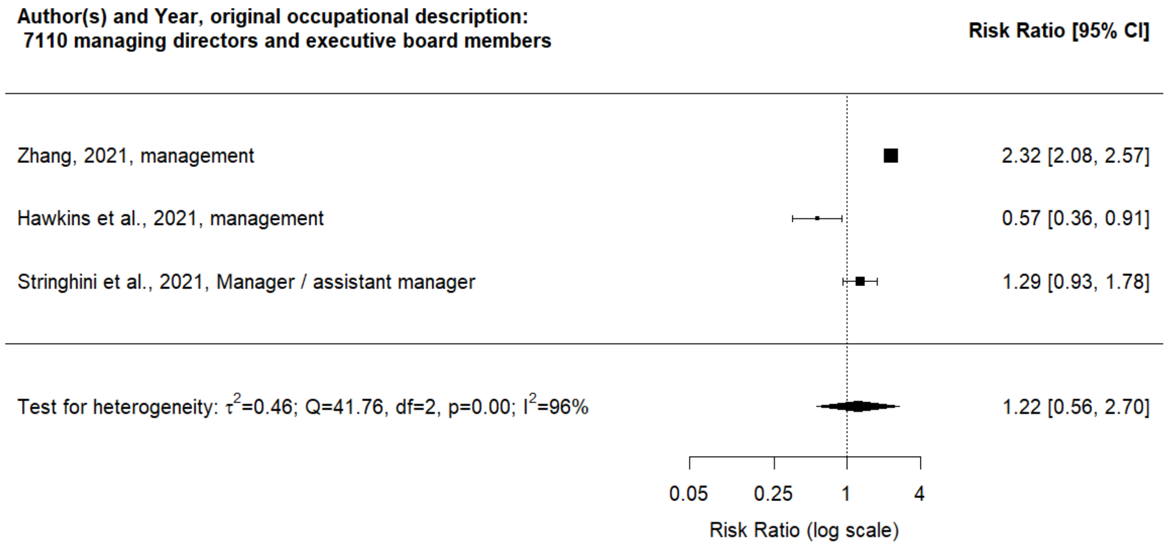

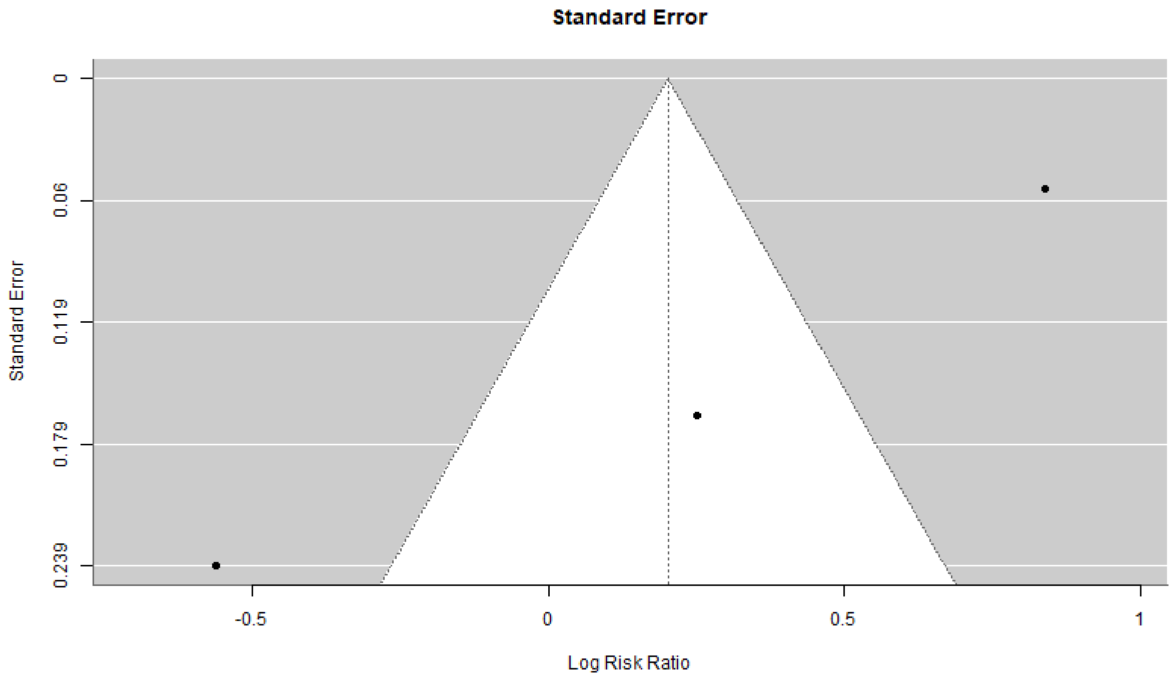

A.4.16 7211 – bankers

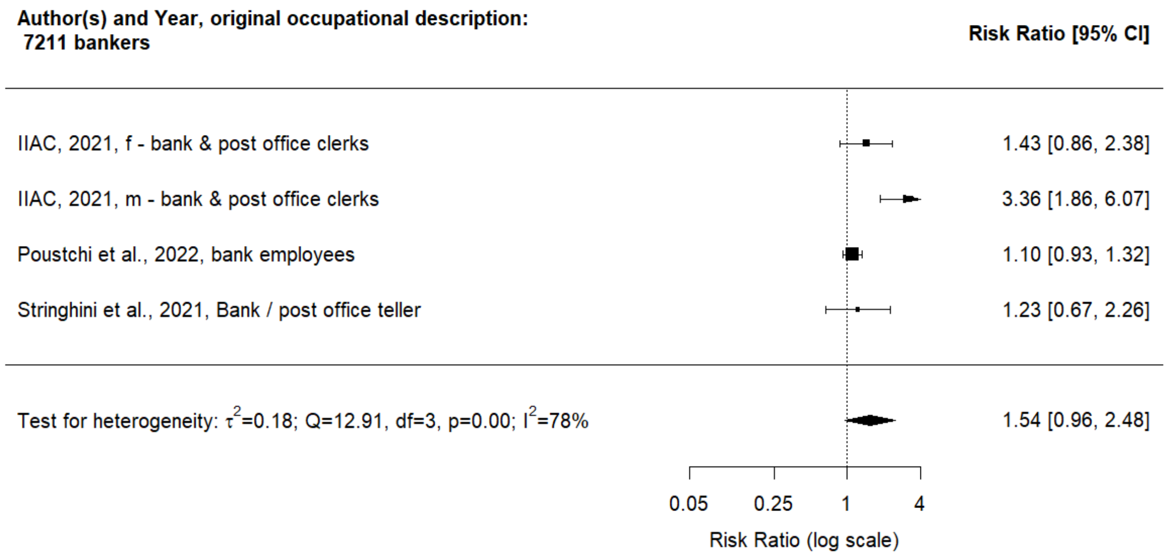

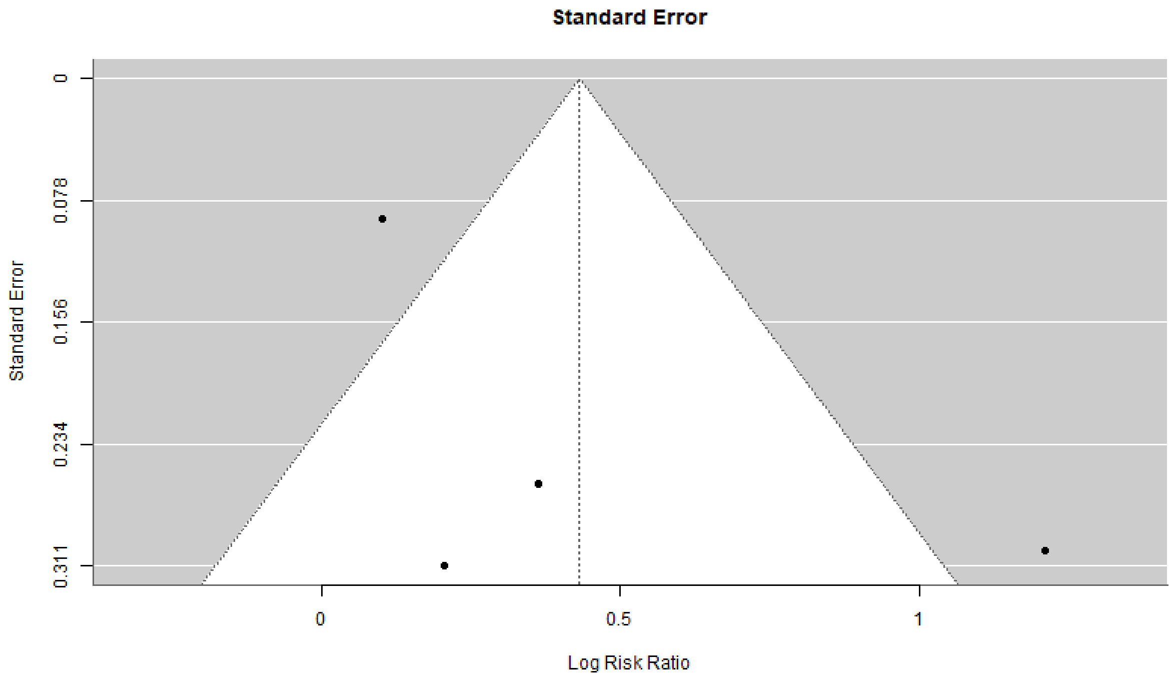

A.4.17 7221 – occupations in accounting

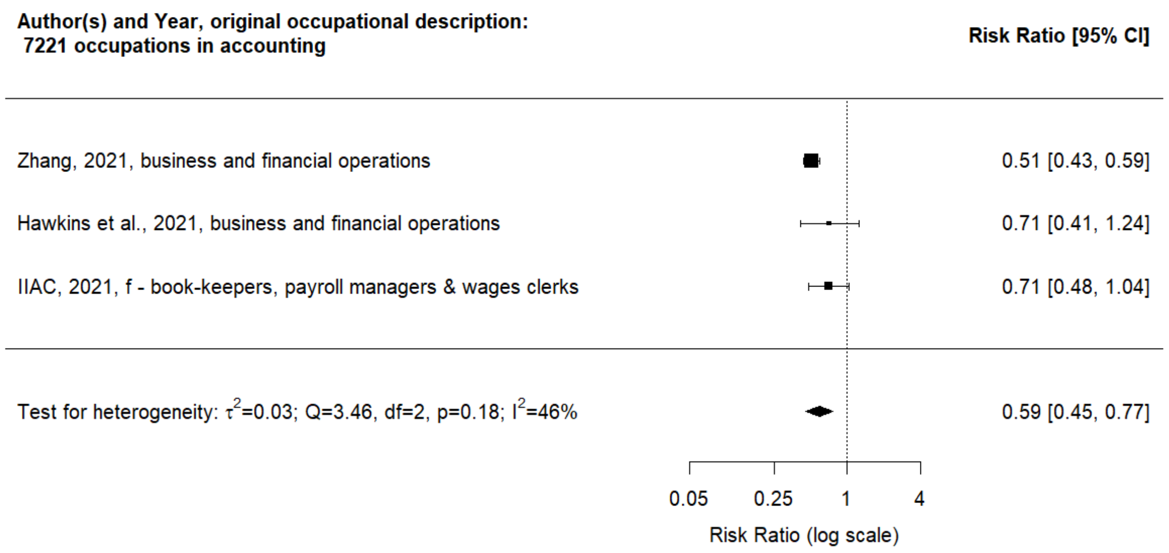

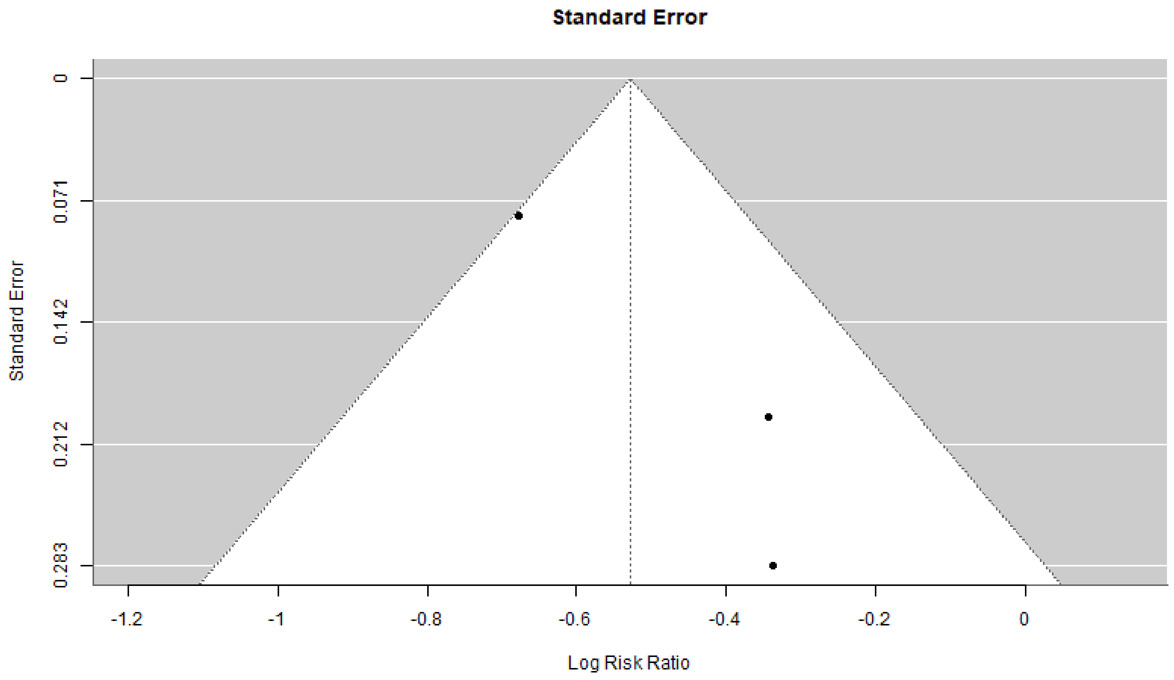

A.4.18 8311 – occupations in child care and child-rearing

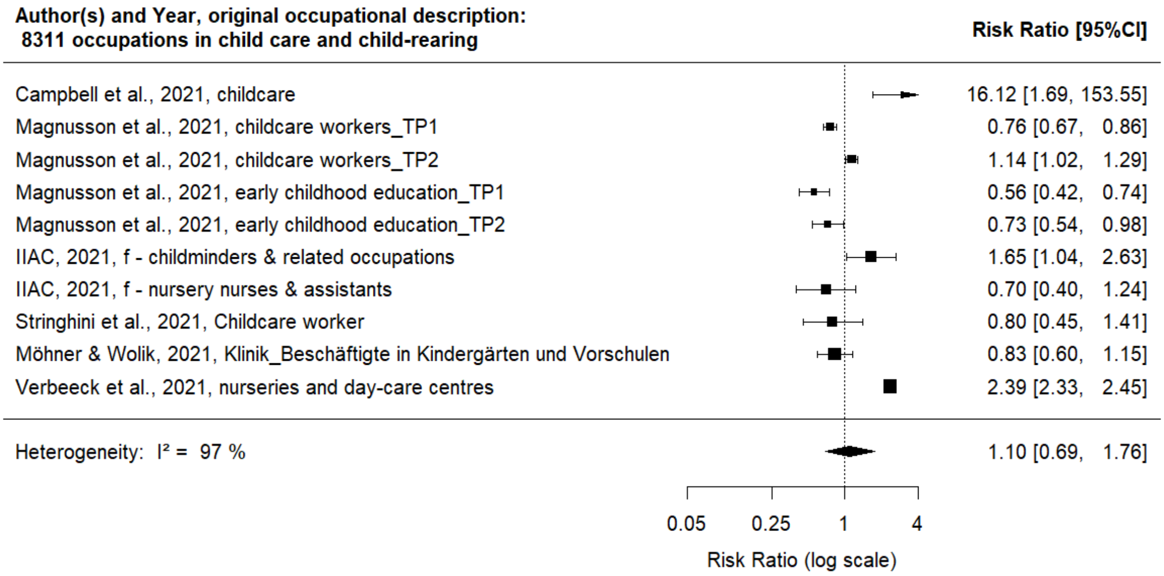

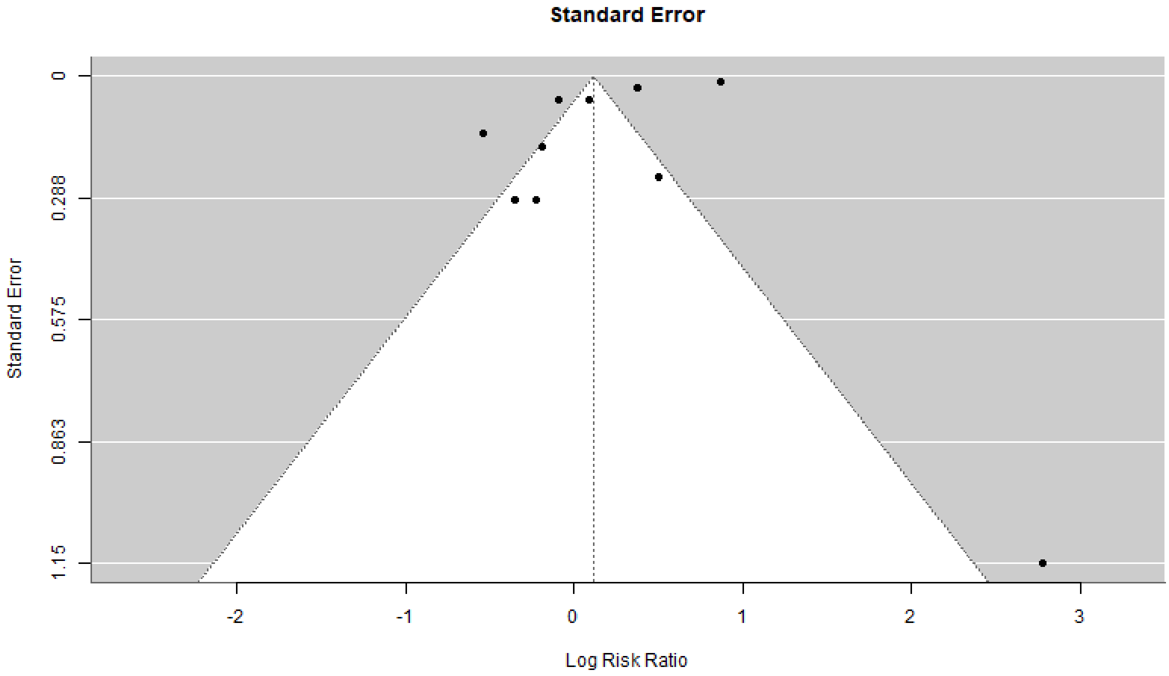

A.4.19 8312 – occupations in social work and social pedagogics

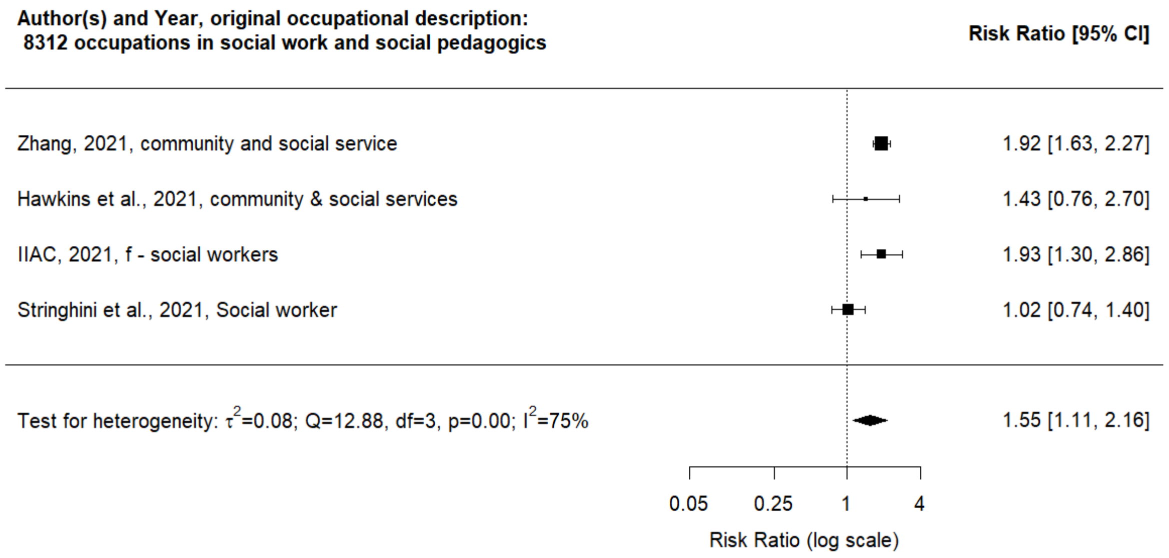

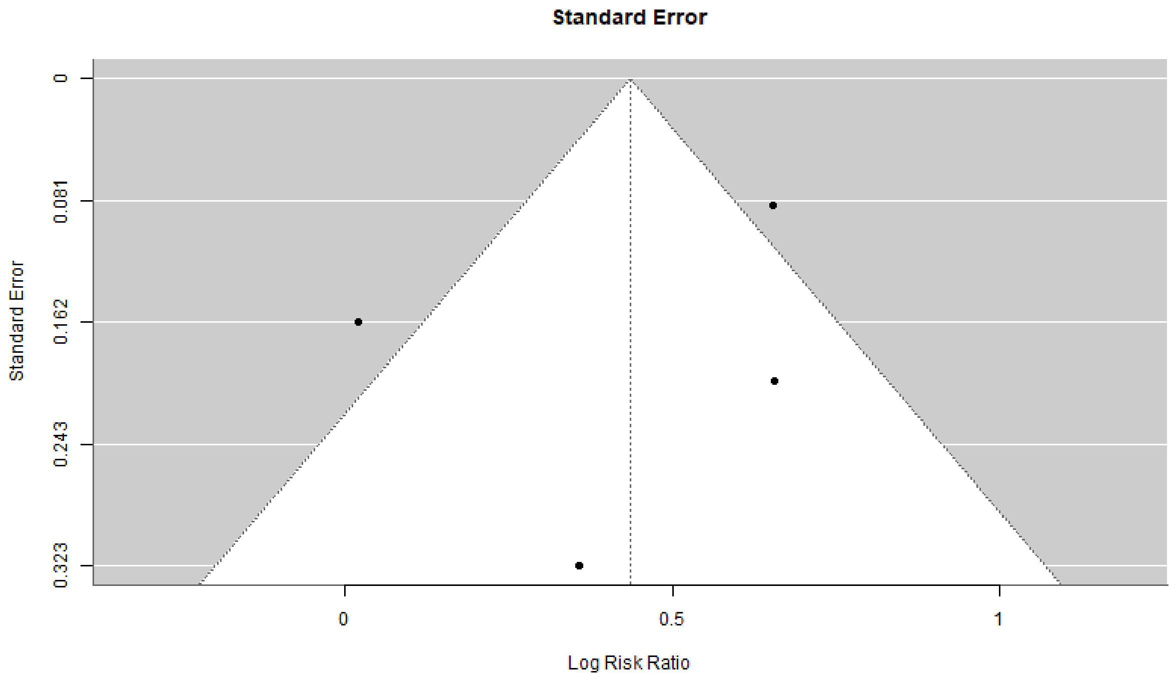

A.4.20 8411 – teachers in primary education

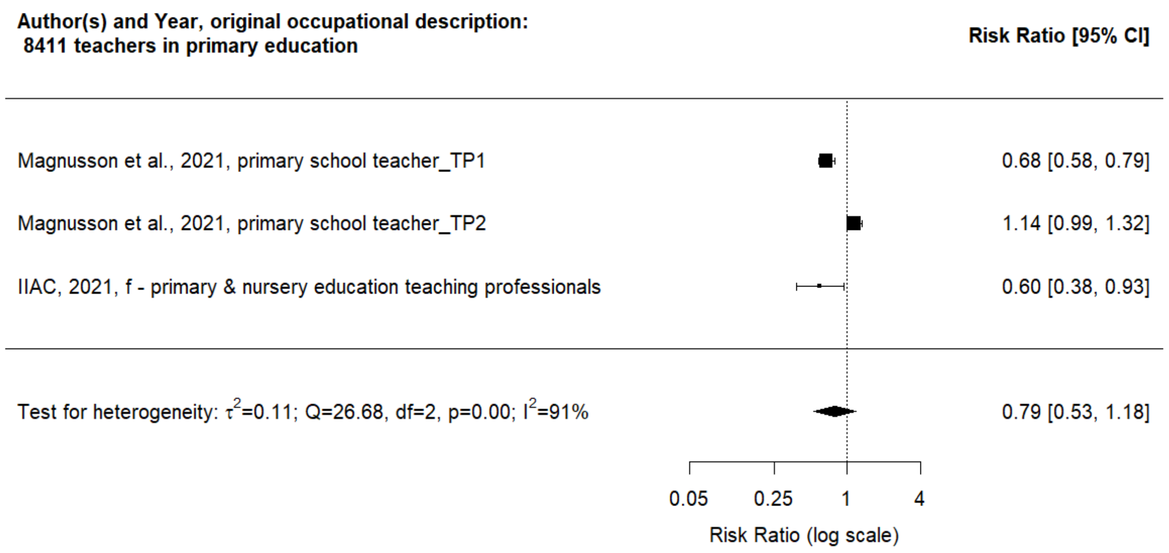

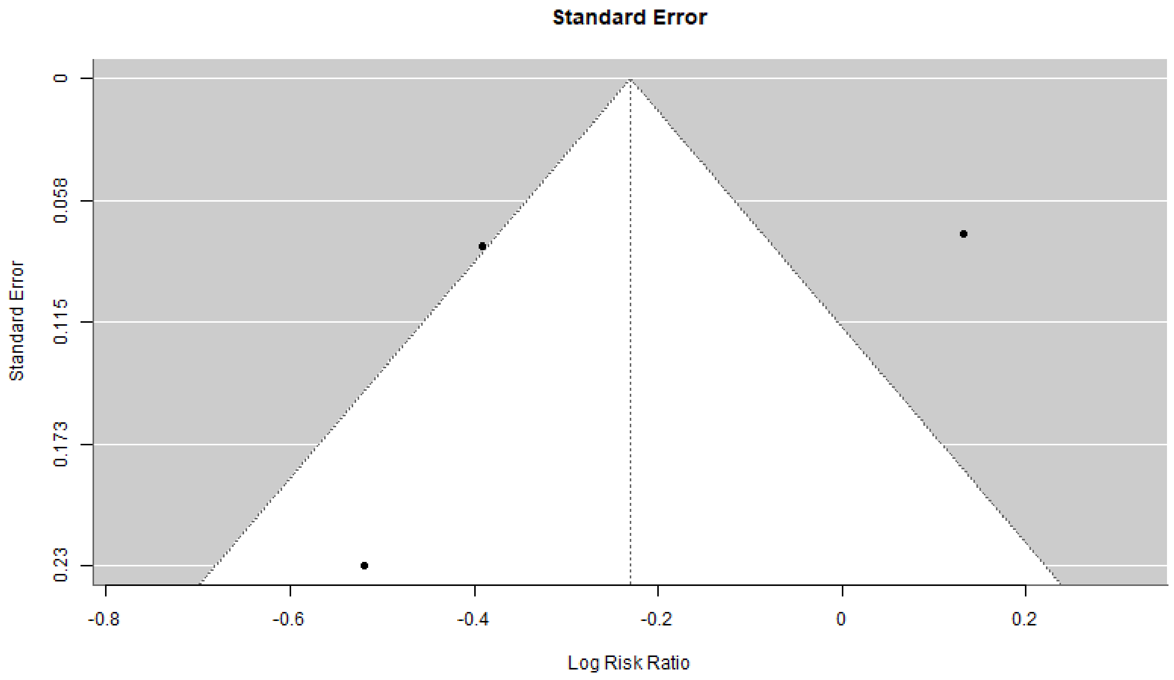

A.5 Subgroup analyses

A.5.1 outcome

	total		infection		hospital		mortality		
2923	3.58	[1.46;8.77]	5.28	[0.70;39.84]	1.47	[0.80;2.70]	6.59	[3.97;10.94]	2
2930	2.53	[1.75;3.67]	1.85	[1.24;2.77]			3.11	[2.45;3.93]	2
5133	2.52	[2.27;2.79]	2.35	[2.14;2.59]			2.73	[2.24;3.33]	2
5211	1.80	[1.19;2.73]	1.30	[1.12;1.52]	2.30	[1.25;4.23]	3.23	[2.81;3.71]	2
5213	1.36	[0.89;2.09]	1.19	[0.76;1.88]			2.24	[1.80;2.78]	2
5311	1.52	[1.03;2.23]	1.34	[0.97;1.85]			1.59	[0.91;2.78]	2
5318	1.90	[1.37;2.64]	1.69	[1.15;2.47]			2.08	[1.25;3.46]	2
5321	2.08	[0.99;4.37]	1.50	[0.97;2.31]			6.18	[3.94;9.69]	2
5411	2.55	[1.51;4.31]	3.61	[3.24;4.03]			2.11	[1.11;4.02]	1
6132	1.77	[0.46;6.84]	0.79	[0.48;1.31]			2.62	[0.39;17.83]	2
6210	1.18	[0.99;1.41]	1.08	[0.88;1.33]	0.98	[0.75;1.28]	1.59	[1.35;1.88]	2
6211	1.08	[0.91;1.27]	1.04	[0.86;1.24]			1.33	[0.64;2.75]	2
6230	1.29	[0.94;1.79]							0
6330	1.41	[1.01;1.97]	1.20	[0.54;2.70]			1.61	[1.22;2.12]	2
7110	1.22	[0.56;2.70]	1.76	[0.99;3.13]			0.57	[0.36;0.91]	1
7211	1.54	[0.96;2.48]	1.11	[0.94;1.32]			2.16	[0.94;4.98]	2
7221	0.59	[0.45;0.77]	0.51	[0.43;0.59]			0.71	[0.52;0.97]	2
8311	1.10	[0.69;1.76]	1.24	[0.49;3.15]	0.83	[0.60;1.15]	1.10	[0.47;2.54]	1
8312	1.55	[1.11;2.16]	1.42	[0.76;2.65]			1.77	[1.27;2.48]	2
8411	0.79	[0.53;1.18]	0.88	[0.53;1.47]			0.60	[0.38;0.93]	1
1	infection > mortality								
2	mortality > infection								

A.5.2 comparison group

	total		admin		non-ess.		other		gen pop		
2923	3.58	[1.46;8.77]					5.28	[0.70;39.84]	3.14	[0.72;13.68]	0
2930	2.53	[1.75;3.67]	1.85	[1.24;2.77]					3.11	[2.45;3.93]	2
5133	2.52	[2.27;2.79]	2.65	[1.92;3.66]			2.59	[2.34;2.87]			0
5211	1.80	[1.19;2.73]	1.36	[0.53;3.49]					1.87	[1.17;2.99]	2
5213	1.36	[0.89;2.09]	0.87	[0.65;1.15]					1.82	[1.23;2.68]	2
5311	1.52	[1.03;2.23]	1.34	[0.97;1.85]					1.59	[0.91;2.78]	2
5318	1.90	[1.37;2.64]	1.76	[1.36;2.28]			1.47	[1.27;1.70]	3.21	[2.71;3.79]	2
5321	2.08	[0.99;4.37]	1.21	[0.81;1.82]					3.49	[1.20;10.19]	2
5411	2.55	[1.51;4.31]	3.57	[3.21;3.97]			1.56	[1.42;1.72]			0
6132	1.77	[0.46;6.84]	0.79	[0.48;1.31]					2.62	[0.39;17.83]	2
6210	1.18	[0.99;1.41]	1.23	[1.10;1.37]					1.18	[0.85;1.63]	1
6211	1.08	[0.91;1.27]	0.97	[0.54;1.75]					1.17	[0.82;1.66]	2
6230	1.29	[0.94;1.79]	1.29	[0.87;1.92]					1.28	[0.77;2.15]	1
6330	1.41	[1.01;1.97]	1.07	[0.79;1.45]			1.53	[1.38;1.71]	1.51	[0.87;2.60]	2
7110	1.22	[0.56;2.70]									0
7211	1.54	[0.96;2.48]	1.23	[0.67;2.26]					1.67	[0.88;3.17]	2
7221	0.59	[0.45;0.77]	0.54	[0.42;0.70]					0.71	[0.48;1.04]	2
8311	1.10	[0.69;1.76]	0.80	[0.45;1.40]			16.12	[1.69;153.55]	1.06	[0.61;1.84]	2
8312	1.55	[1.11;2.16]	1.44	[0.94;2.19]					1.93	[1.30;2.86]	2
8411	0.79	[0.53;1.18]									0
1	admin > general population										
2	general population > admin										

A.5.3 risk of bias

	total		low		high		
2923	3.58	[1.46;8.77]	6.59	[3.97;10.94]	2.64	[2.64;8.77]	1
2930	2.53	[1.75;3.67]	3.11	[2.45;3.93]	1.85	[1.24;3.67]	1
5133	2.52	[2.27;2.79]					0
5211	1.80	[1.19;2.73]	1.77	[0.97;3.23]	1.97	[1.18;3.28]	2
5213	1.36	[0.89;2.09]	1.82	[1.23;2.68]	0.87	[0.65;1.15]	1
5311	1.52	[1.03;2.23]	1.59	[0.91;2.78]	1.34	[0.97;1.85]	1
5318	1.90	[1.37;2.64]	3.21	[2.71;3.79]	1.64	[1.34;2.02]	1
5321	2.08	[0.99;4.37]	6.18	[3.94;9.69]	1.50	[0.97;2.31]	1
5411	2.55	[1.51;4.31]					0
6132	1.77	[0.46;6.84]	2.62	[0.39;17.83]	0.79	[0.48;1.31]	1
6210	1.18	[0.99;1.41]	1.23	[0.83;1.83]	1.17	[1.03;1.34]	1
6211	1.08	[0.91;1.27]	1.33	[0.64;2.75]	1.04	[0.86;1.24]	1
6230	1.29	[0.94;1.79]	1.28	[0.77;2.15]	1.29	[0.87;1.92]	2
6330	1.41	[1.01;1.97]	1.46	[0.87;2.44]	1.38	[0.88;2.17]	1
7110	1.22	[0.56;2.70]					0
7211	1.54	[0.96;2.48]	2.16	[0.94;4.98]	1.11	[0.94;1.32]	1
7221	0.59	[0.45;0.77]	0.71	[0.48;1.04]	0.54	[0.42;0.70]	1
8311	1.10	[0.69;1.76]	0.89	[0.50;1.58]	1.56	[0.64;3.80]	2
8312	1.55	[1.11;2.16]	1.93	[1.30;2.86]	1.44	[0.94;2.19]	1
8411	0.79	[0.53;1.18]					0
1	low RoB > high RoB						
2	high RoB > low RoB						
