# Supplementary material for: SARS-CoV-2 infection risk by non-healthcare occupations: a systematic review and meta-analysis
Source: J Occup Med Toxicol. 2025 May 22;20:17. doi: 10.1186/s12995-025-00462-9 (PMC12096541; doi:10.1186/s12995-025-00462-9)
Supplement: Supplementary file 2 — Supplementary Material 2. [file 12995_2025_462_MOESM2_ESM.pdf]

Appendix B

<table>
<tr><th>Reference</th><th>Study Design</th><th>Population</th><th>Out-come</th><th>Exposure</th><th>Results</th><th>Comments</th></tr>
<tr><td></td><td></td><td></td><td></td><td></td><td></td><td></td></tr>
<tr>
<td rowspan="6">Al-Thani, 2020</td>
<td rowspan="6">Cross-sectional</td>
<td>Study region:
Qatar</td>
<td rowspan="6">Infection (IgM/ IgG, PCR)</td>
<td rowspan="2">Investigated occupation:
Diverse (n=9 occupational sectors)</td>
<td rowspan="6">
<table>
<tr><th></th><th>Persons tested</th><th>Positive Result</th></tr>
<tr><td>administration workers</td><td>82</td><td>36</td></tr>
<tr><td>professional workers</td><td>137</td><td>36</td></tr>
<tr><td>food and beverage workers</td><td>93</td><td>26</td></tr>
<tr><td>retail workers</td><td>171</td><td>67</td></tr>
<tr><td>transport workers</td><td>435</td><td>227</td></tr>
<tr><td>cleaning workers</td><td>105</td><td>55</td></tr>
<tr><td>technical and construction workers</td><td>1329</td><td>862</td></tr>
<tr><td>security workers</td><td>61</td><td>36</td></tr>
</table>
</td>
<td rowspan="6">- PCR and IgM/IgG tests were able to detect both previous and current infections
- only men were interviewed</td>
</tr>
<tr><td>Sample population: The study's sample population consisted of craft and manual workers (CMWs) in Qatar, who make up 60% of the country's total population. The participants were predominantly from India (29.2%), Bangladesh (26.2%), and Nepal (21.6%), reflecting the broader CMW population in the country</td></tr>
<tr><td>Sample size:
2.641</td><td rowspan="2">Assessment:
Interview</td></tr>
<tr><td>Age:
Md 35
IQR [18-80]</td></tr>
<tr><td>Time of study:
07/2020 – 09/2020</td><td>Time of occupation assessment:
07/2020 – 09/2020</td></tr>
<tr><td>Response:
>90%</td><td></td></tr>
</table>

<table>
<tr><td></td><td></td><td></td><td></td><td></td><td><table><tr><td>Other workers</td><td>178</td><td>64</td></tr></table></td><td></td></tr>
<tr>
<td>Billingsley, 2022</td>
<td>Cohort</td>
<td>Study region:
Sweden

Sample population:
The study determined COVID-19-related mortality as the outcome using Sweden's National Cause of Death Register. Deaths were classified as COVID-19-related based on ICD-10 codes (U07.1, U07.2, and B3.42). The data included both deaths where COVID-19 was listed as the underlying cause and deaths where it was a contributing cause.

Sample size:
4 620 395

Age:
MW = 43.2

Time of outcome:</td>
<td>Mortality (ICD-10: U07.1, U07.2, B3.42)</td>
<td>Investigated occupation:
diverse (n=9 occupational sectors)

Assessment:
Standard för svensk yrkesklassificering (SSYK, Swedish administrative occupational registers)

Time of occupation assessment:</td>
<td><table>
<tr><td></td><td></td><td>Exposed</td><td>Deaths</td></tr>
<tr><td>care workers</td><td>221, 222, 223, 532, 533, 534; excluding 2222, 2225, 2233, 2234</td><td>2.824.735</td><td>265</td></tr>
<tr><td>taxi & bus drivers</td><td>8321, 8331</td><td>601.616</td><td>37</td></tr>
<tr><td>meat packers</td><td>7611</td><td>38.046</td><td>25</td></tr>
<tr><td>teachers</td><td>234, 5311</td><td>3.409</td><td>< 5</td></tr>
<tr><td>service sector</td><td>522, 941, 523</td><td>310.084</td><td>16</td></tr>
<tr><td>police men, security guards</td><td>5412, 5413, 3360</td><td>347.283</td><td>22</td></tr>
<tr><td>postal workers, delivery</td><td>4420, 8329</td><td>45.004</td><td>< 5</td></tr>
</table></td>
<td>- Study with nationwide register data
- Occupations are based on December 2018 data, which may not reflect job changes or exits from the labour market during the pandemic.
- The outcome is mortality, which may be influenced by other factors (e.g., pre-existing conditions)</td>
</tr>
</table>

<table>
<tr><td></td><td></td><td>03/2020 – 02/2021

Response:
Register-Study</td><td></td><td>12/18</td><td><table><tr><td>cleaners</td><td>9111</td><td>27.722</td><td>< 5</td></tr><tr><td>IT, economics, or admin (skilled)</td><td>241, 242, 243, 251</td><td>86.414</td><td>14</td></tr></table></td><td></td></tr>
<tr><td>Campbell</td><td>Cross-sectional</td><td>Studio region:
Canada

Sample population:
The people examined were essential workers from 69 non-healthcare businesses.

Sample size:
6.607

Age:
Median: 48
Range: 37-57

Time of outcome:
01/21-03/21

Response:
90,6%</td><td>Infection (PCR test)</td><td>Investigated occupations:
Diverse (n=4 occupational sectors)

Assessment:
Study was conducted at workplace

Time of occupation assessment:
01/21-03/21</td><td><table><tr><td>Occupation</td><td>N</td><td>Infected</td></tr><tr><td>other</td><td>607</td><td>1</td></tr><tr><td>manufacturing / supplier</td><td>1225</td><td>43</td></tr><tr><td>meat processing</td><td>183</td><td>6</td></tr><tr><td>childcare</td><td>113</td><td>3</td></tr></table></td><td>- Only essential businesses willing to participate were included.
- High response among participants, but only 19% of the requested businesses
- Repeat tests in companies if 2 or more people were positive at this company
- High participation was attributed to on-site sampling</td></tr>
<tr><td>Cummings, 2021</td><td>Cohort</td><td>Study region:
USA

Sample population:
The sample population consisted of</td><td>Mortality (death certificates)</td><td>Investigated occupation:
diverse (n=23 occupational sectors)</td><td><table><tr><td></td><td>N</td><td>Infected</td></tr><tr><td>overall</td><td>20756667</td><td>6227</td></tr></table></td><td>- It was a register-based study, which means it includes all COVID-19 deaths in California for the specified age group.</td></tr>
</table>

		working-age adults (18–64 years old) in California who died in 2020.			farming, fishing & forestry	332051	259	- The outcome is mortality, which may be influenced by other factors (e.g., pre-existing conditions)
		**Sample size:** 6 607		**Assessment:** 2010 Census code	material moving	506427	394	
		**Age:** Mean 50.7			construction and extrac-tion	1073718	670	
		**Time of outcome:** 01/2020 to 12/2020		**Time of occupation assessment:** 2020 (6 months before death)	production	1104651	665	
					transporta-tion	867133	496	
		**Response:** Register			installation, mainte-nance & re-pair	576087	318	
					building & grounds cleaning & mainte-nance	982942	461	
					food prepa-ration & serving re-lated	776087	357	
					protective service	415909	183	
Della Valle, 2021	Cohort	**Study region:**		**Investigated occupation:**				

<table>
<tr>
<td></td>
<td></td>
<td>Italy
Sample population: Employees from facilities in the Lombardy region
Sample size:
2 255
Age:
MW (SD) 44.5 (+-9.71)
Time of outcome:
05/2020 to 10/2020
Response: 75.8</td>
<td>Infection
(IgG, IgM)</td>
<td>diverse (n= 3 occupational sectors)
Assessment:
at work
Time of occupation assessment:
05/2020 to 07/2020</td>
<td><table><tr><td>Description</td><td>Total</td><td>IgG/ IgM antibod-ies</td></tr><tr><td>office worker</td><td>993</td><td>36</td></tr><tr><td>Police</td><td>1.213</td><td>67</td></tr><tr><td>HCW</td><td>49</td><td>6</td></tr></table></td>
<td>- Even if a lot covariates were available no adjusted model was calculated.
- Office worker were not further distinguished.</td>
</tr>
<tr>
<td>Diaz-Salazar, 2021</td>
<td>Cross-sectional</td>
<td>**Study region:**
Mexico
Sample population: City government Employees (Ciudad Guadalupe, NL, Mexico) voluntarily participated in a survey.
Sample size:
3 268
Age:
Md [IQR]=40 [31-49]</td>
<td>Infection
(IgG, IgM)</td>
<td>**Investigated occupation:**
diverse (n=5 occupational sectors)
Assessment:
register</td>
<td><table><tr><td>occupation</td><td>total</td><td>Positive IgG/ IgM</td></tr><tr><td>office / management</td><td>696</td><td>36</td></tr><tr><td>police, firefighters, public safety</td><td>1.051</td><td>69</td></tr><tr><td>maintenance / janitors</td><td>608</td><td>25</td></tr><tr><td>HCW</td><td>163</td><td>8</td></tr><tr><td>other</td><td>750</td><td>55</td></tr></table></td>
<td>- High response
- Participants were limited to government employees.
- Covers only one month</td>
</tr>
</table>

<table>
<tr>
<td></td>
<td></td>
<td>Time of outcome:
07/2020

Response:
77.4</td>
<td></td>
<td>Time of occupation assessment:
07/2020</td>
<td></td>
<td></td>
</tr>
<tr>
<td>Finkenzeller, 2020</td>
<td>Cross-sectional</td>
<td>Study region:
Deutschland

Sample population:
Study included 2 groups: a) Healthcare workers b) non-medical workers. Persons in b) were from large regional company.

Sample size:
2 867

Age:
MW (classes) = 42

Time of outcome:
07/2020

Response:
67.7</td>
<td>Infection (IgG, IgM)</td>
<td>Investigated occupation:
diverse (n=13 occupational sectors)

Assessment:
at work

Time of occupation assessment:
07/2020</td>
<td>
<table>
<tr><th></th><th>Total</th><th>AB +</th></tr>
<tr><td>KKH - Ärztliches Personal</td><td>221</td><td>30</td></tr>
<tr><td>KKH - Funktionsdienste</td><td>298</td><td>38</td></tr>
<tr><td>KKH - Pflegepersonal</td><td>710</td><td>177</td></tr>
<tr><td>KKH - Reinigungspersonal</td><td>46</td><td>11</td></tr>
<tr><td>KKH - Technikpersonal</td><td>63</td><td>4</td></tr>
<tr><td>KKH - Verwaltung</td><td>223</td><td>17</td></tr>
<tr><td>UTN - Fertigung</td><td>196</td><td>5</td></tr>
<tr><td>UTN - Home-Office</td><td>69</td><td>7</td></tr>
<tr><td>UTN - Küche</td><td>52</td><td>1</td></tr>
<tr><td>UTN - Service Baustelle</td><td>112</td><td>6</td></tr>
<tr><td>UTN - Service In House</td><td>404</td><td>17</td></tr>
<tr><td>UTN - Verwaltung</td><td>196</td><td>8</td></tr>
<tr><td>KKH - Ärztliches Personal</td><td>221</td><td>30</td></tr>
</table>
</td>
<td>- Excluded due to overlapping outcomes</td>
</tr>
</table>

Fogh, 2021	Cohort	Study region: Denmark<br>**Sample population:** The study invited 1.3 million Danish residents aged over 15 years to participate, selected randomly from the Civil Registration Sys<br>**Sample size:** 318 552<br>**Age:** Median 55<br>**Time of outcome:** 10/2020<br>**Response:** 24,5	Infection (IgG, IgM)	**Investigated occupation:** diverse (n= 6 occupational sectors)<br>**Assessment:** web-based-questionnaire<br>**Time of occupation assessment:** 09/2020	Work type / Sero neg. / Sero pos.:<br>office work / 83 401 / 538<br>other / 44 755 / 370<br>healthcare sector / 21 863 / 287<br>tradesman / 20 653 / 154<br>school / other education / 23 773 / 199<br>shop work / 9 103 / 78<br>nursing home / 5 768 / 57<br>home care / 3 827 / 52	- Low response<br>- Large sample size
Hawkins, 2021	Cohort	**Study region**: USA<br>**Sample population:** The study focused on working-age individuals (16–64 years) in	Mortality (death certificates)	**Investigated occupation:** diverse (n= 11 occupational category)	(blank) / N / Age-adjusted mortality rate	- Inclusion of race and ethnic<br>- Result adjusted for these co-variates could not be used (unsuitable control group in the adjusted model)

		Massachusetts who were part of the civilian workforce.			office & administrative support	398305	11.8		- Occupation was “usual occupation”
		**Sample size:** 3 381 336		**Assessment:** death certificate: "usual work"	healthcare support	93504	44.1		
		**Age:** 56,2			transportation & material moving	155767	37.2		
		**Time of outcome:** 03/2020 to 07/2020		**Time of occupation assessment:** 3/2020 to 07/2020	food preparing & serving related	185416	31.6		
		**Response:** register			building & grounds cleaning & maintenance	117520	31.1		
					production	125614	29.6		
					installation, maintenance & repair	72219	27.2		
					construction and extraction	148782	28.3		
					protective service	64029	25.4		

personal care & service	138887	23.6
arts, design, entertainment & media	75878	23.5
community & social services	71053	18.7
life, physical & social science	65959	14.3
sales and related	300503	13.9
healthcare practitioners & technical	243593	11.2
architecture & engineering	78091	13.7
business and financial operations	201861	8.7
legal	43419	8.2

					computer & mathematical	158040	5.7		
					management	416461	5.4		
					education, training & library	248337	4.3		
House, 2021	Retrospective Cohort	**Study region**: USA<br>**Sample population:** Hospitals from the RECOVER study that were located in Iowa.<br>**Sample size:** 582<br>**Age:** MD [IQR]=42 [29; 55]<br>**Time of outcome:** 03/2020 to 05/2020<br>**Response:** -	Infection, admission to ICU (any test result)	**Investigated occupation:** meat packers<br>**Assessment:** hospital documentation<br>**Time of occupation assessment:** 03/2020 to 05/2020	occupation	total	Infected	Adj. RR	- data were from an emergency department<br>- persons had to have typical COVID-19 symptoms (e.g., cough)<br>- concentration on the occupation meat packers
					Other	533	64	Ref.	
					Meat packing	49	36	2.37 [1.59; 3.53]	
IIAC,	Cohort	**Study region:**		**Investigated occupation:**					

2021		Great Britain	Mortality (death records: U07.1 and U07.2)	diverse (n= 88 occupational category)				
		**Sample population:** England & Wales				f - managers & directors in retail & wholesale	26.7 [16.7, 40.2]	
		Sample size: 7 961		**Assessment:** SOC 2010		f - residential, day & domiciliary care managers & proprietors	31.5 [17.9, 51.3]	
		**Age:** Not specified				f - shop keepers & proprietors: wholesale & retail	36 [18, 63.8]	
		**Time of outcome:** 03/2020 to 12/2020		**Time of occupation assessment:** 2019		f - nurses	24.5 [19.7, 29.4]	
						f - secondary education teaching professionals	21.2 [12.4, 33.2]	
		**Response:** 100				f - primary & nursery education teaching professionals	10 [5.4, 16.5]	
						f - senior professionals of educational establishments	25.2 [10.7, 47.6]	
						f - social workers	32.4 [20.7, 48.3]	
						f - national government administrative occupations	27.9 [18.1, 41.2]	
						f - local government administrative occupations	10.5 [4.9, 19.5]	
						f - book-keepers, payroll managers & wages clerks	11.9 [7.7, 17.6]	

					f - bank & post office clerks	24.1 [13.4, 39.9]	
					f - other administrative occupations n.e.c.	12.3 [9.3, 15.9]	
					f - office managers	8.7 [4.2, 15.9]	
					f - personal assistants and other secretaries	19.1 [12.7, 27.4]	
					f - receptionists	9.5 [5.3, 15.6]	
					f - chefs	40.2 [20.5, 70.0]	
					f - nursery nurses & assis-tants	11.8 [5.3, 22.0]	
					f - childminders & related occupations	27.8 [15.9, 44.8]	
					f - teaching assistants	15 [10.2, 21.0]	
					f - nursing auxiliaries & as-sistants	25.3 [18.9, 33.1]	
					f - house-parents & resi-dential wardens	37.4 [18.8, 65.7]	
					f - care workers and home carers	47.1 [44.1, 53.1]	
					f - hairdressers and bar-bers	44 [24.2, 72.2]	

					f - housekeepers & related occupations	26.4 [14, 45]	
					f - cleaning & housekeeping managers & supervisors	26.1 [12.8, 47.1]	
					f - sales and retail assistants	26.9 [21.8, 31.9]	
					f - retail cashiers & check-out operators	15.7 [8.4, 26.4]	
					f - customer service occupations n.e.c.	12.6 [7, 20.8]	
					f - food, drink and tobacco process operatives	28.2 [14, 50.6]	
					f - sewing machinists	64.8 [34.6, 110.1]	
					f - cleaners & domestics	21.5 [17.4, 26.3]	
					f - school midday & crossing patrol occupations	19.2 [11.3, 30.3]	
					f - kitchen & catering assistants	18.8 [13.1, 26.1]	
					m - production managers and directors in manufacturing	16.3 [11.7, 22.1]	
					m - production managers and directors in construction	20.7 [14.1, 29.4]	

m - financial managers and directors	10 [5.7, 16.2]
m - marketing and sales directors	8.2 [4.2, 14.1]
m - functional managers and directors n.e.c.	16.7 [7.8, 31.1]
m - managers and directors in transport and distribution	51.6 [33.8, 74.9]
m - managers and directors in storage and warehousing	49.9 [30.1, 76.7]
m - managers and directors in retail and wholesale	28.3 [20, 38.8]
m - restaurant & caring establishment: managers & proprietors	119.3 [71.2, 183.3]
m - publicans & managers of licensed premises	219.9 [124.7, 354.2]
m - property, housing and estate managers	31.1 [20.4, 45.4]
m - shop keepers & proprietors: wholesale & retail	69 [51.8, 90.1]

					m - managers and proprietors in other services n.e.c.	40.6 [28.9, 55.2]	
					m - IT specialist managers	21.9 [12, 35.7]	
					m - programmers and software development professionals	8 [3.9, 14]	
					m - information technology and telecommunication professionals n.e.c.	20 [11.9, 31.1]	
					m - medical practitioners	27.6 [18.6, 39.5]	
					m - nurses	79.1 [57.4, 106.1]	
					m - higher education teaching professionals	11.5 [5.2, 21.7]	
					m - further education teaching professionals	24.7 [11.1, 46.6]	
					m - secondary education teaching professionals	39.2 [24.3, 58.6]	
					m - chartered and certified accountants	21.9 [13, 34.4]	
					m - business and financial project management professionals	20.1 [10.7, 33.7]	
					m - chartered surveyors	21.6 [10.3, 39.9]	

					m - IT operations technicans	32.2 [15.3, 58.3]	
					m - police officers (sergeant and below)	194.1 [93.3, 336.3]	
					m - protective service associate professionals n.e.c.	39.3 [20.1, 68.3]	
					m - photographers, audio-visual and broadcasting equipment operators	31.9 [15.6, 57.1]	
					m - business and related associate professional n.e.c.	20.3 [9.7, 37]	
					m - business sales executives	47.2 [29.8, 70.6]	
					m - sales accounts and business development managers	15.8 [10.4, 22.9]	
					m - national government administrative occupations	58.5 [38.8, 84.7]	
					m - local government adminstrative occupations	72.1 [44.8, 109.4]	
					m - book-keepers, payroll managers and wages clerks	48.4 [33.7, 67.2]	

					m - bank & post office clerks	105.5 [49.6, 193.7]	
					m - transport and distribution clerks and assistants	51.4 [27.2, 87.1]	
					m - other administrative occupations n.e.c.	26.8 [17.4, 39.4]	
					m - farmers	17 [8.8, 29.4]	
					m - gardeners and landscape gardeners	22.2 [14.6, 32.3]	
					m - welding trades	54.7 [33.1, 83.5]	
					m - metal working production and maintenance fitters	36.4 [27.8, 46.8]	
					m - vehicle technicians, mechanics, electricians	58 [42.4, 77.4]	
					m - aircraft maintenance & related trades	70.8 [34.4, 128.2]	
					m - electricians and electrical fitters	33.3 [25, 43.5]	
					m - telecommunications engineers	56.4 [30.8, 93.7]	
					m - IT engineers	51.9 [25.9, 92]	
					m - electrical and electronic trades n.e.c.	38 [23.6, 57.9]	

					m - bricklayers and ma-sons	32.4 [18.9, 51.6]	
					m - roofers, roof tilers & slaters	100.5 [55.8, 163.6]	
					m - plumbers and heating and ventilating engineers	24.3 [16.3, 34.6]	
					m - carpenters and joiners	43.1 [32.8, 55.6]	
					m - construction and building trades n.e.c.	40.1 [32, 49.7]	
					m - plasterers	38.5 [18.9, 69.2]	
					m - painters and decora-tors	47 [34.9, 61.8]	
					m - butchers	207 [112.2, 346.8]	
					m - bakers & flour confec-tioners	715.6 [331. 1282.8]	
					m - chefs	103.1 [79.9, 130.5]	
					m - catering & bar manag-ers	86.8 [41.6, 155.4]	
					m - nursing auxiliaries & assistants	87.2 [63.3, 117.1]	
					m - ambulance staff (excl. Paramedics)	95.2 [38.7, 178.5]	

					m - care workers and home carers	109.9 [88.6, 131.3]	
					m - hairdressers & barbers	112.5 [49.6, 209.8]	
					m - caretakers	30.1 [19.4, 44.4]	
					m - sales & retail assis-tants	56.5 [43.7, 71.9]	
					m - retail cashiers & check out operators	61.6 [27.9, 114.7]	
					m - vehicle and parts salespersons and advisors	42.1 [20.3, 76.6]	
					m - customer service oc-cupations n.e.c.	41.8 [23.2, 68.8]	
					m - food, drink and to-bacco process operatives	103.7 [77.2, 136.4]	
					m - metal working ma-chine operators	106.1 [74.5, 146.0]	
					m - routine inspectors and testers	29 [15.8, 48.7]	
					m - construction opera-tives n.e.c.	23.7 [14.6, 36.3]	
					m - large goods vehicle drivers	39.7 [32.4, 47.1]	
					m - van drivers	39.7 [32.1, 48.5]	

					m - bus and coach drivers	70.3 [55.3, 88]	
					m - Taxi & cab drivers, chauffeurs	101.4 [87.5, 115.2]	
					m - fork-lift truck drivers	34.8 [21.4, 53.1]	
					m - mobile machine and operatives n.e.c.	44.2 [24.9, 72.3]	
					m - elementary construction occupations	82.1 [63.9, 103.7]	
					m - packers, bottlers, canners and fillers	51.6 [24.9, 91.4]	
					m - postal workers, mail sorter, messengers, couriers	58.2 [44.5, 74.6]	
					m - cleaners & domestics	66.6 [50.3, 86.5]	
					m - refuse and salvages occupations	45.8 [23.9, 79.2]	
					m - vehicle valets & cleaners	142.9 [60.7, 275.5]	
					m - Security guards & related	100.7 [83.8, 117.6]	
					m - elementary storage occupations	54 [43.4, 64.6]	
					m - hospital porters	86.7 [47.7, 142.3]	

					m - kitchen & catering assistants | 57 [38, 81.9]<br>m - waiters & waitresses | 95.7 [46.6, 169.1]	
Lastrucci, 2020	Cross-sectional	**Study region:**<br>Italy<br>**Sample population**:<br>included 4,656 participants from the Province of Prato, Tuscany, Italy<br>Sample size:<br>4 656<br>Age:<br>Md[IQR]= 49[38, 57]<br>Time of outcome:<br>05/2020<br>Response:<br>95,5	Infection (IgG, IgM)	**Investigated occupation:**<br>diverse (n=4 occupational sectors)<br>**Assessment:**<br>questionnaire<br>**Time of occupation assessment:**<br>05/2020	Type of service | total | positive | OR<br>work-from-home (administrative profiles) | 725 | 7 | 1<br>community support service | 502 | 5 | 1.03 [0.30-3.26]<br>local health authority support service | 601 | 10 | 1.73 [0.66-4.80]<br>health services | 2828 | 116 | 4.93 [2.19-10.43]	- logistic regression with 2 covariables (age, sex)<br>- High response<br>- Cross-sectional design
Magnusson, 2021	cohort	**Study region:**<br>Norway<br>**Sample population:**	Infection (PCR testing;	**Investigated occupation:**<br>diverse (n=22 occupational sectors)	occupation | TP |	- Included the entire working-age population of Norway

		The study included the entire Norwegian population of working-age adults (20–70 years)	ICD-10: U07.1)		individuals of working age_ZR1	1	Ref.		- Detailed occupational classification<br>- Temporal differences were investigated
					nurses_ZR1	1	2,736 [2,53; 2,96]		
					physicians_ZR1	1	2,378 [1,53; 3,69]		
		**Sample size:**<br>3 579 608		**Assessment:**<br>Register (Employer–Employee Register, which uses the Standard Classification of Occupations (STYRK-98))	dentists_ZR1	1	0,676 [0,58; 0,79]		
		**Age:**<br>Mean (Sd)= 44.1(14.3)			primary school teacher_ZR1	1	0,561 [0,42; 0,74]		
		**Time of outcome:**<br>02/2020-12/2020		**Time of occupation assessment:**<br>08/2020	early childhood education_ZR1	1	0,757 [0,67; 0,86]		
					childcare workers_ZR1	1	0,553 [0,41; 0,75]		
		**Response:**<br>Register			secondary education teachers_ZR1	1	0,86 [0,71; 1,04]		
					university and higher education teachers_ZR1	1	0,727 [0,65; 0,81]		
					shop sales assistant_ZR1	1	0,721 [0,62; 0,83]		
					cleaners_ZR1	1	0,635 [0,5; 0,81]		
					waiters_ZR1	1	0,829 [0,56; 1,23]		
					bartenders_ZR1	1	0,977 [0,72; 1,32]		

					food service counter at-tendant_ZR1	1	2,143 [1,76; 2,61]	
					bus and tram drivers_ZR1	1	1,315 [1,07; 1,62]	
					car, taxi and van drivers_ZR1	1	1,421 [0,95; 2,12]	
					physiothera-pists_ZR1	1	0,404 [0,23; 0,71]	
					hotel reception-ists_ZR1	1	0,28 [0,07; 1,12]	
					travel guides_ZR1	1	0,753 [0,31; 1,81]	
					travel attendants and travel stew-ards_ZR1	1	0,296 [0,042; 2,1]	
					transport con-ductors_ZR1	1	0,437 [0,25; 0,77]	
					fitness and recre-ation instructors and programme leaders_ZR1	1	0,418 [0,24; 0,74]	
					hair dress-ers_ZR1	1	2,736 [2,53; 2,96]	
					individuals of working age_ZR2	2	Ref.	

					nurses_ZR2	2	1,141 [0,88; 1,47]	
					physicians_ZR2	2	0,845 [0,71; 1]	
					dentists_ZR2	2	0,203 [0,029; 1,44]	
					primary school teacher_ZR2	2	1,142 [0,99; 1,32]	
					early childhood education_ZR2	2	0,727 [0,54; 0,99]	
					childcare work-ers_ZR2	2	1,145 [1,02; 1,29]	
					secondary educa-tion teach-ers_ZR2	2	1,095 [0,82; 1,47]	
					university and higher education teachers_ZR2	2	0,85 [0,68; 1,07]	
					shop sales assis-tant_ZR2	2	1,133 [1,03; 1,25]	
					cleaners_ZR2	2	0,857 [0,73; 1,01]	
					waiters_ZR2	2	1,873 [1,61; 2,18]	
					bartenders_ZR2	2	2,559 [2,05; 3,2]	
					food service counter at-tendant_ZR2	2	1,656 [1,31; 2,09]	

					bus and tram drivers_ZR2 | 2 | 1,162 [0,81; 1,66]<br>car, taxi and van drivers_ZR2 | 2 | 1,288 [1,02; 1,63]<br>physiotherapists_ZR2 | 2 | 0,801 [0,42; 1,54]<br>hotel receptionists_ZR2 | 2 | 1,03 [0,7; 1,52]<br>travel guides_ZR2 | 2 | 0,845 [0,35; 2,03]<br>travel attendants and travel stewards_ZR2 | 2 | 2,752 [1,59; 4,75]<br>transport conductors_ZR2 | 2 | 1,287 [0,41; 4]<br>fitness and recreation instructors and programme leaders_ZR2 | 2 | 1,134 [0,77; 1,67]<br>hair dressers_ZR2 | 2 | 1,144 [0,78; 1,69]	
Miller, 2021	Cross sectional	**Study region:** 3 739<br>**Sample population:** The study analysed farmworkers employed by a fruit	Infection (NAAT-antigen test)	**Investigated occupation:** farmworker (diverse)	| Total | Pos.<br>office | 49 | 3<br>forklift operator | 86 | 9<br>packing and sorting fruit | 304 | 84	- A specific working environment were investigated<br>- Detailed occupational classifications

		grower in Okanogan County, Washington-<br>**Sample size:** 3.739<br>**Age**: Not specified<br>**Time of outcome**: 05/2020 to 08/2020<br>**Response:** 75,5		**Assessment:** at work<br>**Time of occupation assessment:** 05/2020 to 08/2020	fruit packing support | 126 | 30<br>other warehouse (e.g., maintenance, cleaning, transportation) | 110 | 8<br>unknown job role | 51 | 36<br>orchard employees | 3.013 | 178	
Möhner, 2020	Cohort	Study region: Germany<br>Sample population: The study included more than 4.1 million employed individuals insured by the BARMER GEK health insurance in Germany.<br>Sample size: 4 100 000	Infection (ICD-10: U07.1, U07.2, U04.9, B34.2, B97.2)	**Investigated occupation:** diverse (n= 9 occupational sectors)<br>**Assessment:**	hospital_food production | 0.89 [0.43, 1.64]<br>hospital_energy/waste/water, cleaning sectors | 1.01 [0.69, 1.41]<br>hospital_sale of food, beverages, drugstore products, etc. | 0.98 [0.74, 1.27]<br>hospital_local public transport, inter-city railservices | 1.09 [0.50, 2.06]	- Large sample size<br>- Data were from 1 insurance company.<br>- No information of race, ethnicity, or socioeconomic status

		Age:<br>-		coded social security data (U07.1, U07.2, U04.9, B34.2, and B97.2)	hospital_postal services, logistics sector: 1.53 [1.05, 2.15]	
		Time of outcome:<br>01/2020 to 05/2020		**Time of occupation assessment:**<br>01/2020 to 05/2020	hospital_security services, law enforcement services: 1.75 [1.17, 2.51]<br>hospital_Employees with patient contact in hospitals and medical practices: 1.75 [1.56, 1.97]	
		Response:<br>register			hospital_Employees in the sector of providing and caring for the elderly: 2 [1.59, 2.47]<br>hospital_temporary agency work in production/logistics: 2.3 [1.67, 3.08]	
Nwaru, 2020	Cohort	**Study region:**<br>Sweden	Infection, hospitalization and ICU admission	**Investigated occupation:**<br>diverse (n= 8 occupational sectors)	Outcome type | occupation | N | HR	- comprehensive national data and a large sample size<br>- occupational status is from 2018-2019
		**Sample population:** The sample was derived from the SCIFI-PEARL project, a large				

		register-based co-hort.<br>**Sample size:**<br>326 052<br>**Age:**<br>ME(Sd)=43.0(12.5)<br>**Time of outcome:**<br>01/2020 to 02/2021<br>**Response:**<br>Register	(ICD-10:<br>U07.1, U07.2)	**Assessment:**<br>register<br>**Time of occupation assessment:**<br>2018-2019		non-essential workers	233957	Ref.	-	model were adjusted for age, gen-der, marital status, immigrant sta-tus, healthcare region, education and income
					infec-tion	healthcare workers	29233	1,92 [1.86, 1.99]		
					infec-tion	teachers	26642	1.44 [1.38, 1.50]		
					infec-tion	service sector workers	22957	1.1 [1.05, 1.15]		
					infec-tion	police and se-curity services	3230	n.s.		
					infec-tion	postal workers and delivery	1818	n.s.		
					infec-tion	taxi, bus, and tram drivers	2778	n.s.		
					infec-tion	cleaners	5437	n.s.		
						non-essential workers	233957	Ref.		
					Hospi-tal	healthcare workers	29233	1,7 [1.4, 2.1]		
					hospi-tal	teachers	26642	1.19 [0.9, 1.50]		

					hospi-tal	service sector workers	22957	0,98 [0.7, 1.3]	
					hospi-tal	police and se-curity services	3230	n.s.	
					hospi-tal	postal workers and delivery	1818	n.s.	
					hospi-tal	taxi, bus, and tram drivers	2778	n.s.	
					hospi-tal	cleaners	5437	n.s.	
						ICU_non-es-sential workers	233957	Ref.	
					ICU	ICU_healthcare workers	29233	1,8 [1.00, 3.10]	
					ICU	ICU_teachers	26642	1,06 [0.5, 2.2]	
					ICU	ICU_service sector workers	22957	0.82 [0.4, 1.9]	
					ICU	ICU_police and security ser-vices	3230	n.s.	

					ICU | ICU_postal workers and delivery | 1818 | n.s.<br>ICU | ICU_taxi, bus, and tram drivers | 2778 | n.s.<br>ICU | ICU_cleaners | 5437 | n.s.<br>n.s. … not specified<br>HR … hazard ratio	
Poustchi, 2021	Cross-sectional	**Study region:**<br>Iran<br>**Sample population:**<br>Participants were from 18 cities in Iran. There were 2 groups<br>a) persons randomly selected from health record (general population)<br>b) participants recruited from high-exposure jobs<br>**Sample size:**<br>8 902<br>**Age:**<br>Me=42.6<br>**Time of outcome:**<br>04/2020 to 06/2020	Infection (IgG, IgM)	**Investigated occupation:**<br>diverse (n=6 occupational sectors)<br>**Assessment:**<br>at work<br>**Time of occupation assessment:**<br>04/2020 to 06/2020	| N | seropositive<br>general population | 3.530 | 494<br>non-front-line HCW | 1.156 | 162<br>front-line HCW | 1.245 | 209<br>pharmacy employees | 620 | 101<br>taxi drivers | 718 | 101<br>cashiers of supermarket chains | 753 | 110<br>bank employees | 880 | 136	- Seroprevalence was weighted by age, sex, and city population size to ensure representativeness.<br>- No further information for occupation of general population is given.

		**Response:**<br>97				

<table>
<tr>
<td>Stringhini, 2021</td>
<td>Cross-sec-tional</td>
<td>Study region:
Switzerland
Sample population:
Workers from 16 economic sectors in Geneva, Switzerland, were invited to participate.
Sample size:
10 513
Age:
Me(Sd) = 43 (10.7)
Time of outcome:
05/2020 to 09/2020
Response:
41</td>
<td>Infection (IgG)</td>
<td>Investigated occupation:
diverse (n=16 occupational sectors)
Assessment:
at work
Time of occupation assessment:
05/2020 to 09/2020</td>
<td>
<table>
<tr><th>Activity Sector</th><th>N</th><th>Sero positive</th></tr>
<tr><td>public administration</td><td>1.056</td><td>94</td></tr>
<tr><td>healthcare</td><td>1.668</td><td>185</td></tr>
<tr><td>transportation</td><td>1.185</td><td>99</td></tr>
<tr><td>nursing homes</td><td>1.102</td><td>157</td></tr>
<tr><td>public security</td><td>1.055</td><td>83</td></tr>
<tr><td>food industry</td><td>755</td><td>76</td></tr>
<tr><td>homecare</td><td>753</td><td>91</td></tr>
<tr><td>social work</td><td>734</td><td>62</td></tr>
<tr><td>financial services</td><td>528</td><td>65</td></tr>
<tr><td>international organizations</td><td>425</td><td>24</td></tr>
<tr><td>early childhood education</td><td>259</td><td>15</td></tr>
<tr><td>pharmacy</td><td>254</td><td>28</td></tr>
<tr><td>construction</td><td>182</td><td>11</td></tr>
<tr><td>media</td><td>166</td><td>7</td></tr>
<tr><td>agriculture</td><td>97</td><td>8</td></tr>
<tr><td>other</td><td>294</td><td>21</td></tr>
<tr><td>public administration</td><td>1.056</td><td>94</td></tr>
<tr><td>healthcare</td><td>1.668</td><td>185</td></tr>
</table>
</td>
<td>- In the original paper, the occupations were compared with healthcare, so the adjusted estimators could not be used because healthcare was an ineligible comparison group
- Moderate participation rates</td>
</tr>
<tr>
<td>Stufano, 2021</td>
<td>Cross-sec-tional</td>
<td>Study region:
Italy
Sample population:
study population consisted of inmates and</td>
<td>Infection (rRT-PCR)</td>
<td>Investigated occupation:
correctional workers (diverse)</td>
<td>
<table>
<tr><th></th><th>cam-paign</th><th>Total</th><th>positive</th></tr>
</table>
</td>
<td>- The occupational group studied were all those working in prison.</td>
</tr>
</table>

		correctional workers in an Italian prison.<br>**Sample size:** 367<br>**Age:** -<br>**Time of outcome:** 11/2020 to 01/2021<br>**Response:** 91.3		**Assessment:** at work<br>**Time of occupation assessment:** 11/2020 to 01/2021	adminis-trative staff | first | 66 | 0<br>HCW | first | 77 | 2<br>correc-tional of-ficers | first | 216 | 4<br>external workers | first | 8 | 0<br>adminis-trative staff | second | 49 | 0<br>HCW | second | 47 | 0<br>correc-tional of-ficers | second | 202 | 0<br>external workers | second | 4 | 0	- Just workers from 1 prison were investi-gated.
Tovar, 2021	Cross-sec-tional	**Study region:** Peru<br>**Sample population:** The study population consisted of workers at the market and workers at the bus depot.<br>**Sample size:** 1 773<br>**Age:**	Infection (IgG, IgM)	**Investigated occupation:** diverse (n=5 occupational sectors)<br>**Assessment:** at work	| N | positive<br>bus depot admin | 62 | 20<br>bus depot driver | 329 | 80<br>bus depot cook/ | 52 | 25	none

		-1285 vendors median age [interquartile range]: 40 [30–51] years<br><br>- 488 workers (bus depot, drivers etc.) median age: 39 [32–48] years<br><br>**Time of outcome:**<br>06/2020 to 07/2020<br><br>**Response:**<br>92.9		**Time of occupation assessment:**<br>06/2020 to 07/2020	cleaner/ security | | bus depot maintenance | 34 | 11<br>market vendors | 1.285 | 565	
Verbeck, 2021	Cohort	**Study region:**<br>Belgium<br>**Sample population:** All employees covered under the Belgian social security scheme were included (Self-employed individuals not included).<br>**Sample size:**<br>4 390 750<br>**Age**:<br>-	Infection (register confirmed cases)	**Investigated occupation:**<br>diverse (n= 18 occupational sectors)<br><br>**Assessment:**<br>database of active employees (NACE-BEL)	Results<br><br>Description | P | Employees | Incidence<br>general population | 1 | | 423<br>sports activities, amusement and recreation activities | 1 | 26.911 | 955	- Occupational sectors with at least 10,000 employees were included in detailed analyses.<br>- Large data set

		**Time of outcome:**<br>09/2020 to 10/2020		**Time of occupation assessment:**<br>09/2020 to 10/2020	human health activities	1	261.154	780	
		**Response:**<br>100			creative, arts and entertainment activities	1	21.922	739	
					food and beverage service activities	1	191.978	723	
					residential care activities	1	162.464	698	
					accommodation	1	27.454	652	
					activities of membership organisations	1	47.887	639	
					activities auxiliary to financial services and insurance activities	1	30.351	626	

security and investiga-tions activity	1	20.096	622
office ad-ministrative, office sup-port and other busi-ness support activities	1	36.731	618
sports activi-ties	1	21.131	1.008
other resi-dential care activities	1	15.412	837
hospital ac-tivities	1	210.745	819
hotels and similar ac-commoda-tion	1	20.076	792
residential care activi-ties for the elderly and disabled	1	66.667	786

					restaurants and mobile food service activities	1	149.471	756		
					activities of call centres	1	10.133	750		
					creative, arts and enter-tainment ac-tivities	1	21.922	739		
					other pas-senger land transport	1	40.751	719		
					medical and dental prac-tice activities	1	22.695	705		
					activities of sports clubs	1	5.954	1.394		
					other human resources provision	1	4.320	1.250		
					fitness facili-ties	1	3.707	1.187		
					other retail sale of food in special-ised stores	1	3.312	1.117		

						other sports activities	1	3.220	1.087		
						performing arts	1	5.196	1.020		
						other amusement and recrea-tion activi-ties	1	3.398	883		
						manufacture of air and spacecraft and related machinery	1	5.257	875		
						other resi-dential care activities	1	15.412	837		
						service activ-ities inci-dental to air transporta-tion	1	5.968	821		
						activities of football clubs	1	3.605	1.609		
						activities of leagues and	1	1.961	1.428		

					sports federations					
					other human resources provision	1	4.320	1.250		
					fitness facilities	1	3.707	1.187		
					other retail trade of food in specialised stores	1	2.857	1.155		
					general ordinary secondary education	1	159.055	1.143		
					general construction of office buildings	1	1.756	1.139		
					general social services with accommodation	1	2.500	1.080		
					production of shows by	1	4.924	1.056		

					artistic ensembles				
					other cleaning activities	1	2.846	1.019	
					general population	2		816	
					sports activities, amusement and recreation activities	2	26.687	1.660	
					human health activities	2	261.680	1.631	
					residential care activities	2	162.476	1.543	
					food and beverage service activities	2	189.940	1.342	
					public administration and defence; compulsory social security	2	542.998	1.321	

					security and investiga-tions activity	2	20.015	1.304	
					education	2	534.158	1.294	
					activities auxiliary to financial ser-vices and in-surance ac-tivities	2	30.322	1.273	
					real estate activities	2	24.582	1.257	
					office ad-ministrative, office sup-port and other busi-ness support activities	2	37.037	1242	
					sports activi-ties	2	21.062	1.752	
					residential care activi-ties for the elderly and disabled	2	66.609	1.746	

					hospital activities	2	210.960	1.688		
					other residential care activities	2	15.472	1.577		
					medical and dental practice activities	2	22.765	1.454		
					office administrative and support activities	2	11.777	1.435		
					secondary education	2	404.968	1.409		
					residential care activities for mental retardation, mental health and substance abuse	2	39.972	1.406		
					beverage serving activities	2	19.500	1400		
					activities of call centres	2	10.367	1389		

					other human resources provision	2	4.326	2.381		
					activities of sports clubs	2	5.875	2.349		
					other sports activities	2	3.208	1.964		
					child day-care activi-ties	2	25.658	1.824		
					other retail sale of food in special-ised stores	2	3.305	1.785		
					fitness facili-ties	2	3.741	1.764		
					residential care activi-ties for the elderly and disabled	2	66.609	1.746		
					other credit granting	2	3.230	1.734		
					manufacture of air and spacecraft	2	5.251	1.714		

and related machinery			
public order and safety activities	2	53.021	1.688
manufacture of weapons and ammunition	2	2.183	2.657
general construction of office buildings	2	1.761	2.556
general ordinary secondary education	2	159.806	2.478
other human resources provision		4.326	2.381
activities of football clubs	2	3.556	2.250
activities of leagues and sports federations	2	1.956	2.250

					<table><tr><td>distribution of gaseous fuels through mains</td><td>2</td><td>1.881</td><td>2.126</td></tr><tr><td>activities of medical laboratories</td><td>2</td><td>5.159</td><td>1.977</td></tr><tr><td>nurseries and day-care centres</td><td>2</td><td>22.564</td><td>1.950</td></tr><tr><td>motion picture projection activities</td><td>2</td><td>1.633</td><td>1.837</td></tr></table><br>P … Period<br>1 … 29.09-12.10<br>2 …06.10-19.10	
Ward, 2021	Cohort	**Study region:** USA<br>**Sample population:** U.S. correctional staff from state and federal prisons<br>**Sample size:** 386 311<br>Age:	Infection (PCR)	**Investigated occupation:** prison officers<br>**Assessment:** register	<table><tr><td></td><td>Time Period</td><td>Cases / 100.000</td></tr><tr><td>General population</td><td>03/20 to 05/20</td><td>385</td></tr><tr><td>Prison staff</td><td>03/20 to 05/20</td><td>1469</td></tr></table>	- Included correctional staff from nearly all U.S. states<br>- Variability in case reporting protocols across states

<table>
<tr>
<td></td>
<td></td>
<td>-

Time of outcome:
03/2020 to 11/2020

Response:
100</td>
<td></td>
<td>

Time of occupation assessment:
2019-2020</td>
<td><table><tr><td>General population</td><td>03/20 to 11/20</td><td>2900</td></tr><tr><td>Prison staff</td><td>03/20 to 11/20</td><td>9316</td></tr></table></td>
<td></td>
</tr>
<tr>
<td>Zhang, 2021</td>
<td>cohort</td>
<td>**Study region:**
USA

Sample population:
Case counts were published by the Washington State Department of Health.

Sample size:
10 850

Age:
-

Time of outcome:
02/2020 to 06/2020

Response:
Register (41%, 26 799 cases, only 10 850</td>
<td>Infection (lab confirmed)</td>
<td>**Investigated occupation:**
diverse (n=22 occupational sectors)

Assessment:
SOC 2010

Time of occupation assessment:
05/2019</td>
<td><table><tr><td></td><td>employees</td><td>cases</td><td>Case per 100.000 employees</td></tr><tr><td>office and administrative support</td><td>392.860</td><td>695</td><td>176,91</td></tr><tr><td>architecture and engineering</td><td>77.020</td><td>97</td><td>125,94</td></tr><tr><td>arts, design, entertainment, sports, and media</td><td>49.860</td><td>93</td><td>186,52</td></tr><tr><td>building and</td><td>90.590</td><td>579</td><td>639,14</td></tr></table></td>
<td>- no real-time occupational data were available, occupation time were from 2019
- only 41% of COVID-19 cases had occupational information</td>
</tr>
</table>

		with occupational information)			grounds cleaning and maintenance				
					business and financial operations	225.940	203	89,85	
					community and social service	52.280	178	340,47	
					computer and mathematical	173.940	111	63,82	
					construction and extraction	169.600	606	357,31	
					education, training, and library	189.670	241	127,06	
					farming, fishing, and forestry	22.250	741	3.330,34	
					food preparation	299.950	517	172,36	

and serving related			
healthcare practitioners and technical	171.440	1.208	704,62
healthcare support	144.170	989	686,00
installation, maintenance, and repair	133.320	240	180,02
legal	22.500	49	217,78
life, physical, and social sciences	39.850	54	135,51
management	162.850	667	409,58
personal care and service	74.900	579	773,03
production	178.980	964	538,61
protective service	66.690	231	346,38

					sales and related	316.510	712	224,95	
					transporta-tion and material moving	263.330	1.096	416,21	
